# Prospective Characterization Factors for Assessing Climate Change Impacts in Life Cycle Assessments

**DOI:** 10.1021/acs.est.5c12391

**Published:** 2026-01-21

**Authors:** Marcos D. Barbosa Watanabe, Francesco Cherubini

**Affiliations:** Industrial Ecology Programme, Department of Energy and Process Engineering, 8018 Norwegian University of Science and Technology (NTNU), Trondheim 7034, Norway

**Keywords:** prospective life cycle assessment, characterization factors, integrated assessment models, climate impacts, life cycle impact

## Abstract

Prospective life cycle assessment (pLCA) is a future-oriented approach that estimates the environmental impacts of products and systems under future technological changes, market dynamics, and policy shifts. However, pLCA lacks consistent prospective characterization factors (pCFs) to assess the climate impacts of future emissions and align the inventory and impact assessment phases. This work produces pCFs by integrating gas-specific climate parameters with future emission scenarios from the Integrated Assessment Models (IAM). Prospective Global Warming Potential (pGWP_20_, pGWP_100_) and Global Temperature change Potential (pGTP_50_, pGTP_100_) are computed for emission years until 2050. Relative to present-day CFs, methane pGWP_100_ varies from −8% to +23%, and nitrous oxide varies from −17% to +7%. CH_4_ pGTP_100_ shifts from −24% to +22%, while N_2_O pGTP_100_ shifts from −27% to +8%. For non-CO_2_-dominated activities such as rice production, climate impacts increase by 8% in terms of pGWP_100._ With pGTP_100_, impacts of ammonium nitrate decrease by 9%. When pCFs are combined with prospective background inventories, impacts are substantially lower in sectors such as steel (−44%), road transport (−58%), and cement (−31%) under pGTP_100_. Overall, the availability of pCFs for multiple climate metrics and IAM scenarios enables a consistent coupling of impact assessment with future-oriented inventory data, improving the robustness and coherence of pLCA.

## Graphical abstract

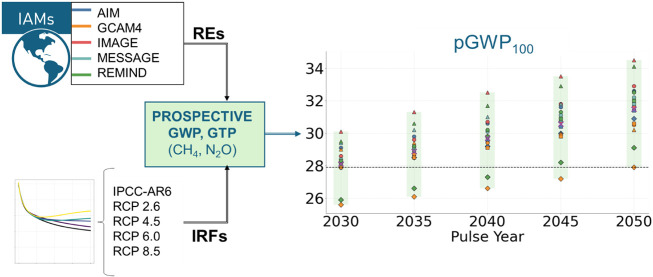

## 1 Introduction

Prospective life cycle assessment (pLCA) is an emerging methodology designed to evaluate the future environmental impacts of technologies and their associated products.^1^ In recent years, the application of pLCA has grown significantly,^2^ triggering improved definitions and methodological frameworks to increase consistency and transparency in comparative assessments.^3–6^ Despite these advancements, progress across the quantitative stages of pLCA has been uneven. While several methods have been developed to advance the Life Cycle Inventory (LCI) phase, the life cycle impact assessment (LCIA) phase notably remains underdeveloped.^3^ Addressing this gap is critical, as unresolved limitations in LCIA can undermine an accurate and consistent evaluation of environmental burdens, potentially leading to incomplete or misleading conclusions.^7,8^

In LCI, developments entailed both foreground and background system processes. Many studies explored approaches to scale-up foreground inventories to assess emerging technologies within a more realistic industrial-scale context,^9^ using methods based on process-based calculations,^10^ learning curves and scaling factors,^9,11,12^ expert elicitation through interviews,^11,13^ and the integration of technical parameters with assumptions on efficiency improvements.^1^ In parallel, aligning background inventories with projections from Integrated Assessment Models (IAMs) has emerged as a crucial step in recent years to improve pLCA studies and make them more consistent among each other and with the main future global policy scenarios.^14–20^ Scenario-based modeling in IAMs captures possible future developments in technology and emissions, and it offers a structured and scientifically robust foundation for defining key parameters in pLCAs. The further development of the open-source tool *Premise* ^21^ consolidated this approach into a transparent and reproducible framework for generating background databases among practitioners.

Despite these advances in modeling future LCIs, the corresponding progress in the LCIA phase has not occurred at the same pace or depth. CFs are based on present-day environmental conditions and are assumed to remain constant when used in pLCA, even if these conditions change in the future. For example, CFs used to estimate climate change impacts in LCA rely on climate metrics computed using radiative efficiencies and impulse response functions that reflect the current atmospheric concentration of GHGs and the saturation levels of terrestrial and ocean carbon sinks.^22^ Using these CFs to characterize GHG emissions occurring far in the future is therefore inconsistent as the background conditions of the atmosphere receiving an emission in the future may substantially differ from those on which the CFs are originally based.

Existing studies on time-dependent CFs for climate change impacts are limited, as most of the discussions focus on dynamic rather than prospective LCIA aspects.^23^ A few examples that are relevant in the context of the prospective analysis exist. Reisinger et al. (2011)^24^ linked Representative Concentration Pathways (RCPs) to projections of Global Warming Potentials (GWPs).^25,26^ Although not explicitly connected to the LCA field and to the need for pCFs, this work shows how future background concentrations of GHGs influence radiative efficiencies (RE) and climate–carbon cycle feedbacks, thereby affecting the GWPs of methane (CH_4_) and nitrous oxide (N_2_O). The findings suggest potential increases in GWP of up to 20% for CH_4_ and approximately 30% for N_2_O by 2100. However, a direct applicability of these metrics as CFs in LCA is hindered by several barriers, such as the lack of integration with IAM-based scenarios, the use of complex and nonopen-source climate models, the challenge to adjust metric values to the specific year at which emissions occur, a lack of regular updates, and the absence of alternative metrics such as the Global Temperature change Potential (GTP).^27,28^ More recent work provided new projections of climate metrics for CH_4_ and N_2_O by incorporating changes in RE in line with only RCP scenarios.^29^ However, these results follow a different trajectory than that reported by Reisinger et al. (2011),^24^ do not incorporate IAM-based prospective scenarios, and do not consider other climate indicators such as GTP.

In this study, we address the gap of missing CFs for climate change impacts in pLCA by proposing an approach that is consistent with the method used by the IPCC to compute climate metrics, such as GWP and GTP. RE and impulse response functions (IRFs) for the three most important GHGs (CO_2_, CH_4_, and N_2_O) are adapted to future changes in background concentrations as those underpinning IAM scenario outputs that are used to modify background process inventories. The study considers IRFs for CO_2_ that are specific to each main RCP scenario and relies on the most updated parameters from the IPCC Sixth Assessment Report^26^ to estimate changes in RE, and hence CFs, for methane (CH_4_) and nitrous oxide (N_2_O). Prospective metrics for both GWP (pGWP_100_ and pGWP_20_) and GTP (pGTP_100_ and pGTP_50_) are computed for emissions of GHGs occurring between 2030 and 2050. To facilitate their adoption from pLCA studies and tools, the pGWPs and pGTPs are provided in tabular form for emission years up to 2050 and open-access code is available to reproduce the method to compute pCFs within different modeling frameworks and to generate values through 2100. This approach extends beyond previous efforts by incorporating the full range of Shared Socioeconomic Pathways (SSPs) and enabling the calculation of year-specific metrics for any future pulse emissions. The new pCFs are applied to a range of case studies to explore how much and under which circumstances they can affect LCA results, either in isolation (i.e., relative to the use of existing CFs) or within a full pLCA framework that includes both pCFs and changes in background process inventories. This investigation also shows how the choice of IAM type can influence results, even under the same climate policy scenario.

## 2 Materials and Methods

### 2.1 Prospective CFs

#### 2.1.1 Prospective GWP (pGWP)

The method to estimate pCFs follows the equations and definitions of climate metrics that are used by the IPCC in its series of periodical assessments.^25,26,30^ The GWP is formulated as

1 $$GWP_{i}\left(H\right)=\frac{AGWP_{i}\left(H\right)}{AGWP_{CO_{2}}\left(H\right)}=\frac{\int_{0}^{H}RF_{i}\left(t\right)dt}{\int_{0}^{H}RF_{CO_{2}}\left(t\right)dt}$$

where AGWP_*i*_ (*H*) is the absolute global warming potential associated with a pulse emission of gas *i* for the time horizon *H* (year), which is computed as the integrated radiative forcing (RF_*i*_) exerted by the gas *i* until *H*. The denominator denotes the same but for CO_2_, which is the gas used as reference (hence the characterized results are expressed in CO_2_-equivalents). RF_*i*_ is, in turn, described by eq 2:

2 $$RF_{i}=RE_{i}\cdot IRF_{i}$$

where RE_*i*_ is the radiative efficiency of gas *i* and IRF_*i*_ is the impulse response function, which indicates the fraction of species *i* remaining in the atmosphere over time after a pulse emission. The RE of a greenhouse gas is defined as the instantaneous radiative forcing per unit mass or concentration increase of that gas, and it is expressed in terms of watts per square meter per ppm increase or kg emission (W m^–2^ ppm^–1^ or W m^–2^ kg^–1^). For CO_2_, the RE_*i*_ values can be calculated through a logarithmic relationship with CO_2_ concentration^31^ in eq 3:

3 $${\mathrm{RF}}_{i}=5.35\cdot \ln\left(C/C_{0}\right)$$

Where *C* is the new CO_2_ concentration and *C*_0_ is the reference concentration. Alternatively, they can be estimated from the derivative of radiative forcings:^25^

4 $$RE_{i}=\frac{dRF_{i}}{dC_{i}}$$

where RF_*i*_ is the radiative forcing (in W m^–2^) and *C*_*i*_ is the concentration of species in the atmosphere (ppb). Considering the time-dependent nature of RE_*i*_ and *C_i_*, it is possible to project time-specific RE_*i*_ (in W·m^–2^·ppb^–1^) for CO_2_, CH_4_, and N_2_O. To secure consistency with the prospective inventories, the projections for RE are produced according to eqs 5, 6, and 7. RE_*i*_ are extracted from changes in both radiative forcings and GHG concentrations from the outputs of IAM-SSP marker scenarios, based on time steps for the period 2030–2050 ^32^ as follows:

5 $$RE_{CO_{2}}\left(t\right)=\frac{dRF_{CO_{2}}}{dC_{CO_{2}}}\approx \frac{RF_{CO_{2}}\left(t\right)-RF_{CO_{2}}\left(t^{\prime }\right)}{C_{CO_{2}}\left(t\right)-C_{CO_{2}}\left(t^{\prime }\right)}$$

6 $$RE_{CH_{4}}\left(t\right)=\frac{dRF_{CH_{4}}}{dC_{CH_{4}}}\approx \frac{RF_{CH_{4}}\left(t\right)-RF_{CH_{4}}\left(t^{\prime }\right)}{C_{CH_{4}}\left(t\right)-C_{CH_{4}}\left(t^{\prime }\right)}$$

7 $$RE_{N_{2}O}\left(t\right)=\frac{dRF_{N_{2}O}}{dC_{N_{2}O}}\approx \frac{RF_{N_{2}O}\left(t\right)-RF_{N_{2}O}\left(t^{\prime }\right)}{C_{N_{2}O}\left(t\right)-C_{N_{2}O}\left(t^{\prime }\right)}$$

Where *t* denotes a given year and *t′* represents the preceding time step from the IAM. Equations 5, 6, and 7 follow the average RE approach to align with the IPCC method to estimate GWP and GTP, which are derived from the marginal formulation (eq 4) using finite differences of RF and concentration over 5-year intervals. This interval reflects the temporal resolution of publicly available IAM outputs and therefore provides a numerical approximation of the marginal efficiency. To derive annualized results, the RE values calculated at each five-year step are linearly interpolated, providing a continuous one-year time series suitable for finer-resolution analyses. Our paper focuses on the 2030–2050 period to ensure a readable size of figures and tables, and because 2050 is a key target year for the time frame of many of the existing sustainable policies and mitigation goals. However, the codes and data provided in the Supporting Information (SI) file allow users to compute prospective characterization up to 2100.

In line with the IPCC method, the IRF for CO_2_ is based on Joos et al. (2013).^22^ The default climate metrics provided by the IPCC use IRF parameters of a multimodel mean based on the atmospheric background CO_2_ concentration in 2010 of 389 ppm (and thereby held constant in the calculation of the metric values). IRFs are also available for pulse emissions in 2100 under different future scenarios of background concentrations: 421 (RCP 2.6), 538 (RCP 4.5), 670 (RCP 6.0), and 936 ppm (RCP 8.5). However, while the standard IRF of CO_2_ typically used in GWP calculations is derived from a multimodel ensemble of 16 Earth System Models (ESMs),^22^ the ones for future conditions are produced from the Bern3D-LPJ model^33,34^ only. To overcome this inconsistency, we apply a delta function to reproduce the relative differences between the Bern3D-LPJ $IRF_{CO_{2}}$ and the multimodel $IRF_{CO_{2}}$ observed at 2010’s concentration to IRF curves under varying background atmospheric CO_2_ concentrations. This enables us to estimate future $IRF_{CO_{2}}$ for different RCP scenarios that can be used to estimate climate metrics to be used as pCFs maintaining consistency with the IPCC framework. For the non-CO_2_ GHGs, the IRF is modeled as a simple exponential decay based on the atmospheric lifetime of each gas (11.8 years for CH_4_ and 109 years for N_2_O) according to the IPCC-AR6.^26^ These lifetimes have remained relatively stable over time: for CH_4_ (and N_2_O), they were 12 (and 114) years in the 2001 IPCC-TAR,^35^ 12 (and 114) in the 2007 IPCC-AR4,^30^ and 12.4 (and 121) in the 2013 IPCC-AR5,^25^ despite increases in atmospheric concentrations of these gases (from 1,750 to 1,896 ppb for CH_4_ and from 316 to 335 ppb for N_2_O). Therefore, the IRFs for these gases are less sensitive to changes in background concentrations than those of CO_2_ and can be considered to remain constant.

The time-varying IAM-RCP-specific REs derived from eqs 5, 6, and 7 are input into eq 2. This equation is then combined with the RCP-specific IRF curves to derive the AGWPs and the pGWPs, as described in eq 1. In the AGWPs, the carbon cycle responses and other indirect contributions from both CH_4_ and N_2_O are also included by using parameter values from IPCC-AR6.^26^ These include carbon cycle feedbacks, CH_4_-induced effects on tropospheric ozone (O_3_) and stratospheric water vapor, as well as the indirect effects of N_2_O on stratospheric O_3_ and the lifetime of CH_4_. Although AGWPs are calculated by using time-varying RE over the integration horizon, our approach remains consistent with the GWP definition adopted by the IPCC, where the impact of a given pulse emission is assessed in an atmosphere with constant background concentrations and a steady-state climate system. This means that for a given pulse year (e.g., 2040), the RE value from that year is used and then held constant throughout the entire integration horizon.

#### 2.1.2 Prospective GTP (pGTP)

The absolute global temperature change potentials (AGTPs) associated with the pulse emission of gas *i* are based on eq 8:^27,28^

8 $$AGTP_{i}\left(H\right)=\int_{0}^{H}AGWP_{i}\left(\tau \right)\;R\left(H-\tau \right)\;d\tau$$

where *H* denotes the time horizon at which the temperature change is evaluated, and *τ* represents earlier times from 0 to *H*. The term *R*(*H* – *τ*) captures the climate system’s response at time *H* to a forcing that occurred at time *τ*. The temperature responses to changes in RFs are modeled using the framework established in the IPCC-AR6.^26^ Our approach considers a two-layer energy balance model, representing the surface layer (comprising the upper ocean and atmosphere) and the deep ocean layer. This model incorporates key parameters such as the efficacy of deep ocean heat uptake, the surface–deep ocean heat exchange rate, and the radiative damping coefficient. To facilitate the reproducibility of the results, our calculations build on the modeling framework developed by Persson and Johansson (2022),^36^ which reflects the equations and parametrizations used for estimating GHG metrics in the latest IPCC-AR6.^26^

After computing the AGTPs based on IAM-specific emission trajectories, we derive the prospective global temperature change potentials (pGTPs) for CH_4_ and N_2_O using eq 9:

9 $$GTP_{i}\left(H\right)=\frac{AGTP_{i}\left(H\right)}{AGTP_{CO_{2}}\left(H\right)}$$

While GWP compares the integrated RF of a pulse emission of a given species relative to that of CO_2_ over a specified time horizon,^37^ GTP compares the resulting temperature change at a specific point in time from a pulse emission to that caused by an equivalent pulse of CO_2_. The GTP accounts for both the time-dependent radiative forcing of different species and the temporal response of the climate system’s temperature. Therefore, the GTP reflects changes at the end of the chosen time horizon, whereas the GWP represents the integrated impacts over that period.^28^ As noted by Tanaka et al. (2019),^38^ emission metrics are also sensitive to the selected time scale, particularly for species whose atmospheric lifetimes differ substantially from that of CO_2_. For instance, CO_2_ can persist in the atmosphere for centuries or even millennia,^22^ whereas CH_4_ largely disappears within several decades after emission.^39^ As noted by Allen et al. (2016),^40^ the GWP_100_ of CH_4_ is approximately equivalent to GTP_40_, indicating that the cumulative radiative forcing over 100 years has a climate impact similar to the instantaneous temperature increase observed 40 years after an emission pulse. This means that GWP_100_ can be interpreted as a metric to assess temperature impacts after about 40 years (midterm impacts), while GTP_100_ assesses impacts after 100 years (long-term impacts). GWP_20_ or GTP_50_ are instead more suitable to evaluate impacts in the short term. Because of these complementary perspectives, using multiple metrics and time horizons has been recommended to better capture how impacts change under different temporal dimensions.^40–42^ No single climate metric can fully represent the temporal profile of the climate response to emissions of GHGs with varying lifetimes, making a multimetric approach essential to disentangle short- and long-term impact dynamics.

### 2.2 Sensitivity to Prospective CFs

We assess the sensitivity of climate change impacts to pCFs in two steps. First, the analysis starts with two representative products extracted from the ecoinvent 3.9 database:^43^ production of nonbasmati rice in India (IN) and nitric acid in 50% solution state from the European market (RER w/o RU). These processes are selected due to their relatively high emission factors of non-CO_2_ GHGs, particularly N_2_O and CH_4_. The analysis is executed with Brigthway2 after loading the new sets of IAM-specific CFs for climate change impact assessment presented above (pGWP_100_, pGTP_100_, pGWP_20_, and pGTP_50_). For simplification, a *pLCA* limited to the year 2040 is considered in this paper. Although the set of new CFs enables more detailed dynamic-prospective analyses (i.e., considering the specific years at which emissions will occur in the future), selecting a single pulse year better isolates the sensitivity of climate impacts from different product systems to the new CFs. Because of the differences in functional units and product systems, we further normalize the comparison of variability in characterized results to the percentage difference relative to the case in which the present-day CFs from IPCC-AR6 ^26^ are used. The second part aims to extend the approach to cover a broader group of activities from ecoinvent 3.9 ^43^ including power generation, transport, food, materials, and chemicals production.

### 2.3 Application of Prospective LCA to Both Inventories and CFs

The combination of evolving background systems and changes in climate change CFs is jointly investigated to explore the effects of integrating the prospective LCI and LCIA phases. Background transformations were implemented for the year 2040 using the Premise tool (version 2.2.7 ^21^), which modifies the ecoinvent database to reflect future conditions projected by IAMs. As only the REMIND-MAgPIE model provided projected databases for more than one RCP scenario in the Premise tool (RCP 2.6 and RCP 8.5), our analysis was limited to this IAM and these two scenarios. In addition to transforming background databases, Premise also introduces changes in foreground systems of sectors, such as power generation, cement, steel, transport, fuels, and heat production. As a result, the IAM-based projected technological improvements directly modified the foreground inventories for the following activities: electricity production from natural gas using combined cycle power plants; Portland cement production; unalloyed steel production via a converter; and the market for freight transport by unspecified lorry.

Climate impacts were recalculated using pCFs, and the results were expressed as percentage deviations from a reference case based on present-day characterization factors from IPCC-AR6 ^26^ and the unmodified ecoinvent 3.9 database.^43^ Activities were then ranked according to their sensitivity to these changes.

## 3 Results

### 3.1 Prospective AGWP and AGTP

The evolution of prospective REs, IRFs, AGWPs, and AGTPs is shown in Figure 1 for the different RCPs connected to the REMIND-MAgPIE-SSP5 scenario. Results for IMAGE-SSP1, MESSAGE-GLOBIOM-SSP2, AIM-CGE-SSP3, and GCAM4-SSP4 are presented in Figures S1–S30 in the SI.

**Figure 1:**
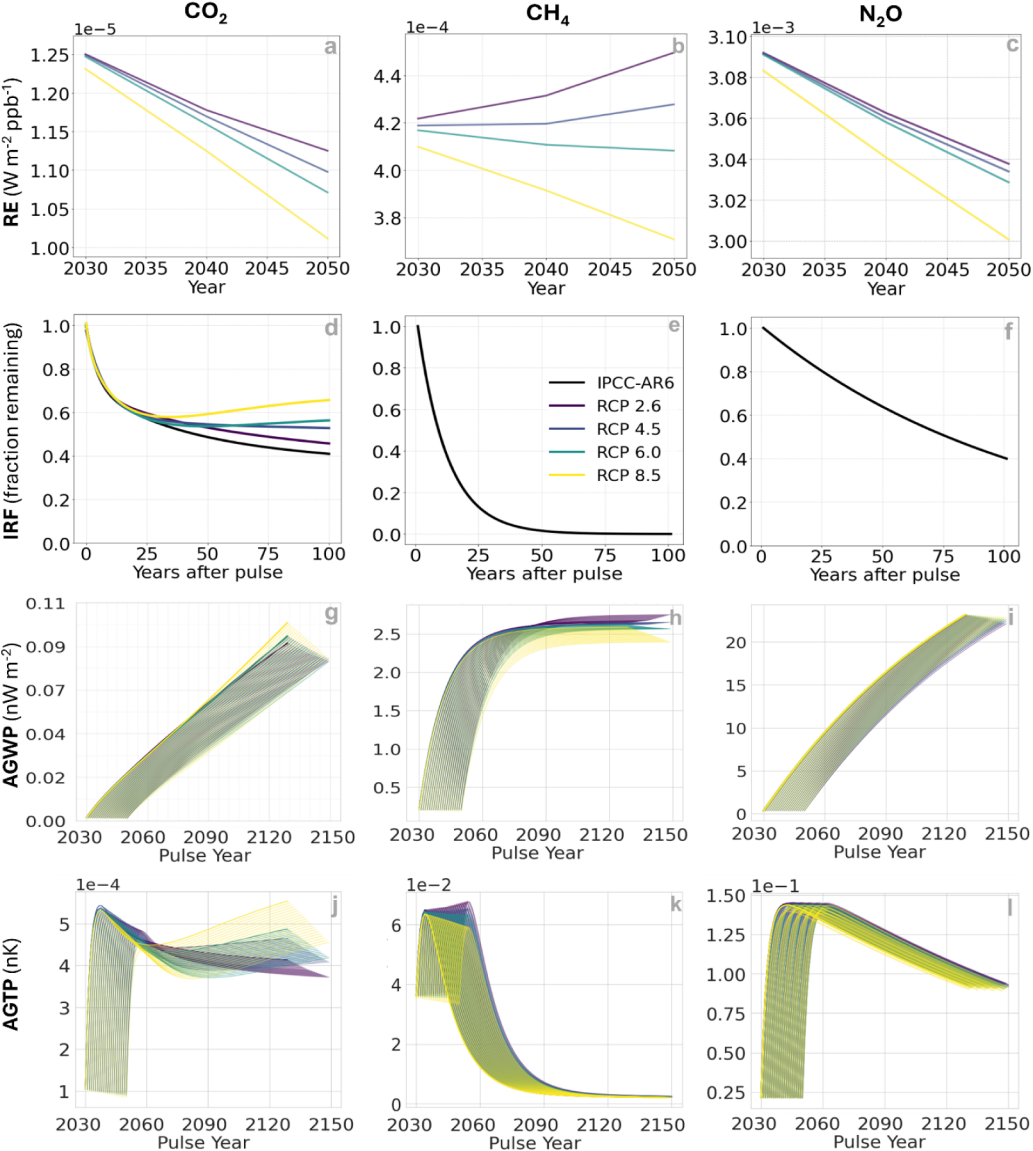
Prospective radiative efficiencies (RE), impulse response functions (IRFs), absolute global warming potentials (AGWP), and absolute global temperature change potentials (AGTP) under different RCP scenarios for the REMIND-MAGPIE-SSP5 scenario.^32^ Panels (a–c) show REs for a one ton pulse of CO_2_, CH_4_, and N_2_O under RCPs 2.6, 4.5, 6.0, and 8.5 from 2030 to 2050. Panels (d–f) show the IRFs for CO_2_, CH_4_, and N_2_O under varying concentration pathways. Panels (g–i) present AGWPs and panels (j–l) show AGTPs for pulse emissions of CO_2_, CH_4_, and N_2_O at different future years (2030–2050) and for the investigated RCP scenarios.

The RE of CO_2_ steadily declines under the least stringent climate scenario (RCP 8.5), reaching approximately 1.01 × 10^–5^ W·m^–2^·ppb^–1^ by 2050 (Figure 1a). This represents a 25% reduction compared with the IPCC-AR6 reference value of 1.33 × 10^–5^ W·m^–2^·ppb^–1^. Generally, as the atmospheric concentration of a gas increases, its RE decreases. This trend reflects the logarithmic relationship between CO_2_ concentration and its absorption of infrared radiation (eq 3), which diminishes the marginal impact of additional CO_2_ on radiative forcing.^24^ Under RCP 2.6, CO_2_ RE remains relatively higher than in other scenarios but still falls to 1.13 × 10^–5^ W·m^–2^·ppb^–1^ by 2050, which is 15% lower than the current reference value. A similar declining trend is observed for N_2_O (Figure 1c). Under RCP 8.5, the RE of N_2_O decreases more sharply, reaching 3.00 × 10^–3^ W·m^–2^·ppb^–1^ by 2050 (6% below the IPCC AR6 reference of 3.20 × 10^–3^ W·m^–2^·ppb^–1^). For other RCPs (2.6, 4.5, and 6.0), the RE values remain relatively similar until 2050, ranging between 3.03 and 3.04 × 10^–3^ W·m^–2^·ppb^–1^. CH_4_, which has a shorter atmospheric lifetime, exhibits a different pattern (Figure 1b). RE values under RCPs 2.6 and 4.5 tend to increase up to 2050, reflecting a faster rise in RF relative to the increase in CH_4_ concentrations. In contrast, RE remains nearly constant under RCP 6.0, while under RCP 8.5 it declines to around 3.75 × 10^–4^ W·m^–2^·ppb^–1^, a 4% decrease from the IPCC AR6 reference value of 3.89 × 10^–4^ W·m^–2^·ppb^–1^. These dynamics reflect the varying CH_4_ atmospheric concentration under these scenarios (lower for RCPs 2.6 and 4.5, and higher for RCPs 6.0 and 8.5).

The IRF of CO_2_ is sensitive to the background conditions of the gas (Figure 1d). In general, a pulse emission of CO_2_ emitted to the atmosphere is never fully reabsorbed by the climate system within millennial time scales, with the fraction that remains in the air depending on the saturation of the carbon sinks.^44^ This long-term legacy of CO_2_ atmospheric perturbation underpins the irreversibility of CO_2_-induced global warming and the need to bring net anthropogenic emissions down to zero to stabilize temperature changes.^45–47^ The fraction of CO_2_ remaining in the air 100 years after a pulse emission increases from 41% (present-day IRF) to 46%, 53%, 56%, and 66% for RCPs 2.6, 4.5, 6.0, and 8.5, respectively. In all cases, the IRFs follow a similar decay for about 20 years, but thereafter their patterns start to diverge. The decay continues for RCP 2.6, as this is the scenario with the lowest addition of carbon to the atmosphere and in which the carbon removal rates of the natural carbon sinks (oceans and terrestrial vegetation) are more efficient. By increasing CO_2_ concentrations, the carbon sinks become increasingly saturated and less efficient at removing CO_2_ from the atmosphere.^48,49^ In RCP 4.5, the fraction of CO_2_ remaining in the air tends to stabilize after about 40 years. In the cases of RCPs 6.0 and 8.5, the CO_2_ fraction declines for the first 30 years, but then there is a sharper increase in the fraction remaining in the air until year 100. This is due to the relatively high CO_2_ emission levels in these scenarios that lead to an excess of atmospheric CO_2_ that is beyond the absorption capacity of the natural sinks, which become saturated. More technical descriptions about the behavior of the global carbon cycle at varying atmospheric CO_2_ concentrations are available elsewhere.^49–51^ As discussed in the methods, the IRF for CH_4_ (Figure 1e) and N_2_O (Figure 1f) is simpler and follows a single exponential decay model that is rather insensitive to their background atmospheric concentrations.

By combining IRFs with REs, the AGWPs associated with the emission of 1 ton of CO_2_ for multiple pulse years under various RCP scenarios can be estimated (Figure 1g). Across all RCPs, AGWP values tend to decrease for later pulse years, primarily due to a consistent decline in RE values over the 100 year evaluation period. In RCPs 8.5 and 6.0, this downward trend in REs offsets the slight increase in IRFs observed around 40 years after the emission pulse, yet AGWP values remain relatively higher compared to those of other scenarios. When benchmarked against the IPCC AR6 reference estimate (where the 100-year AGWP for CO_2_ is projected to reach 0.090 nW·m^–2^ per ton of CO_2_), only the 2030 pulse emissions under RCPs 6.0 and 8.5 exceed this threshold. By contrast, for 2050 pulse emissions, AGWP values converge to approximately 0.084 nW·m^–2^ across all RCPs, which is about 7% lower than the IPCC AR6 reference. This reduction reflects the reduced marginal impact of a CO_2_ emission in a future atmosphere with elevated background CO_2_ concentrations.

For CH_4_ AGWPs (Figure 1h), RCP scenarios that result in higher radiative forcing by 2100 are associated with lower REs, leading to a reduction in AGWP values for later pulse years. This downward trend is reversed in RCPs 4.5 and 2.6, where AGWPs increase slightly over time. The variability in CH_4_ AGWPs by 2050 is notably greater than that observed for CO_2_, highlighting the larger sensitivity of CH_4_’s RE values to different RCP scenarios. In contrast, N_2_O (Figure 1i) exhibits the lowest AGWP variability, as the relative differences in RE values across RCPs are much smaller than those for CO_2_ and CH_4_. Interestingly, N_2_O AGWPs are slightly higher under RCP 8.5 compared to RCP 2.6, a pattern opposite to that of CH_4_. Although N_2_O REs decrease from RCP 2.6 to 8.5, this effect can be offset by the reduction in CH_4_ REs under RCP 8.5, which leads to less spectral overlap and reduced competition for infrared absorption. This interaction may enhance the effective radiative impact of N_2_O in high-CH_4_-concentration scenarios.

When analyzing the AGTPs, the response curves for the CO_2_ peak within approximately a decade following the emission pulse and then gradually decline under RCPs 2.6, 4.5, and 6.0 (Figure 1j). In contrast, for the earliest pulse years (2030–2035) under RCP 8.5, AGTP values continue to increase beyond the initial peak, primarily driven by a more rapid rise in AGWPs associated with those pulse years—as previously shown in Figure 1g. While the greater variability in CO_2_ AGTPs largely reflects the diversity in RE trajectories across scenarios, the AGTP curves for later pulse years begin to closely mirror the trends of the IRF curves shown in Figure 1d. As with AGWPs, later pulse years consistently correspond to lower AGTP values across all RCPs. This again illustrates the declining marginal warming impact of a CO_2_ emission in a future atmosphere with elevated background concentrations. AGTPs of RCP 2.6 can approach a value close to that based on current IPCC parameters (which stabilize at around 4 nK per ton of CO_2_ after 100 years) for the earliest pulse emissions. However, for later pulse years under RCP 2.6, AGTP values decline to approximately 3.8 nK after 100 years, which is about 5% lower than the present-day reference values. Conversely, under RCP 8.5, AGTPs can reach 4.5 nK, or 12% above the reference level, highlighting the amplified warming potential of CO_2_ in a high-emissions trajectory due to a higher fraction of CO_2_ remaining airborne at more saturated ocean and terrestrial carbon sinks.

The CH_4_ AGTPs exhibit a different pattern than that of CO_2_ and N_2_O (Figure 1k). Although the temperature response peaks around 5–6 years after the emission pulse, all trajectories converge to the same value of approximately 2 × 10^–4^ nK per ton of CH_4_ by the end of the 100-year period. This sharp decline reflects methane’s short atmospheric lifetime and the rapid adjustment of radiative forcing as CH_4_ concentrations decay over time. Unlike CO_2_ and N_2_O, CH_4_ is classified as a short-lived climate pollutant, with warming effects that are largely reversible within decades after a single emission pulse.^52,53^ In the first three decades following a pulse, CH_4_ AGTPs behave differently depending on the scenario: AGTP values increase for later pulse years under RCP 2.6, while they decrease under RCP 8.5. Nonetheless, by year 100, AGTPs across all RCPs converge to a value slightly below the one derived from present-day IPCC parameters (∼4 × 10^–4^ nK after 100 years), further highlighting the short-term nature of methane’s warming influence.

For N_2_O (Figure 1l), the AGTP values show minimal variability across RCPs and pulse years. Although RCP 2.6 yields slightly higher AGTPs, the values for all scenarios practically overlap within a narrow range of 0.091–0.095 nK after the 100-year period. These are slightly higher than the estimate derived using current IPCC parameters (∼0.090 nK) and indicate relatively stable long-term warming impacts of N_2_O under future emission scenarios.

### 3.2 Prospective Characterization Factors

Figure 2 shows the projected pGWP_100_ and pGTP_100_ values, derived from evolving background concentrations of GHGs under various IAMs, for selected pulse years from 2030 to 2050 at five-year intervals.

**Figure 2:**
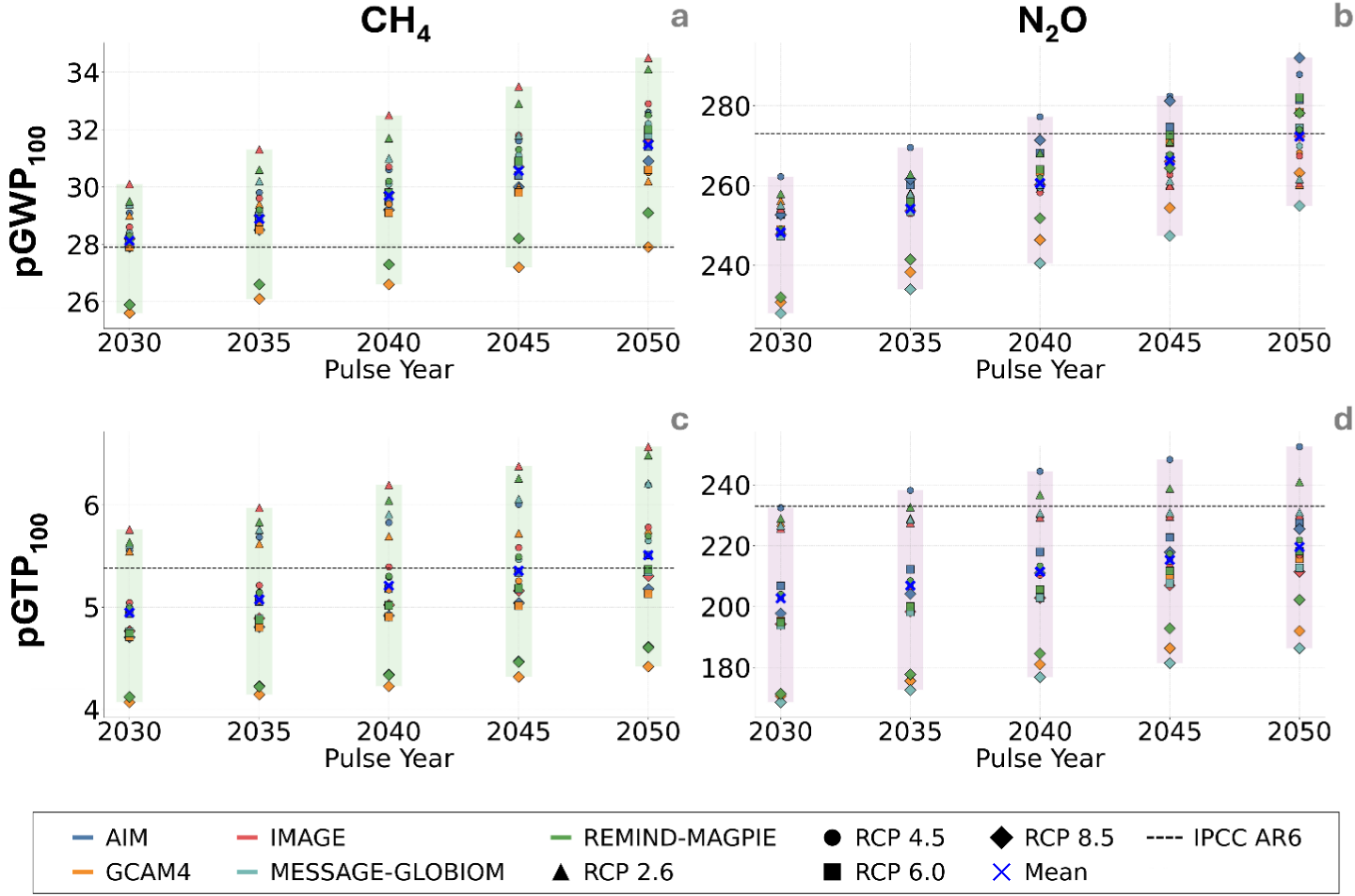
Prospective global warming potential over 100 years (pGWP_100_) and global temperature change potential 100 years (pGTP_100_) after a pulse emission at year 2030–2050 for methane (CH_4_) and nitrous oxide (N_2_O). Results are based on outputs from multiple Integrated Assessment Models (IAMs) for different RCPs; the green and purple shaded bars illustrate the possible span of the CH_4_ and N_2_O metric values, respectively, for each pulse year. Mean values are calculated based on the results obtained from the 18 different IAM-RCP combinations.

For CH_4_ (Figure 2a), the average pGWP_100_ increases from 28.1 ± 1.2 in 2030 to 31.4 ± 1.7 in 2050 (mean ± one standard deviation across all future IAM-RCP scenario combinations). Among individual estimates, the lowest pGWP_100_ value is 25.6, or 8% below the present-day CF, which occurs for a 2030 pulse under the GCAM4-SSP4 RCP 8.5 scenario. This lower value is primarily driven by the higher CO_2_ AGWPs in this scenario, which increase the denominator in the GWP equation and thereby reduce methane’s relative warming impact. In contrast, the highest pGWP_100_ value is 34.5, or 23% above the present-day value, and it occurs for a 2050 pulse in the IMAGE-SSP1 RCP 2.6 scenario. In this case, the CH_4_ AGWPs peak and the CO_2_ AGWPs peak are at their lowest, amplifying the ratio. Overall, CF values tend to rise with later pulse years with most projections exceeding the present-day CFs described in IPCC-AR6.^26^ In general, values are higher at lower RCPs. The findings are consistent with Reisinger et al. (2011),^24^ where the lowest projected GWP_100_ values for CH_4_ are reported for RCP 8.5, largely due to the rapid near-term increase in CH_4_ concentrations that reduces RE and the methane’s marginal climate impact over a 100-year time frame.

For N_2_O pGWP_100_, the mean values increase from 248.2 ± 9.2 in 2030 to 272.0 ± 10.1 in 2050. As shown in Figure 2b, all mean values remain slightly below the present-day GWP_100_ value of 273, with only a few exceptions. The lowest projected pGWP_100_ value, 228, is associated with a 2030 pulse under the MESSAGE-GLOBIOM-SSP2 RCP 8.5 scenario. This low value results from the relatively higher CO_2_ AGWP, which increases the denominator in the GWP equation, thereby lowering the overall pGWP_100_ for N_2_O. Unlike CH_4_ and CO_2_, no consistent trend with the RCP level is observed for N_2_O pGWP_100_. Notably, the maximum value of 292, which is about 7% above the present-day GWP_100_, is found under RCP 8.5, namely, the AIM/CGE-SSP3 scenario for a pulse year in 2050. Reisinger et al. (2011)^24^ also identified RCP 8.5 in 2050 as yielding the highest pGWP_100_ values for N_2_O, while values across RCPs were relatively similar in 2030. Our findings, however, highlight a stronger influence of the underlying IAM framework on projected GWP values, rather than RCP trajectory alone. In general, the highest pGWP_100_ values for N_2_O are driven by the AIM/CGE-SSP3 scenario, while the lowest originate from MESSAGE-GLOBIOM-SSP2.

The pGTP_100_ for CH_4_ increases from 4.92 ± 0.52 in 2030 to 5.47 ± 0.62 in 2050 (Figure 2c), exhibiting a trend similar to that of pGWP_100_, with values rising over time. The minimum and maximum projected values are 4.1 and 6.6, respectively, and represent changes of −24% and +22% relative to the present-day GTP_100_ value of 5.38. Across various RCP and IAM scenarios, this pattern mirrors that of pGWP_100_, with lower values associated with RCP 8.5 and higher values linked to RCP 2.6. For the pGTP_100_ of N_2_O (Figure 2d), values increase from 201.7 ± 19.9 in 2030 to 218.9 ± 15.9 in 2050, with the lowest values occurring under RCP 8.5. The pGTP_100_ values for N_2_O are generally lower than the present-day N_2_O GTP_100_ of 233, a behavior largely driven by the increased AGTP of CO_2_, which exceeds current levels, particularly in scenarios with earlier emission pulses. In contrast to CH_4_, the REs of N_2_O do not increase over time—and consequently AGTP—consistently decline across all RCP scenarios. The lowest value, 168.6, represents a 27% decrease relative to current GTP_100_. Exceptions to this trend occur in both the AIM-CGE-SSP3 and REMIND-MAGPIE-SSP5, where the maximum value of 252 is observed under RCP 4.5 for a 2050 pulse year, which is an increase of approximately 8% relative to the present-day value.

### 3.3 Impact of Prospective Characterization Factors on LCA Results

Figure 3 presents the sensitivity to pGWP_100_ and pGTP_100_ of the life-cycle climate change impacts of two production activities, namely, nonbasmati rice and nitric acid. For this example, a prospective LCA fixed at the year 2040 without updates to the background database is considered. Climate change impacts for rice characterized with pGWP_100_ range from −1.7% to +7.5% relative to the results calculated with present-day CFs (blue bars in Figure 3a). The largest deviation occurs under the IMAGE-SSP1 RCP 2.6 scenario, which yields the most extreme CFs. Since CH_4_ and N_2_O contribute 38% and 9% of rice life-cycle impacts, respectively, pGWP_100_ values can reach up to 1.62 kg of CO_2_-eq per kg of rice, compared to about 1.5 kg of CO_2_-eq with current CFs. For nitric acid (Figure 3b), impacts are lower and less variable, with deviations from −2.8% to +0.5% relative to the baseline of 0.94 kg of CO_2_-eq. This is largely due to the higher share of N_2_O (18%) compared to that of CH_4_ (7%) in its impact profile. Since N_2_O CFs under pGWP_100_ can be lower than present-day values, they partially offset CH_4_ increases, resulting in net changes typically between −1% and +0.5%.

**Figure 3:**
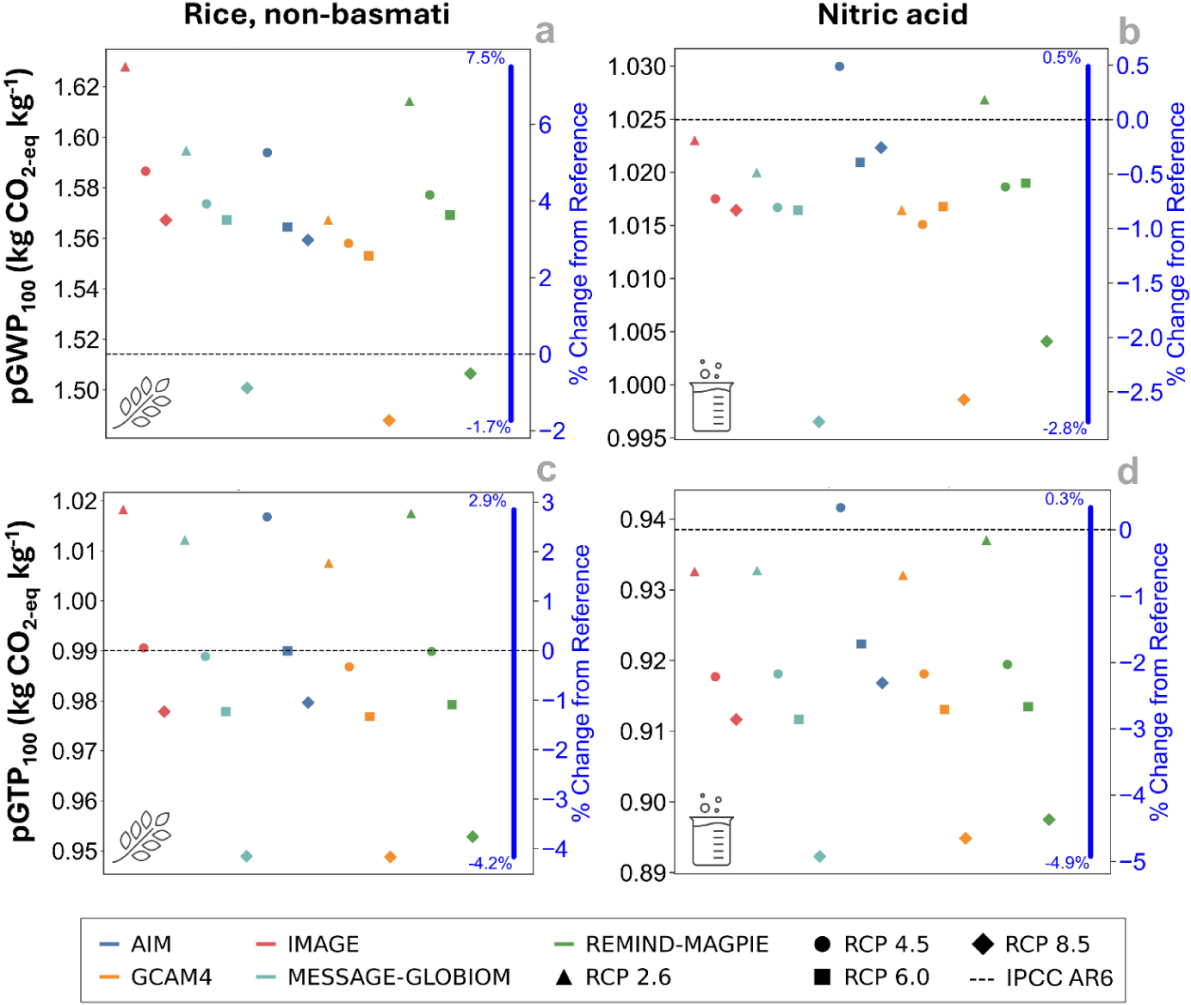
Sensitivity analysis of climate impacts in 2040 for nonbasmati rice production and nitric acid production in 50% aqueous solution. Panels (a,b) illustrate the sensitivity to pGWP_100_ and panels (c,d) pGTP_100_. Blue bars represent percentage changes relative to the present-day characterization factors from IPCC-AR6 (indicated by dashed lines).

Under pGTP_100_ (Figure 3c,d), the largest reductions for both rice and nitric acid occur under the GCAM-SSP4 and MESSAGE-GLOBIOM SSP2 models with RCP 8.5, which yield the lowest combined CFs for CH_4_ and N_2_O. Nitric acid shows slightly more pronounced negative deviations, with the lowest reaching −4.9% due to its strong dependence on N_2_O emissions. For rice production, a slightly broader range of changes from the reference (blue bars in Figure 3) is observed compared to nitric acid due to the greater contribution from CH_4_.

Figure 4 shows the sensitivity to pCFs of a group of activities, including power generation, transport, food, materials, and chemicals production. The most affected by the changes in CFs are rice and cheese production, which show the highest sensitivity to both metrics. For pGWP_100_ (Figure 4a), cheese can achieve life cycle impacts up to 8% higher than the reference, largely driven by CH_4_ (27%) and N_2_O (13%) impacts originating from ruminant digestion and the production of feed used in cow milk production. Within the food sector, trout farming in semi-intensive systems ranks fifth, as it is particularly impacted by upstream N_2_O emissions (which represent 6% of total life cycle impacts) linked to soybean cultivation for trout feed. The chemical sector, represented by ammonium nitrate and nitric acid production, is among the activities with the largest sensitivities represented in Figure 4a. The nitrogen-based fertilizer has the highest contribution of N_2_O to total life cycle impacts (35%) among all activities investigated in this study. Such a value is reflected in the lowest change relative to the reference to pGWP_100_ (−3.6%), as the prospective N_2_O CFs used to calculate pGWP_100_ can fall below the present-day CF. As CH_4_ emissions are practically zero in this activity, the sensitivity profile of ammonium nitrate is dominated by the uncertainty of CFs associated with N_2_O. For other activities such as transport, power generation, and cement production, the sensitivity to pGWP_100_ is much lower because CO_2_ impacts are predominant.

**Figure 4:**
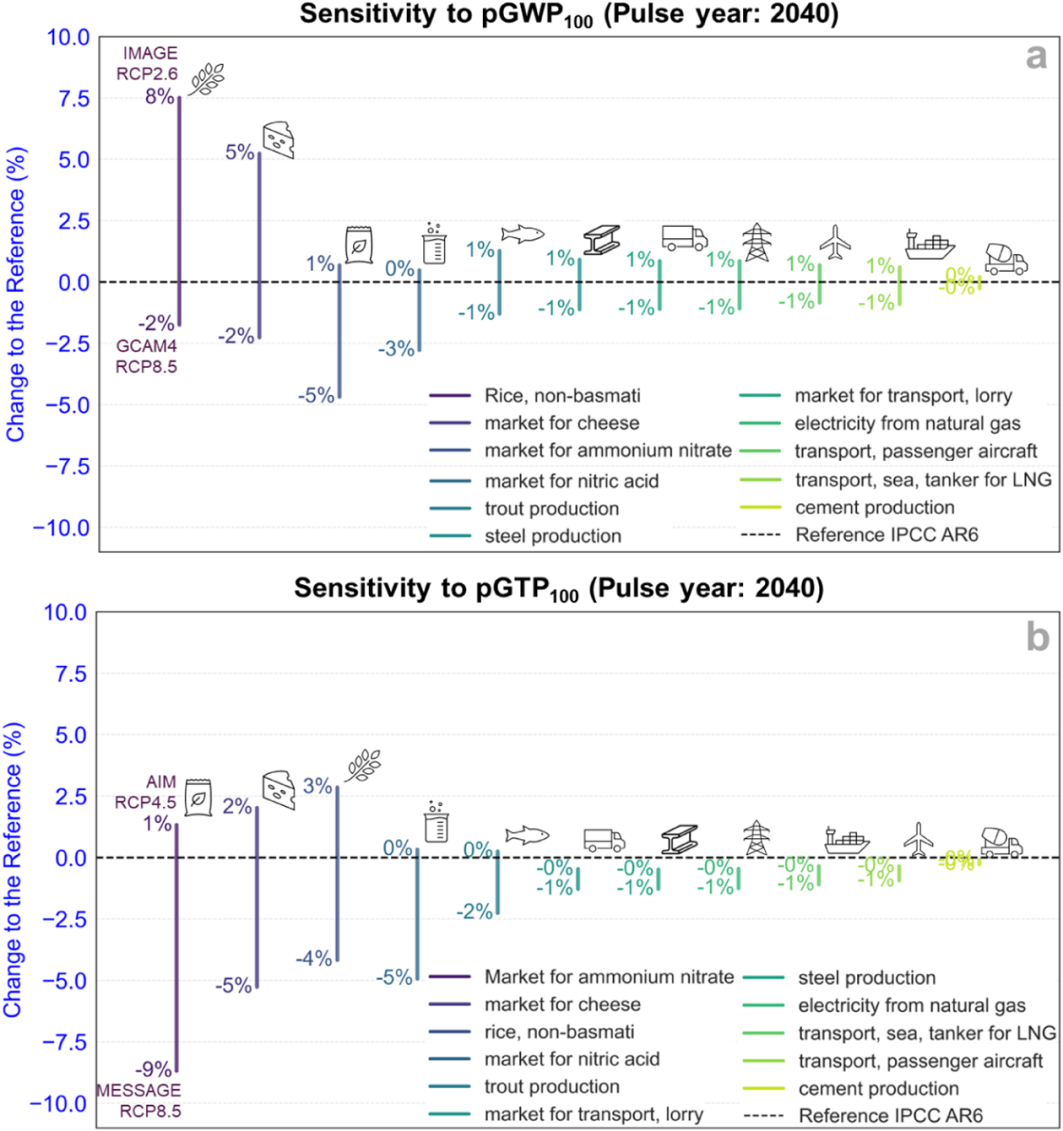
Ranking sensitivity to pCFs for the life-cycle climate change impacts of the selected ecoinvent 3.9 activities in the year 2040. Panel (a) illustrates the sensitivity of activities to global warming potential over 100 years (pGWP_100_) while panel (b) shows the sensitivity to global temperature change potential 100 years after emission (pGTP_100_). Colored bars represent the percentage change relative to the present-day characterization factors from the IPCC Sixth Assessment Report (AR6),^26^ indicated by dashed lines. Activities are arranged from left to right in order of decreasing sensitivity.

With pGTP_100_ (Figure 4b), ammonium nitrate shows the largest potential decrease (−9% relative to the present-day value). This is primarily due to the dominant role of N_2_O in its life cycle impacts combined with CFs that are lower than the reference. Rice production, which ranked highest in sensitivity under pGWP_100_, decreases its sensitivity under pGTP_100_. This shift reflects the higher CH_4_-to-N_2_O ratio in its life cycle emissions. Since CH_4_ is a short-lived greenhouse gas, its relative importance decreases when using a metric like GTP, which captures the instantaneous impact at a specific time horizon rather than integrating effects over time as GWP does. In contrast, activities dominated by fossil CO_2_ emissions—such as transport, power generation, steel, and cement production—exhibit sensitivity even lower under pGTP_100_ than under pGWP_100_. This aligns with their emission profiles, which are largely unaffected by non-CO_2_ GHGs. As discussed in detail elsewhere,^38^ the differences between Figure 4a and b highlight how the choice of the climate metric can affect the estimated climate impact of a given product, especially when there is a high share of non-CO_2_ GHG emissions.

### 3.4 Combination of Prospective Inventories and Prospective CFs

Figure 5 shows the sensitivity of the estimated climate change impacts to changes in the background database by adapting ecoinvent v.3.9 to the prospective REMIND-SSP5 scenarios RCP 2.6 and RCP 8.5 for the year 2040, in comparison to the cases where the background database is not transformed and only the CFs are prospective. These REMIND-RCP scenarios were chosen because they are the only ones currently available in the Premise tool^21^ that fully align with the IIASA marker scenarios, and they represent the two extremes of the RCP range examined in this study. When only the pCFs are modified, represented by the blue bars, there is a general trend of net increases in climate impacts assessed with GWP_100_ for CH_4_-emission dominated activities and net decreases when GTP_100_ is used for activities dominated by N_2_O emissions compared to the reference case (i.e., no prospective inventories nor pCFs). However, adding changes in the background database significantly alters the outcomes, as indicated by the colored bars in Figure 5.

**Figure 5:**
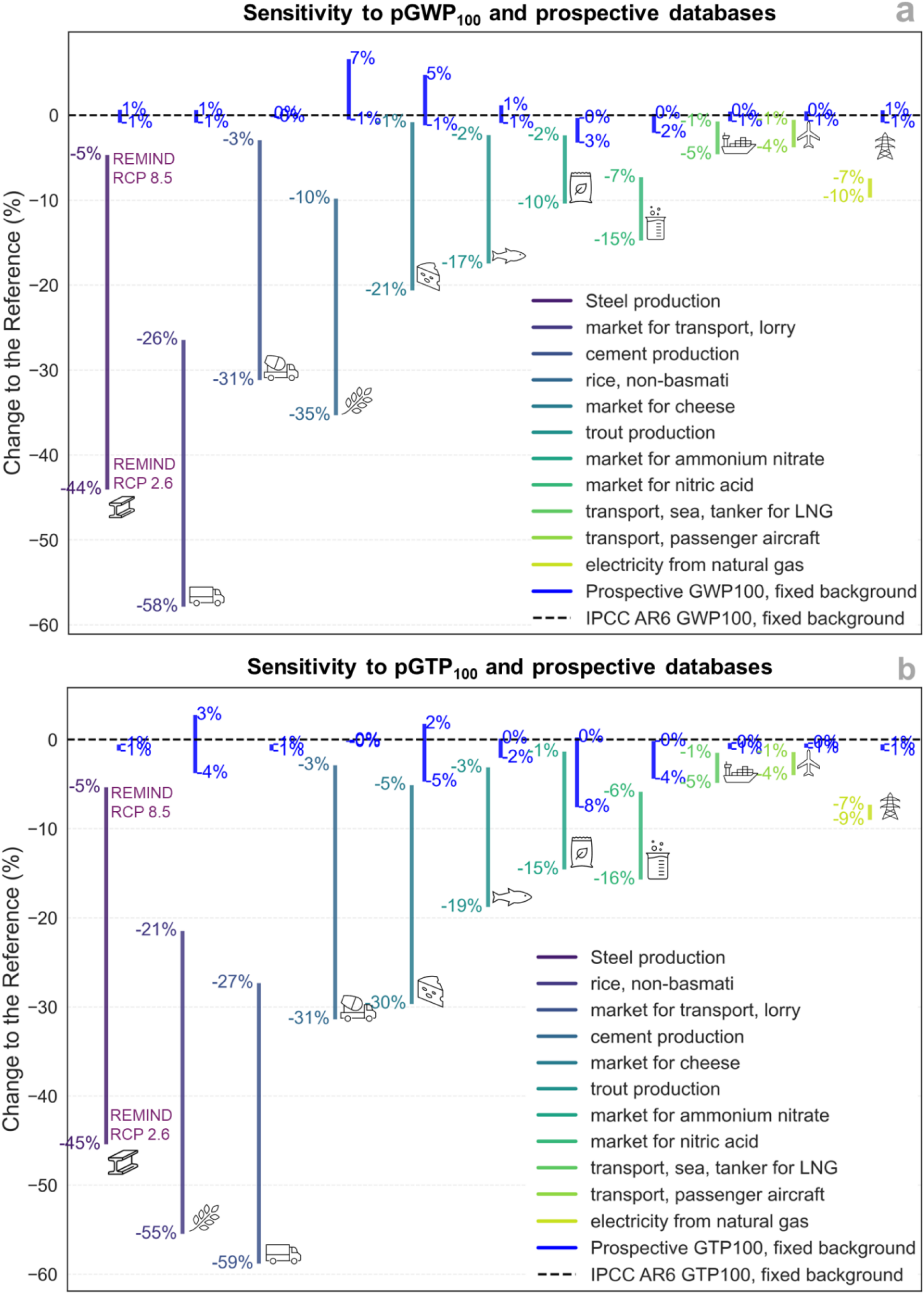
Sensitivity of activities to prospective climate change characterization factors (CFs) and prospective ecoinvent 3.9 data under REMIND-SSP2-2.6 and REMIND-SSP5-8.5 scenarios for the year 2040. Panel (a) illustrates the sensitivity of activities to global warming potential over 100 years (pGWP_100_) while panel (b) shows the sensitivity to global temperature change potential 100 years after emission (pGTP_100_). Activities are ordered from left to right by decreasing the sensitivity. The bars indicate the percentage deviation from the reference case, which is based on the fixed ecoinvent 3.9 database^43^ and present-day CFs^26^ (represented by the dashed line). Blue bars isolate the effect of changing only the CFs, i.e., keeping the present-day background database.

Steel production emerges as the most affected activity by changes in the background scenario under pGWP_100_ (Figure 5a), representing a significant shift in sensitivity compared to the fixed background analysis (Figure 4a) where it ranked sixth. In steel production, net reductions in impacts range from −5% to −44%. These reductions are driven by progressive improvements in energy efficiency and declining direct emissions from both steel and pig iron production.^21^ The maximum reduction corresponds to the RCP 2.6 scenario, which aligns with the adoption of technologies such as carbon capture and storage (CCS). Similarly, road transport is significantly influenced by changes in the prospective databases, reflecting shifts in fleet composition such as the introduction of electric trucks and enhanced energy efficiency as well as a growing share of renewable fuels in internal combustion engines by 2040. Relative to the fixed background analysis, road transport achieves the largest reduction in impacts across all assessed activities, reaching −58% under the RCP 2.6 scenario. Other CO_2_-intensive activities, such as cement production, also become increasingly responsive to changes in process inventories, showing consistent reductions in pGWP_100_ across all RCP scenarios. For Portland cement, projected decreases in fuel consumption (e.g., coke) and associated CO_2_ emissions reflect ongoing improvements in the foreground system, particularly in clinker production. In the RCP 2.6 scenario, the adoption of CCS further amplifies these reductions, potentially lowering the impacts by up to 31%. By contrast, maritime transport and aviation exhibit lower sensitivity to changes in background scenarios due to the limited representation of future developments in fleet composition and fuel mix for ships and aircraft in current prospective scenario modeling.

In terms of pGTP_100_ (Figure 5b), steel production also experiences the highest sensitivity, primarily due to the reduction in fossil-CO_2_ emissions driven by cleaner production technologies, with the variability of the impacts ranging from −5% to −45%. When compared with the use of pGWP_100_, rice production is the activity that shows the most pronounced change in sensitivity when shifting to the pGTP_100_. The relative contributions of CH_4_ and N_2_O to the total direct emissions (from rice cultivation and fertilizer use) increase alongside the significant reduction of fossil CO_2_ from background activities (such as fuel mixes, road transport, and fertilizer production). As a result, the variability associated with CFs becomes even more impactful under REMIND-SSP5 RCP 2.6, which represents the scenario with the greatest level of decarbonization in the supply chain.

We further exemplify how pCFs can influence LCA outcomes for similar products but produced from different value chains, a perspective that is relevant when LCA is used as a decision support tool by assessing rice production in 2040 in the United States and India using pGTP_100_ under the REMIND-SSP5-RCP 8.5 scenario. With conventional pCFs, the U.S. rice production has higher impacts (758 vs 750 kg of CO_2_-eq/ton). When applying prospective CFs, the results reverse, and rice production in India exhibits higher impacts (704 vs 714 kg CO_2_-eq/ton). A similar trend is found for “market for nitric acid, without water” when comparing Rest of the World (RoW) and UN-Oceania. Without pCFs, UN-Oceania shows higher impacts (1,840 vs 1,709 kg CO_2_-eq/ton for RoW), but applying pCFs reverses the results (1,604 vs 1,595 kg CO_2_-eq/ton). This occurs because the use of pGTP_100_ makes results particularly sensitive when relatively high emissions of N_2_O are present. The results of both examples can be found in Figures S31 and S32 in the SI.

## 4 Discussion

Comparing the pCFs estimated in this study with those available in the literature is challenging, as no previous publication has projected pCFs across multiple climate metrics using the IAM-SSP marker scenarios. Our results for CH_4_ pGWP_100_ for the period 2030–2050 range from 25.6 to 34.5, and slightly differ from the projections by Lan and Yao (2022),^29^ who reported values between 29 and 41. For N_2_O, their estimated range of 280–380 is broader than our findings, which fall between 232 and 292. Although their method accounts for changes in REs, they do not clearly specify the IAM model or SSP scenario used in their analysis, limiting the comparability of their results. Moreover, unlike our approach, their study does not incorporate variations in the CO_2_ IRFs across RCPs, a factor that likely contributes to the observed differences. To our knowledge, no studies have examined other climate metrics (e.g., GTP_100_) in this context under such conditions.

Potential future changes in GWPs were originally modeled by Reisinger et al. (2011),^24^ who incorporated both changes in REs and climate–carbon cycle feedback under different RCPs. Although their study provided projections for the GWP of N_2_O and CH_4_, a direct comparison with our quantitative results is not feasible, as their analysis did not link their data directly to specific IAM outputs. Nevertheless, some trends in their findings align with ours. For instance, our results for methane’s projected GWP_100_ also lead to the lowest GWP_100_ values under the RCP 8.5 scenario. This outcome is primarily attributed to the rapid increase in CH_4_ concentrations in the near term, which reduces its RE and, consequently, its marginal climate impact over a 100-year horizon. Their analysis of the 2030–2050 period further identified the highest GWP_100_ values under RCP 2.6, which is in line with our finding.

A key limitation in our analysis is the absence of a multimodel mean for the IRFs of CO_2_ across different RCP scenarios, which led us to adopt an indirect approach to address this gap. It is also important to note that the IRF used by the IPCC for estimating metric values in its last assessment report (dated 2019)^26^ is the one from Joos et al., which is based on a background atmospheric concentration of CO_2_ of 389 ppm, which refers to the year 2010.^22^ This is lower than the current concentration of 427 ppm. As such, there is an inconsistency as the IRF available for CO_2_ becomes progressively outdated and does not reflect present-day conditions nor align with updated RE values. Addressing this issue requires simulations from complex climate models to replicate experiments such as those in Joos et al., requiring competencies and tools that go beyond the current capabilities of the LCA community. Given the importance of IPCC climate metrics for LCA, establishing a collaborative dialog with climate scientists can stimulate the development of an updated set of IRFs that reflect current climate conditions and span multiple future RCPs. Although future changes in the IRFs for CH_4_ and N_2_O are expected to only have a minor influence on our results given the relative stability of their lifetimes across the historical IPCC assessment reports, the future availability of more refined and time-dependent IRFs could improve the accuracy of the estimated pCFs.

The radiative forcings and GHG concentrations from the IAM-SSP-RCP marker scenarios used in this study are based on the best available data from the IIASA Legacy Database.^32^ Incorporating other or more recent IAM results could potentially enrich the pCFs and affect the mean statistics elaborated in this paper.

The coupling between pCFs and prospective background databases is hindered by the limited number of IAM scenarios that are currently available within existing prospective tools. In the current version of Premise (v. 2.2.7), only the REMIND-MAgPIE-SSP5 scenario is available in more than one RCP. Extending the availability of prospective databases to match other IAM marker scenarios can be beneficial for prospective LCA studies aiming at combining changing CFs with changes in background databases. As the Premise framework is open to add more IAM scenarios, this gap can be covered by future studies such as the one that coupled the TIAM-UCL model with Premise and ecoinvent.^54^

This study focuses on the three primary GHGs (CO_2_, CH_4_, and N_2_O) with the goal of introducing and demonstrating a consistent methodological approach for estimating pCFs. These gases are a logical starting point to showcase the method to compute pCFs, and they dominate most of the LCA applications. The same approach can be reiterated to estimate pCFs of other GHGs, including chlorinated and fluorinated compounds, for which data are available. Expanding the method to include short-lived climate forcers such as carbon monoxide (CO), nitrogen oxides (NO_*x*_), and sulfur oxides (SO_*x*_) will require additional consideration due to their complex atmospheric behavior and indirect effects. Future research should aim to adapt and refine the framework to accommodate these substances, supported by more comprehensive data sets and modeling tools.

The pCFs computed in this study are timely given the increasing number of pLCAs conducted in recent years.^2^ Unlike previous studies, we provide analytically derived and updatable CFs tailored to LCA that include multiple metrics and align with IPCC methods and IAM scenarios used in pLCA. These features ensure consistency with frameworks for modifying background databases^21^ and enable straightforward integration into existing pLCA tools using tabulated values (Tables S.2–S.9 of the SI). The provided REs, IRFs, and Python codes for AGWP, AGTP, pGWP_100_, pGWP_20_, pGTP_50_, and pGTP_100_ enable extended calculations of pCFs of up to 2100. Emission inventories can be adapted to the year at which the emission occurs, a procedure that can be facilitated by integrating these pCFs with dynamic LCA tools that are becoming increasingly available.^55,56^ For more generic applications or uncertainty considerations aiming to provide insights on how alternative scenarios influence LCA results, mean CFs per RCP with the associated standard deviations can be used. This approach enables the exploration of how a single climate target can be met through different pathways, offering insights into the variability arising from alternative socioeconomic and technological assumptions. In cases in which a specific future climate target is not specified, the average and ranges from all of the available CFs for each given pulse year can be considered. Conversely, averaging CFs across SSPs for different RCPs is likely less meaningful, given the substantial variability in impacts that can occur across SSPs. In all cases, the aggregation method used for pCFs should be consistent with the type of IAM-RCP combination considered for projecting process inventories. This variety of applications can be enhanced by uploading individual and aggregated pCFs (for pGWP_100_, pGWP_20_, pGTP_50_, and pGTP_100_) into existing LCA tools such as Brightway2. Ultimately, we emphasize the need for a harmonized framework that integrates prospective metrics across impact categories beyond climate change, thereby supporting the broader implementation of pLCA.

As presented in the sensitivity analysis, the use of pCFs can result in significant variations in life-cycle climate change impacts across activities, even for identical products sourced from different regions or supply chains. As exemplified by rice and nitric acid production, pCFs can exert a greater influence on decision-making involving value chains in which nitrous oxide emissions play a larger role.

The outcomes of this study are primarily intended for the LCA community, aiming to strengthen the methodological consistency of pLCA and apply the prospective analysis to both the inventory and the LCIA phase. The availability of pGWPs and pGTPs is a step forward in developing dynamic, time-dependent climate impact assessment, where CFs vary with the emission year and evolving background conditions. By integrating pulse-year-specific CFs into temporally explicit modeling frameworks, our approach enhances the accuracy, consistency, and policy relevance of future-oriented climate impact evaluations. The prospective CFs developed here are presented in table format in the SI, together with the code to generate them, and as such, they can be readily implemented in pLCA tools to be consistently coupled with the IAM scenarios used to produce future background databases. Alternatively, CFs averaged by a specific RCP can be used in more streamlined applications to reflect a range of plausible futures or to explore scenario-induced variability in an uncertainty analysis. While this study focuses on CH_4_ and N_2_O, future work can extend this framework to generate pCFs for the remaining GHGs, enabling the creation of complete and harmonized data sets for other climate forcers. This research ultimately supports a robust evolution of pLCA approaches toward greater alignment with forward-looking climate policies and decision-making contexts.

## Supplementary Material

























































## Notes

(XLSX)­GTP100_CH4_table
(XLSX)­GTP100_N2O_table
(XLSX)­GWP100_CH4_table
(XLSX)­GWP100_N2O_table
(XLSX)­IPCC2021 GTP100
(XLSX)­IPCC2021 GTP50
(XLSX)­IPCC2021 GWP100
(XLSX)­IPCC2021 GWP20
(XLSX)­pIRF_CH4
(XLSX)­pIRF_CO2
(XLSX)­pIRF_N2O
(XLSX)­pRE_CH4_AIM
(XLSX)­pRE_CH4_GCAM4
(XLSX)­pRE_CH4_IMAGE
(XLSX)­pRE_CH4_MESSAGE
(XLSX)­pRE_CH4_REMIND
(XLSX)­pRE_CO2_AIM
(XLSX)­pRE_CO2_GCAM4
(XLSX)­pRE_CO2_IMAGE
(XLSX)­pRE_CO2_MESSAGE
(XLSX)­pRE_CO2_REMIND
(XLSX)­pRE_N2O_AIM
(XLSX)­pRE_N2O_GCAM4
(XLSX)­pRE_N2O_IMAGE
(XLSX)­pRE_N2O_MESSAGE
(XLSX)­pRE_N2O_REMIND
(XLSX)­selected_flows
This file provides complementary results, supporting figures, supporting pGWP and pGTP tables, and supporting files and codes (PDF)

## Abbreviations:

AGTP: absolute global temperature change potential
AGWP: absolute global warming potential
AR4: IPCC Fourth Assessment Report
AR5: IPCC Fifth Assessment Report
AR6: IPCC Sixth Assessment Report
CCS: carbon capture and storage
CFs: characterization factors
CH_4_: methane
CO_2_: carbon dioxide
ESMs: Earth system models
GTP: global temperature change potential
GWP: global warming potential
IAMs: integrated assessment models
IPCC: Intergovernmental Panel on Climate Change
IRF: impulse response function
LCI: life cycle inventory
LCIA: life cycle impact assessment
N_2_O: nitrous oxide
ODPs: ozone depletion potentials
pGTP_100_: prospective global temperature change potential over 100 years
pGTP_50_: prospective global temperature change potential over 50 years
pGWP_100_: prospective global warming potential over 100 years
pGWP_20_: prospective global warming potential over 20 years
pLCA: prospective life cycle assessment
PREMISE: PRospective EnvironMental Impact AsSEssment
RCPs: representative concentration pathways
RE: radiative efficiency
SSPs: shared socioeconomic pathways
SI: Supporting Information
TAR: IPCC Third Assessment Report
