# Supplementary material for: Prospective Characterization Factors for Assessing Climate Change Impacts in Life Cycle Assessments
Source: Environ Sci Technol. 2026 Jan 21;60(4):3202–15. doi: 10.1021/acs.est.5c12391 (PMC12874512; doi:10.1021/acs.est.5c12391)
Supplement: Supplementary file 28 [file es5c12391_si_028.pdf]

Supporting Information

Title: Prospective characterization factors for assessing climate change impacts in life cycle assessments

Authors: Marcos D.B. Watanabe, Francesco Cherubini

Summary

Section 1. Input data and complementary results 4

Table S.1. Additional activities from ecoinvent 3.9 used in the sensitivity analysis 4

Figure S.1. Absolute global warming potential (AGWP) of CO_2 under IMAGE- Shared Socioeconomic Pathway 1 (SSP1). 4

Figure S.2. Absolute global warming potential (AGWP) of CO2 under MESSAGE- Shared Socioeconomic Pathway 2 (SSP2) scenarios. 5

Figure S.3. Absolute global warming potential (AGWP) of CO_2 under AIM-CGE- Shared Socioeconomic Pathway 3 (SSP3) scenarios 5

Figure S.4. Absolute global warming potential (AGWP) of CO_2 under GCAM4- Shared Socioeconomic Pathway 4 (SSP4) scenarios. 6

Figure S.5. Absolute global warming potential (AGWP) of CO_2 under REMIND- Shared Socioeconomic Pathway 5 (SSP5) scenarios. 6

Figure S.6. Absolute global warming potential (AGWP) of CH_4 under IMAGE- Shared Socioeconomic Pathway 1 (SSP1) scenarios. 7

Figure S7. Absolute global warming potential (AGWP) of CH_4 under MESSAGE- Shared Socioeconomic Pathway 2 (SSP2) scenarios. 7

Figure S8. Absolute global warming potential (AGWP) of CH_4 under AIM-CGE- Shared Socioeconomic Pathway 3 (SSP3) scenarios. 8

Figure S9. Absolute global warming potential (AGWP) of CH_4 under GCAM4- Shared Socioeconomic Pathway 4 (SSP4) scenarios 8

Figure S10. Absolute global warming potential (AGWP) of CH_4 under REMIND- Shared Socioeconomic Pathway 5 (SSP5) scenarios 9

Figure S11. Absolute global warming potential (AGWP) of N_2O under IMAGE- Shared Socioeconomic Pathway 1 (SSP1) scenarios. 9

Figure S12. Absolute global warming potential (AGWP) of N_2O under MESSAGE- Shared Socioeconomic Pathway 2 (SSP2) scenarios. 10

Figure S13. Absolute global warming potential (AGWP) of N_2O under AIM- Shared Socioeconomic Pathway 3 (SSP3) scenarios. 10

Figure S14. Absolute global warming potential (AGWP) of N_2O under GCAM4- Shared Socioeconomic Pathway 4 (SSP4) scenarios 11

Figure S15. Absolute global warming potentials (AGWP) of N_2O under REMIND- Shared Socioeconomic Pathway 5 (SSP5) scenarios 11

Figure S.16. Absolute global temperature change potential of CO_2 (AGTP) under IMAGE- Shared Socioeconomic Pathway 1 (SSP1) scenarios. 12

Figure S.17. Absolute global temperature change potential (AGTP) of CO_2 under MESSAGE- Shared Socioeconomic Pathway 2 (SSP2) scenarios 12

Figure S.18. Absolute global temperature change potential (AGTP) of CO_2 under AIM-CGE- Shared Socioeconomic Pathway 3 (SSP3) scenarios. 13

Figure S.19. Absolute global temperature change potential (AGTP) of CO_2 under GCAM4- Shared Socioeconomic Pathway 4 (SSP4) scenarios. 13

Figure S.20. Absolute global temperature change potential (AGTP) of CO2 under REMIND- Shared Socioeconomic Pathway 5 (SSP5) scenarios 14

Figure S.21. Absolute global temperature change potential (AGTP) of CH_4 under IMAGE- Shared Socioeconomic Pathway 1 (SSP1) scenarios. 14

Figure S22. Absolute global temperature change potential (AGTP) of CH_4 under MESSAGE- Shared Socioeconomic Pathway 2 (SSP2) scenarios 15

Figure S23. Absolute global temperature change potential (AGTP) of CH_4 under AIM-CGE- Shared Socioeconomic Pathway 3 (SSP3) scenarios 15

Figure S24. Absolute global temperature change potential (AGTP) of CH_4 under GCAM4- Shared Socioeconomic Pathway 4 (SSP4) scenarios. 16

Figure S25. Absolute global temperature change potential (AGTP) of CH_4 under REMIND- Shared Socioeconomic Pathway 5 (SSP5) scenarios. 16

Figure S26. Absolute global temperature change potential (AGTP) of N_2O under IMAGE- Shared Socioeconomic Pathway 1 (SSP1) scenarios. 17

Figure S27. Absolute global temperature change potential (AGTP) of N2O under MESSAGE- Shared Socioeconomic Pathway 2 (SSP2) scenarios 17

Figure S28. Absolute global temperature change potential (AGTP) of N_2O under AIM- Shared Socioeconomic Pathway 3 (SSP3) scenarios. 18

Figure S29. Absolute global temperature change potential (AGTP) of N_2O under GCAM4- Shared Socioeconomic Pathway 4 (SSP4) scenarios 18

Figure S30. Absolute global temperature change potential (AGTP) of N_2O under REMIND- Shared Socioeconomic Pathway 5 (SSP5) scenarios. 19

Figure S31. Comparison of 'rice production, non-basmati' in India and the United States. 19

Figure S32. Comparison of 'market for nitric acid, without water, in 50% solution state' in UN-Oceania and Rest of the World (RoW). 20

Table S.2. Results for CH_4 $pGWP_{100}$ under different IAM-SSP-RCP scenarios. 21

Table S.3. Results for N_2O $pGWP_{100}$ under different IAM-SSP-RCP scenarios. 22

Table S.4. Results for CH_4 $pGWP_{20}$ under different IAM-SSP-RCP scenarios. 23

Table S.5. Results for N_2O $pGWP_{20}$ under different IAM-SSP-RCP scenarios. 24

Table S.6. Results for CH_4 $pGTP_{100}$ under different IAM-SSP-RCP scenarios 25

Table S.7. Results for N_2O $pGTP_{100}$ under different IAM-SSP-RCP scenarios 26

Table S.8. Results for CH_4 $pGTP_{50}$ under different IAM-SSP-RCP scenarios 27

Table S.9. Results for N_2O $pGTP_{50}$ under different IAM-SSP-RCP scenarios 28

Section 2. Python codes for implementing available CFs 29

Code #1. Loading $pGWP_{100}$ characterization factors in Brightway 29

Code #2. Loading $pGTP_{100}$ characterization factors 34

Section 3. GWP & GTP – Unified Folder Structure and Guidelines for using AGWP and AGTP codes 39

Unified Folder Structure 39

Folder Description 40

Combined Workflow Summary (GWP & GTP) 40

Code #3. CO2 AGWP for multiple IAM-SSP-RCP scenarios 42

Code #4 CH_4 AGWP for multiple IAM-SSP-RCP scenarios 45

Code #5 N_2O AGWP for multiple IAM-SSP-RCP scenarios 50

Code #6. Generating GWP20 and GWP100 values for CH_4 and N_2O (Pulse years: 2030-2100)54

Code #7. AGTP of CO_2 for multiple IAM-SSP-RCP scenarios 56

Code #8. AGTP of CH_4 for multiple IAM-SSP-RCP scenarios 60

Code #9. AGTP of N_2O for multiple IAM-SSP-RCP scenarios 64

Code #10. Generating GTP50 and GTP100 values for CH4 and N2O (Pulse years: 2030-2100 68

Section 1. Input data and complementary results

Table S.1. Additional activities from ecoinvent 3.9 used in the sensitivity analysis

Activity	Location
market for cheese from cow milk, fresh, unripened	GLO
trout, production in semi-intensive system, in lake	RoW
market for ammonium nitrate	RoW
electricity production, natural gas, combined cycle power plant	RoW
cement production, Portland	Europe without Switzerland
steel production, converter, unalloyed	RER
market for transport, freight, lorry, unspecified	RER
transport, passenger aircraft, long haul	GLO
transport, freight, sea, tanker for liquefied natural gas	GLO

Figure S.1. Absolute global warming potential (AGWP) of CO_2 under IMAGE- Shared Socioeconomic Pathway 1 (SSP1).

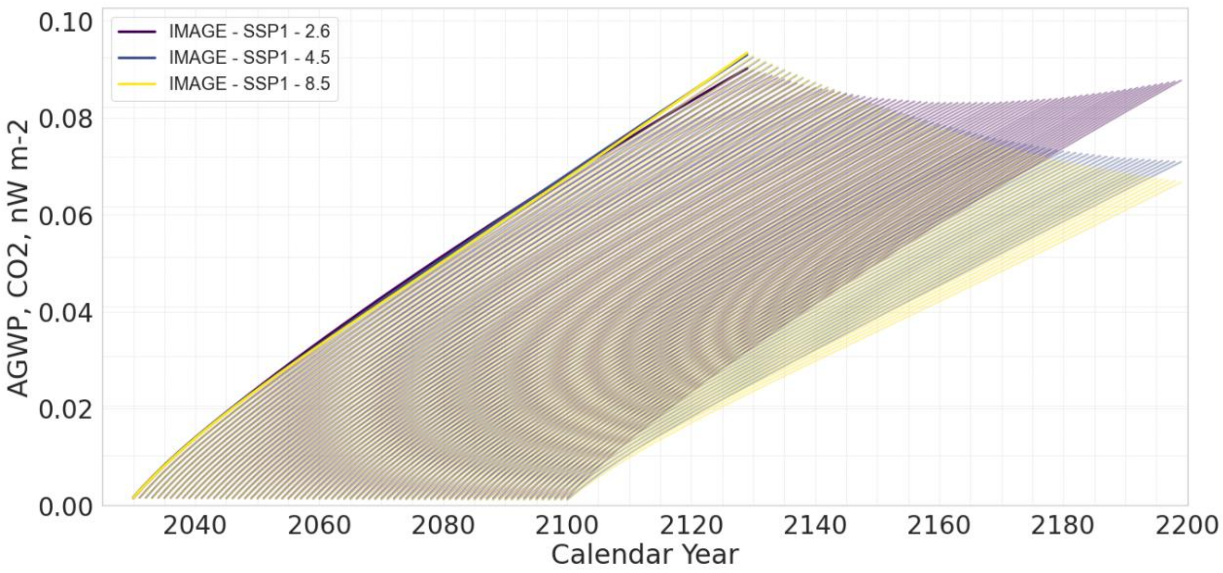

Figure S.2. Absolute global warming potential (AGWP) of CO2 under MESSAGE- Shared Socioeconomic Pathway 2 (SSP2) scenarios.

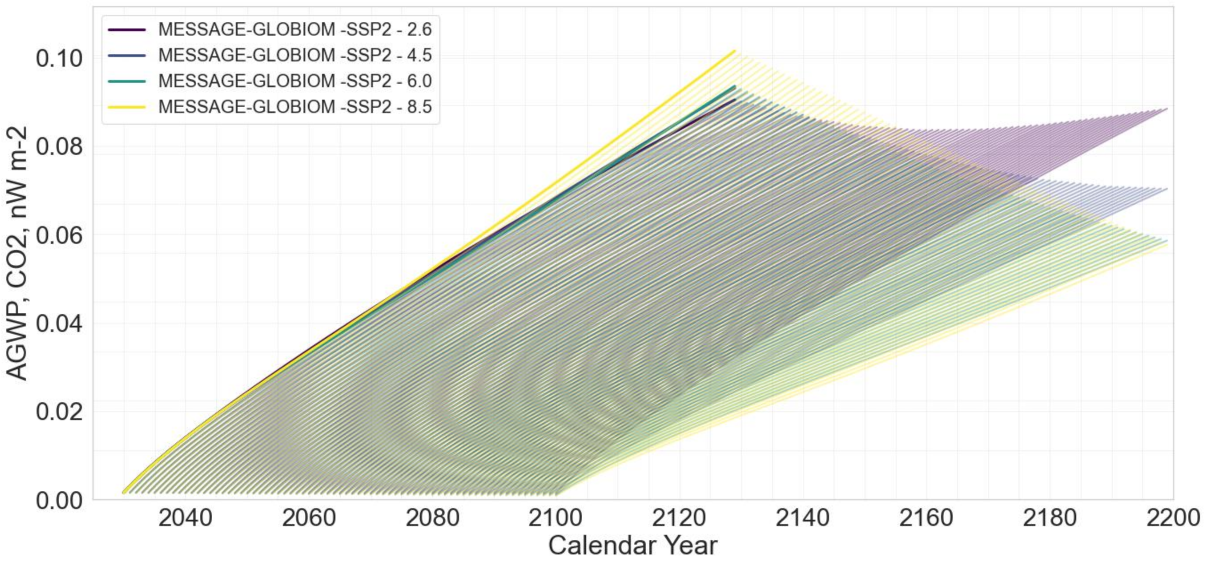

Figure S.3. Absolute global warming potential (AGWP) of CO_2 under AIM-CGE- Shared Socioeconomic Pathway 3 (SSP3) scenarios

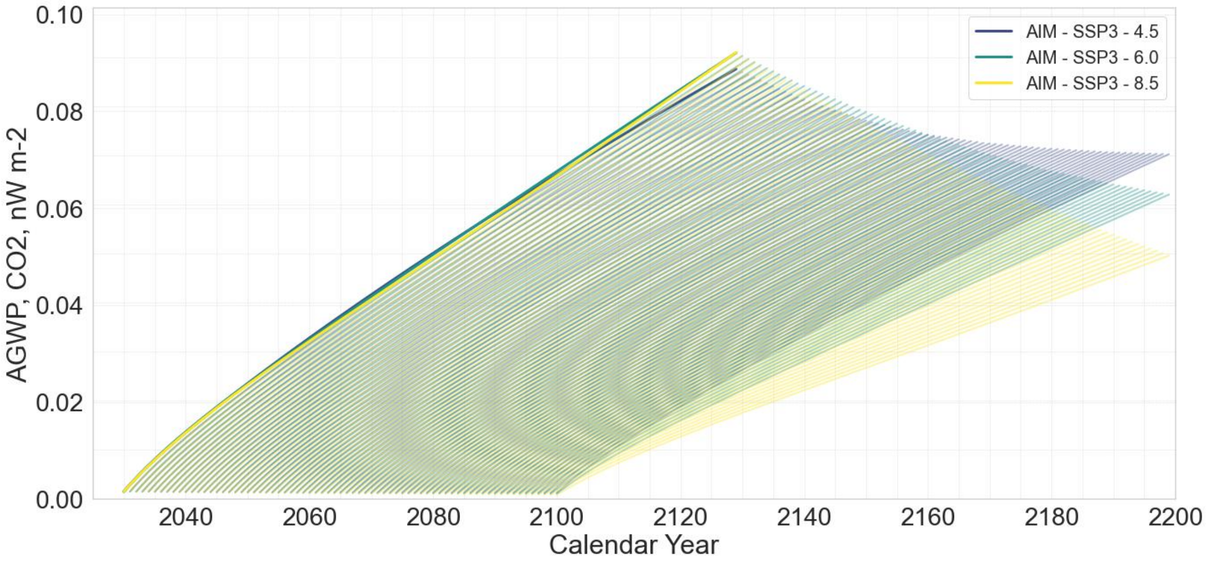

Figure S.4. Absolute global warming potential (AGWP) of CO_2 under GCAM4- Shared Socioeconomic Pathway 4 (SSP4) scenarios.

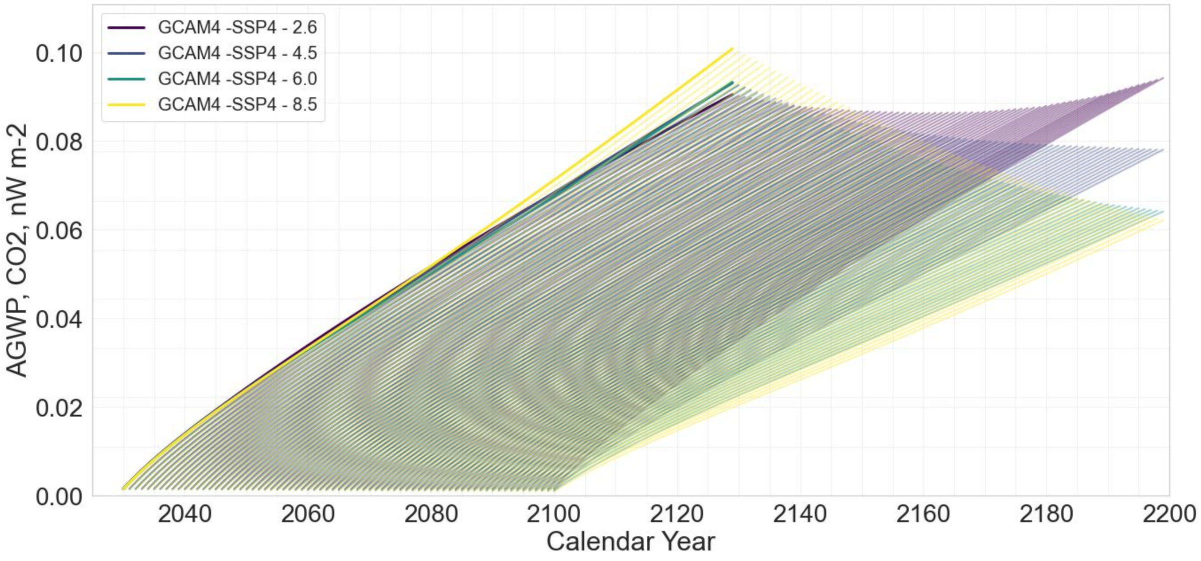

Figure S.5. Absolute global warming potential (AGWP) of CO_2 under REMIND- Shared Socioeconomic Pathway 5 (SSP5) scenarios.

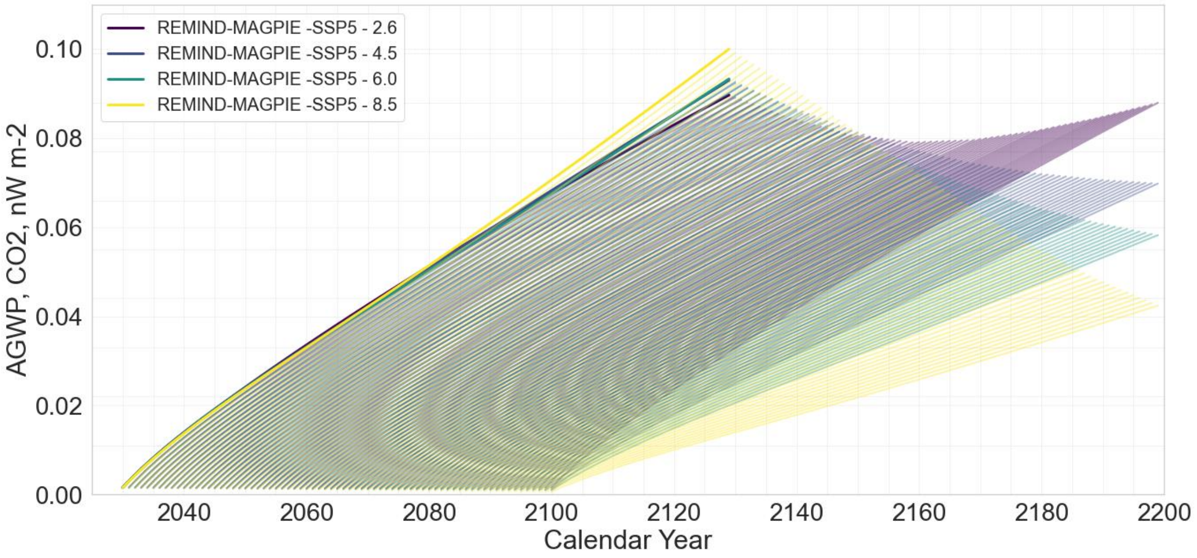

Figure S.6. Absolute global warming potential (AGWP) of CH_4 under IMAGE- Shared Socioeconomic Pathway 1 (SSP1) scenarios.

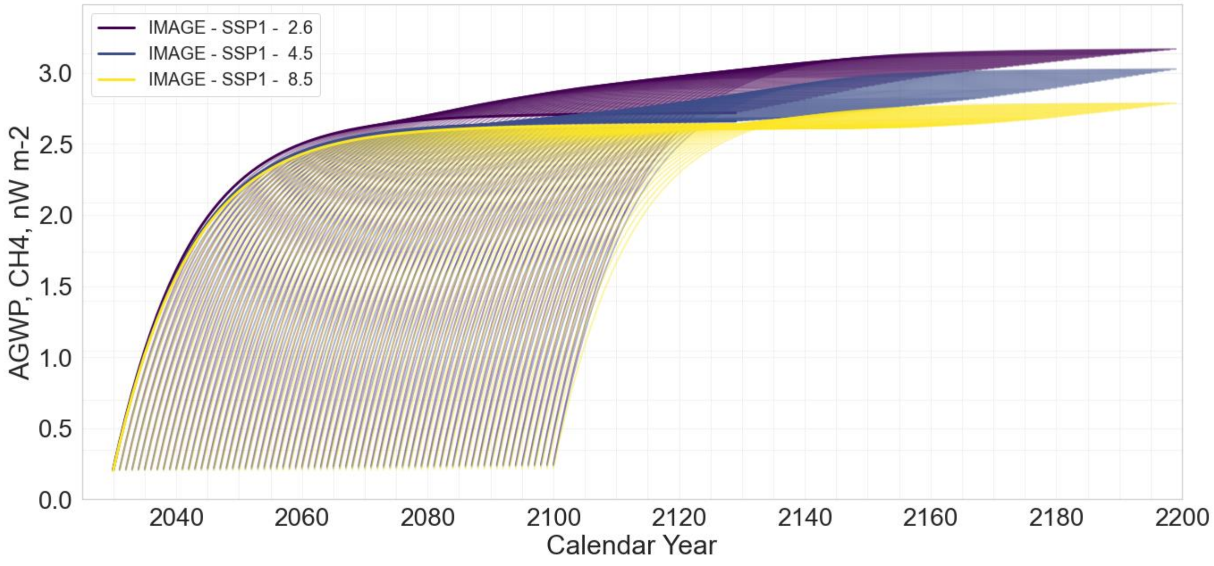

Figure S7. Absolute global warming potential (AGWP) of CH_4 under MESSAGE- Shared Socioeconomic Pathway 2 (SSP2) scenarios.

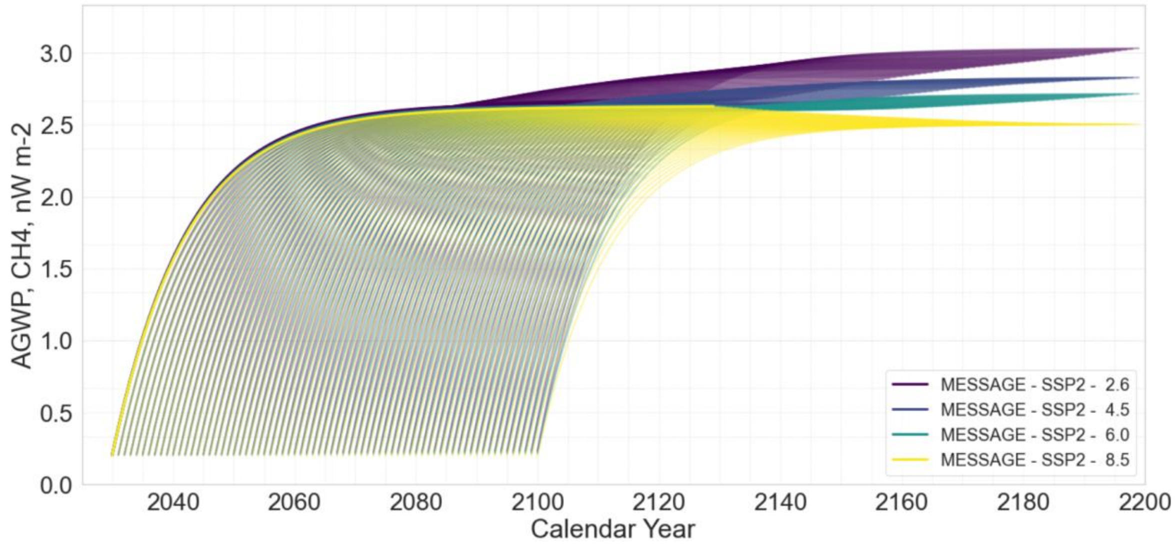

Figure S8. Absolute global warming potential (AGWP) of CH_4 under AIM-CGE- Shared Socioeconomic Pathway 3 (SSP3) scenarios.

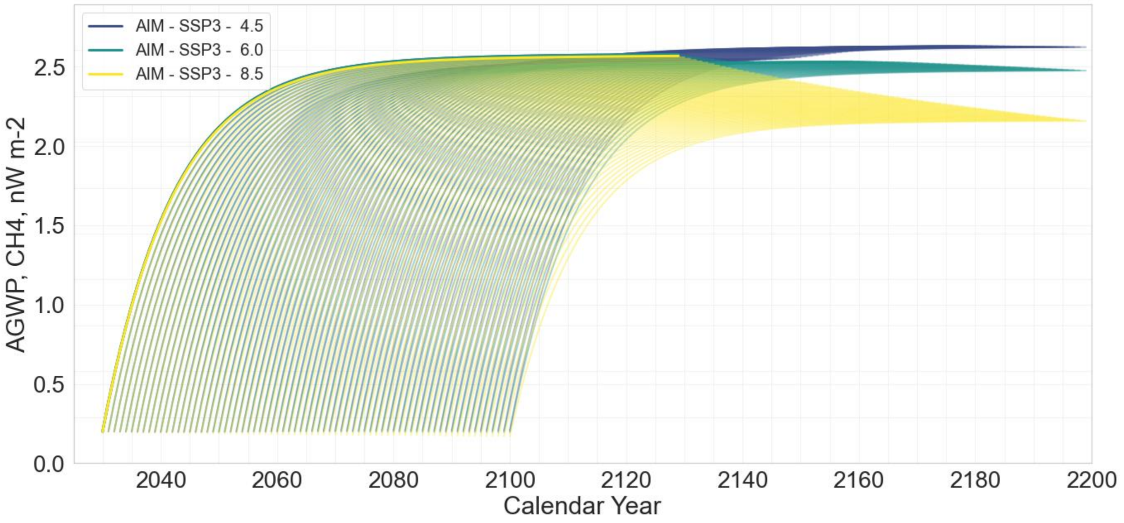

Figure S9. Absolute global warming potential (AGWP) of CH_4 under GCAM4- Shared Socioeconomic Pathway 4 (SSP4) scenarios

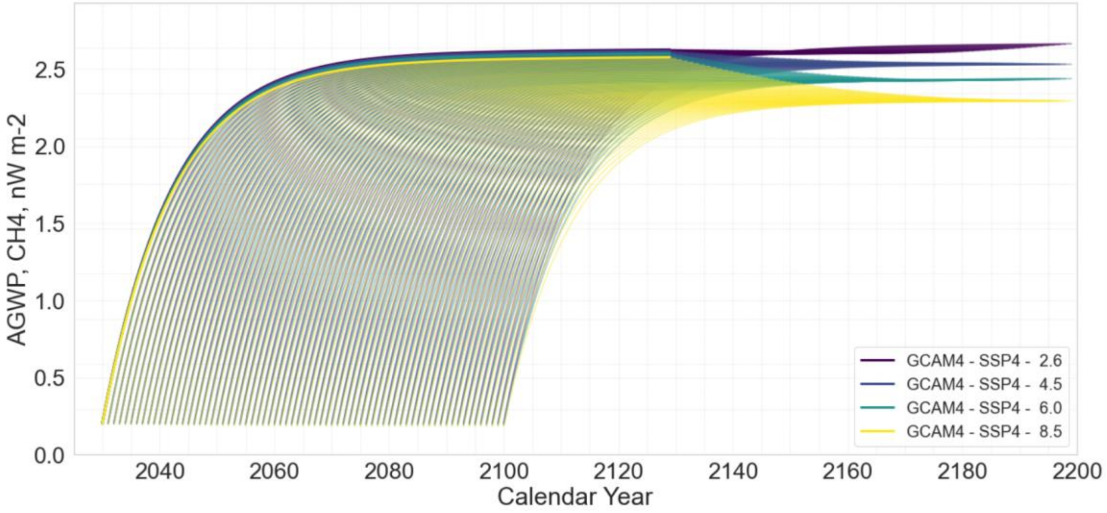

Figure S10. Absolute global warming potential (AGWP) of CH_4 under REMIND- Shared Socioeconomic Pathway 5 (SSP5) scenarios

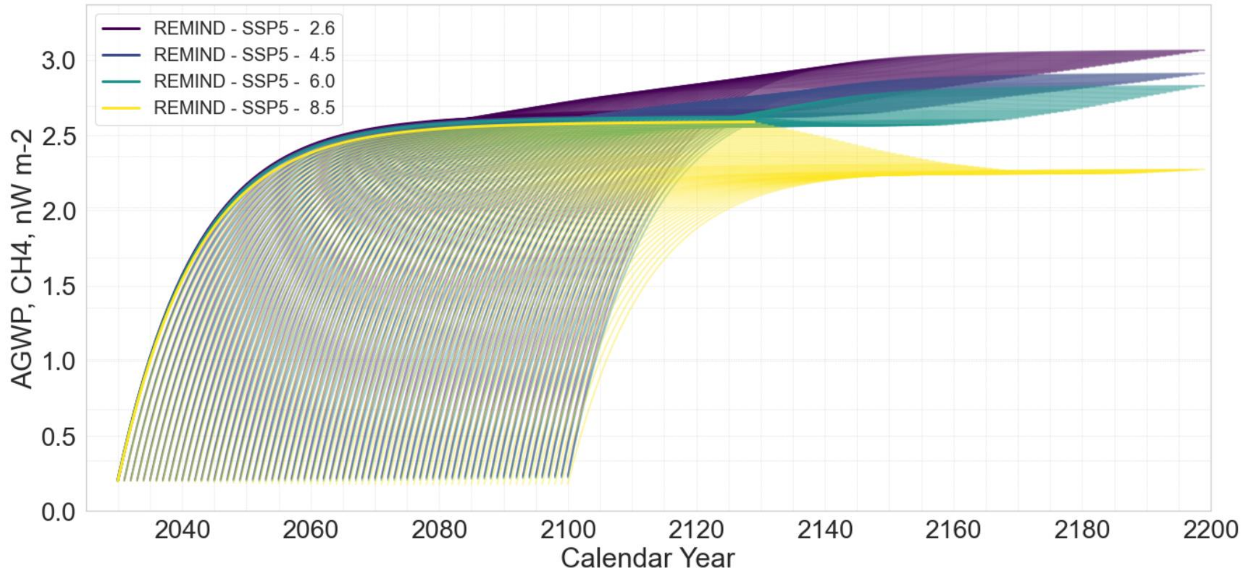

Figure S11. Absolute global warming potential (AGWP) of N_2O under IMAGE- Shared Socioeconomic Pathway 1 (SSP1) scenarios.

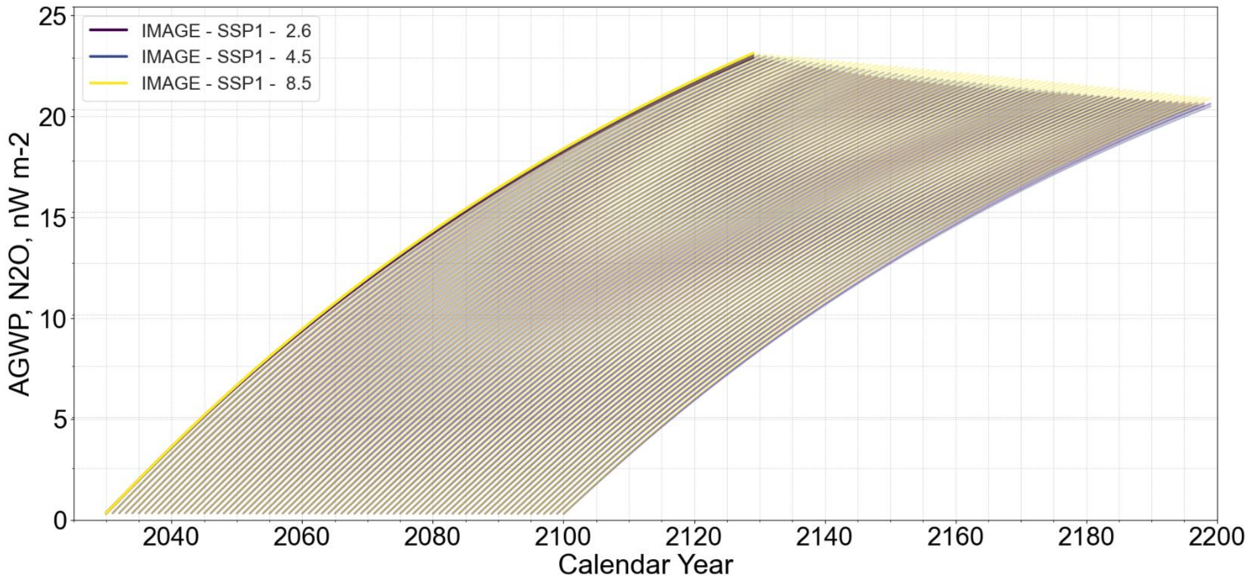

Figure S12. Absolute global warming potential (AGWP) of N_2O under MESSAGE- Shared Socioeconomic Pathway 2 (SSP2) scenarios.

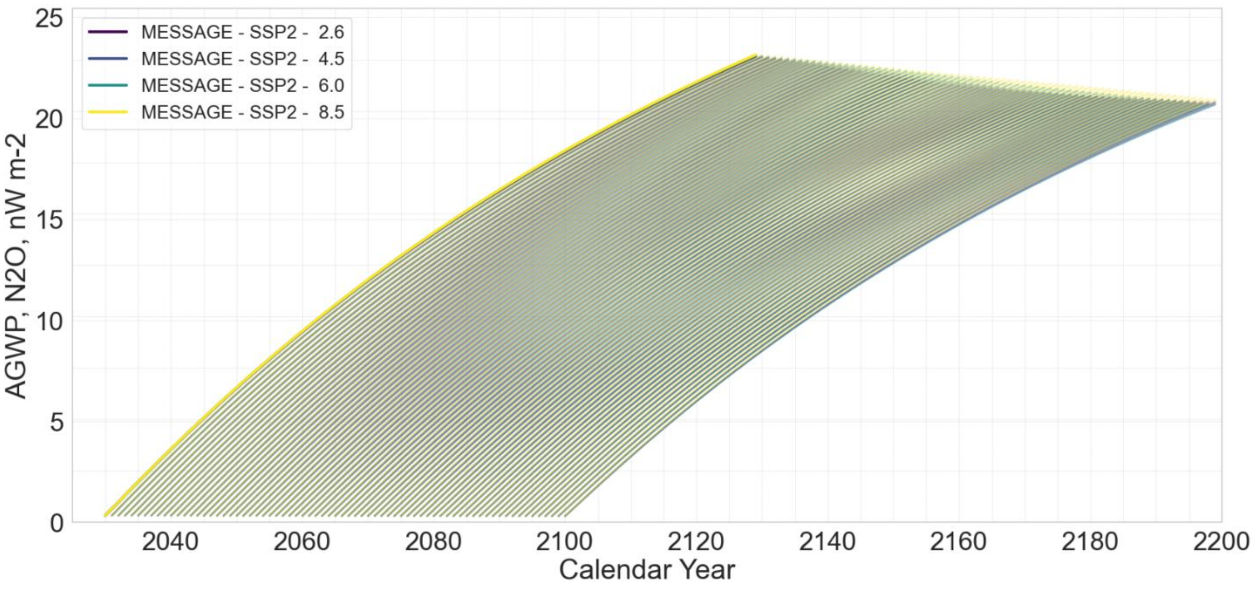

Figure S13. Absolute global warming potential (AGWP) of N_2O under AIM- Shared Socioeconomic Pathway 3 (SSP3) scenarios.

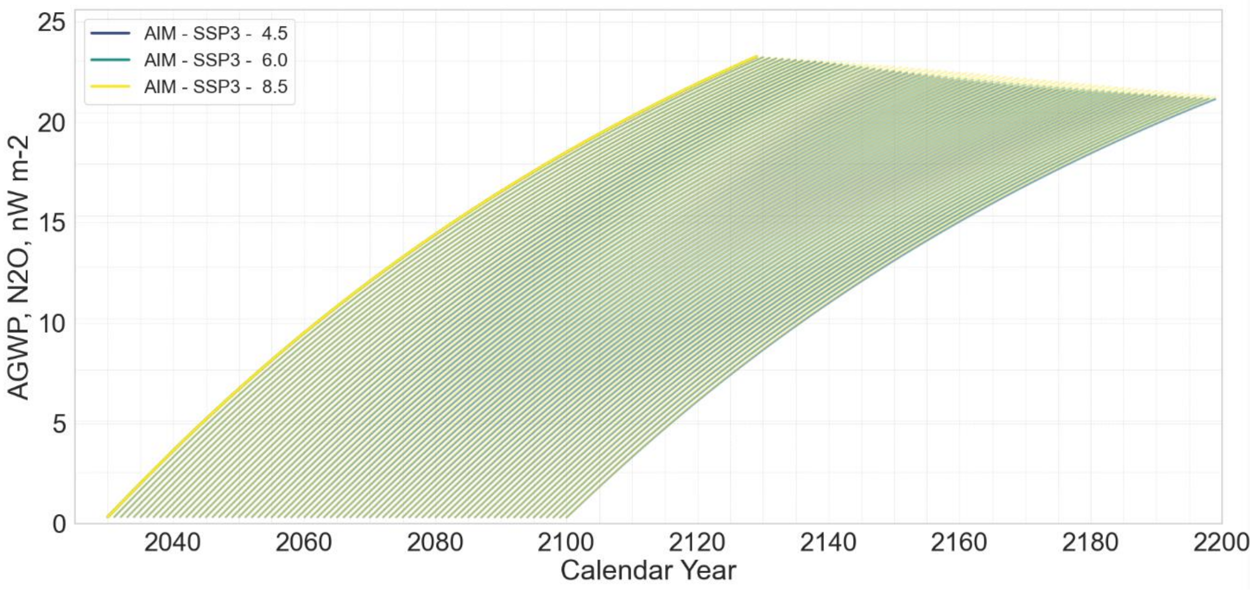

Figure S14. Absolute global warming potential (AGWP) of N_2O under GCAM4- Shared Socioeconomic Pathway 4 (SSP4) scenarios

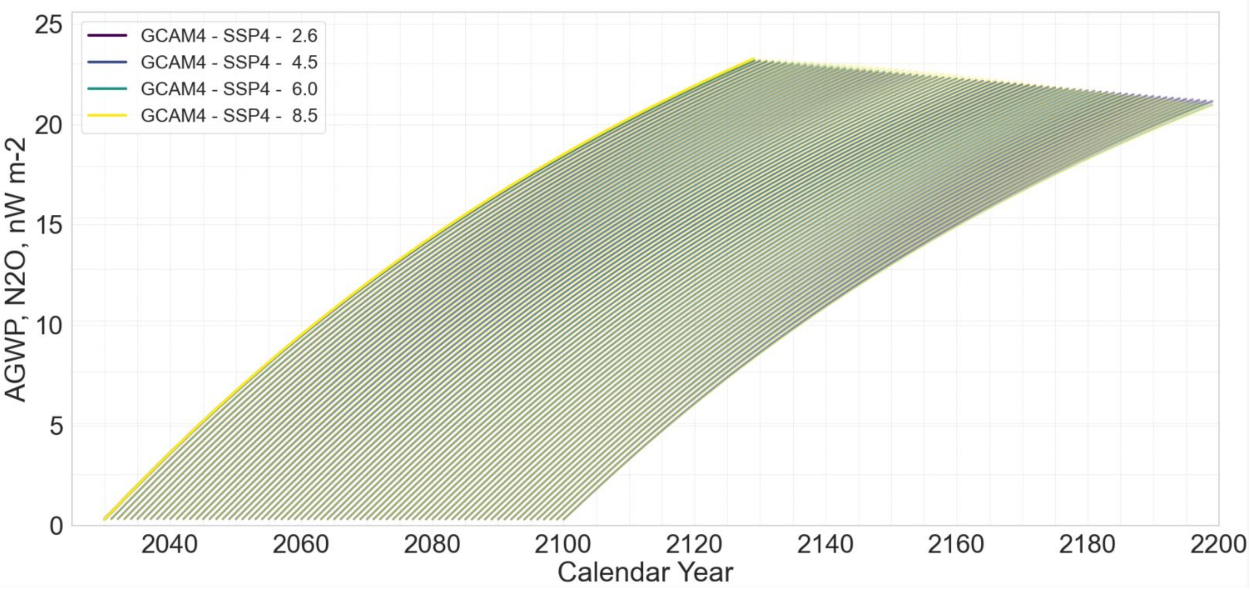

Figure S15. Absolute global warming potentials (AGWP) of N_2O under REMIND- Shared Socioeconomic Pathway 5 (SSP5) scenarios

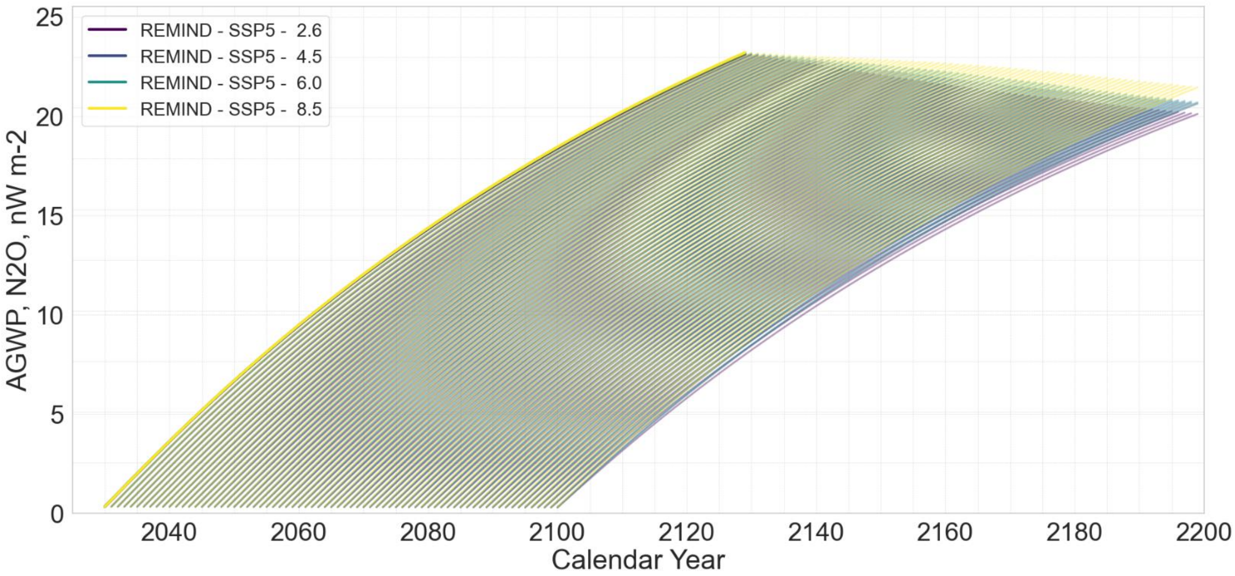

Figure S.16. Absolute global temperature change potential of CO_2 (AGTP) under IMAGE- Shared Socioeconomic Pathway 1 (SSP1) scenarios.

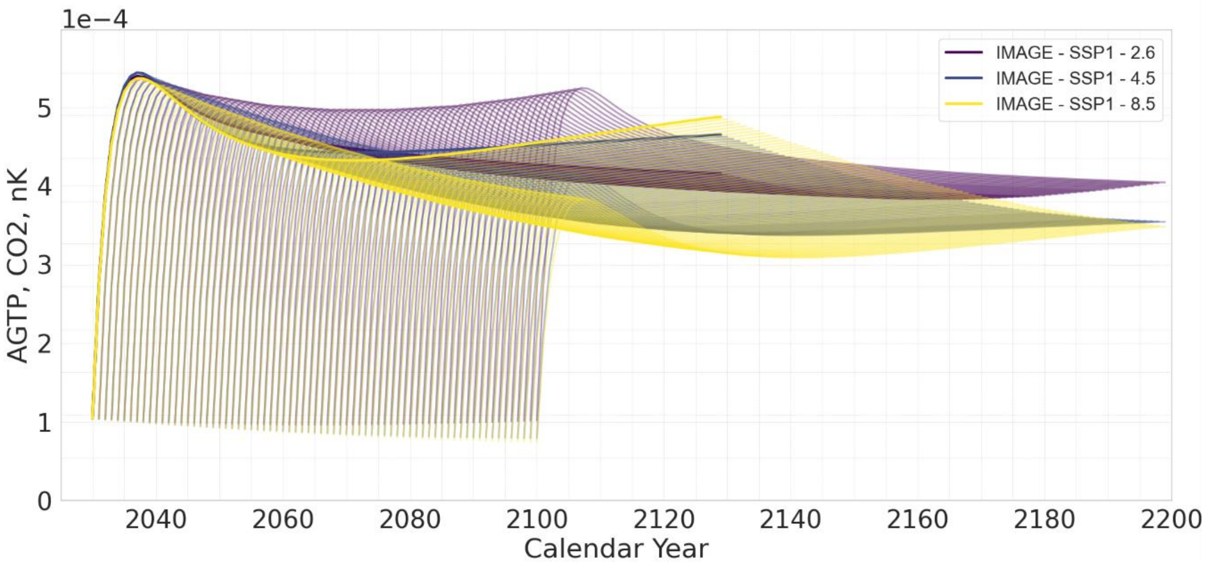

Figure S.17. Absolute global temperature change potential (AGTP) of CO_2 under MESSAGE- Shared Socioeconomic Pathway 2 (SSP2) scenarios

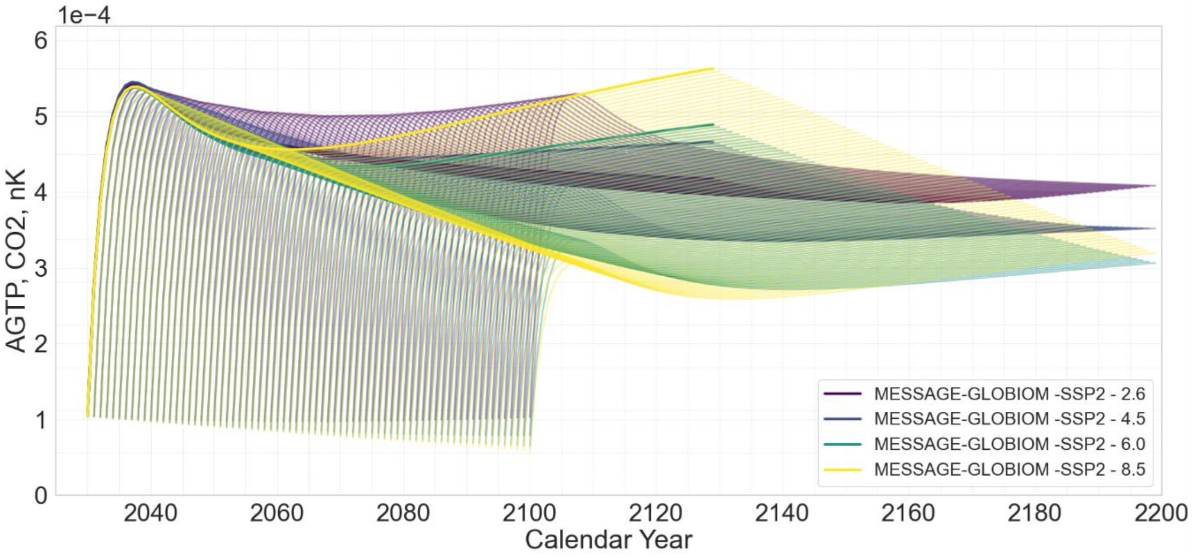

Figure S.18. Absolute global temperature change potential (AGTP) of CO_2 under AIM-CGE- Shared Socioeconomic Pathway 3 (SSP3) scenarios.

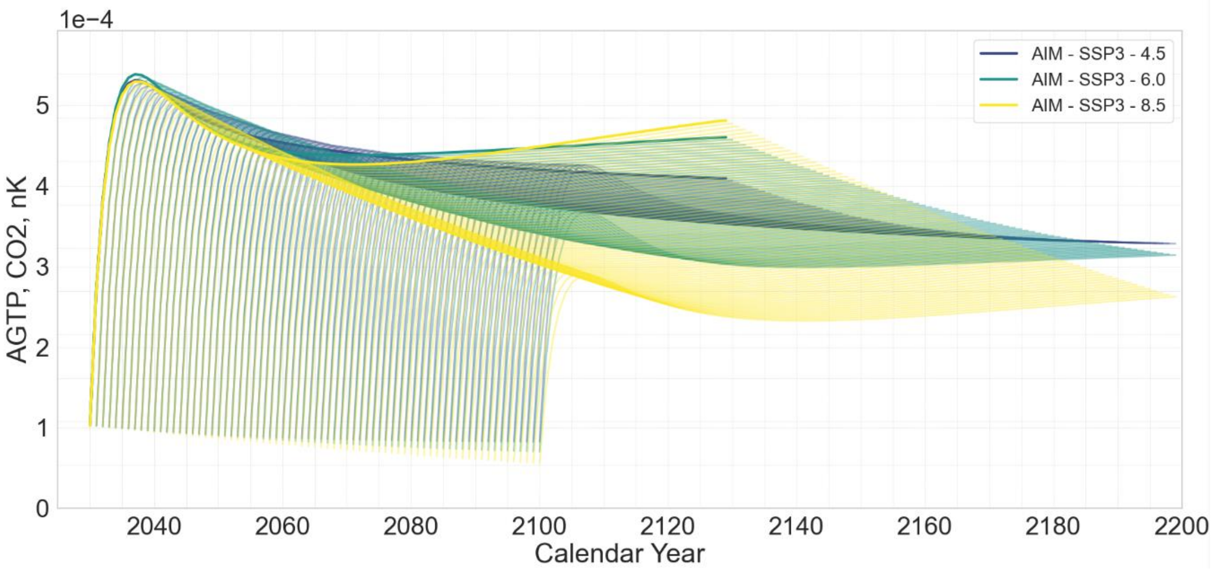

Figure S.19. Absolute global temperature change potential (AGTP) of CO_2 under GCAM4- Shared Socioeconomic Pathway 4 (SSP4) scenarios.

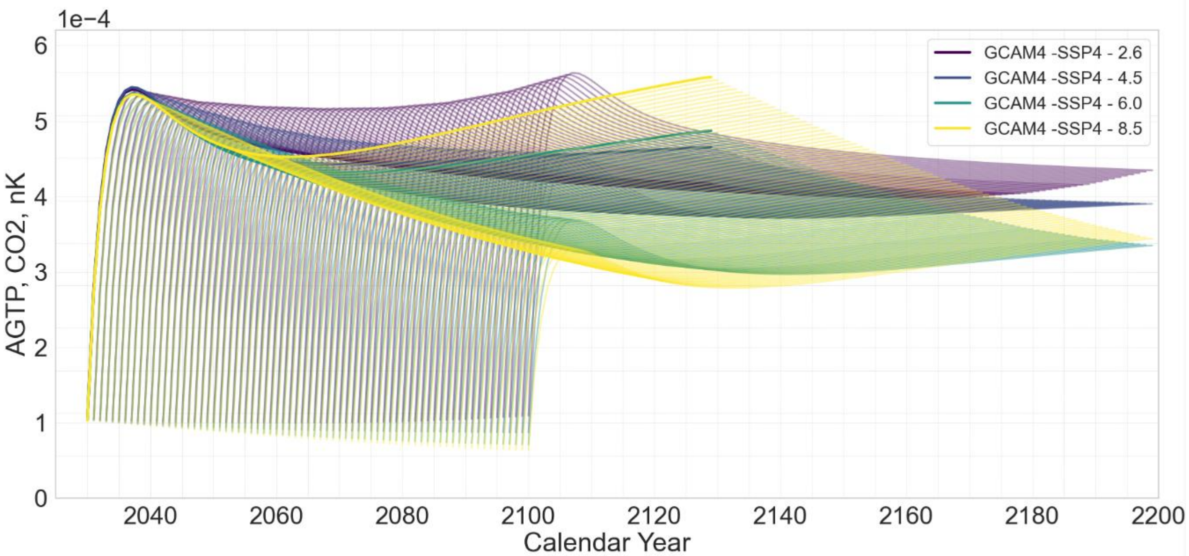

Figure S.20. Absolute global temperature change potential (AGTP) of CO2 under REMIND-Shared Socioeconomic Pathway 5 (SSP5) scenarios

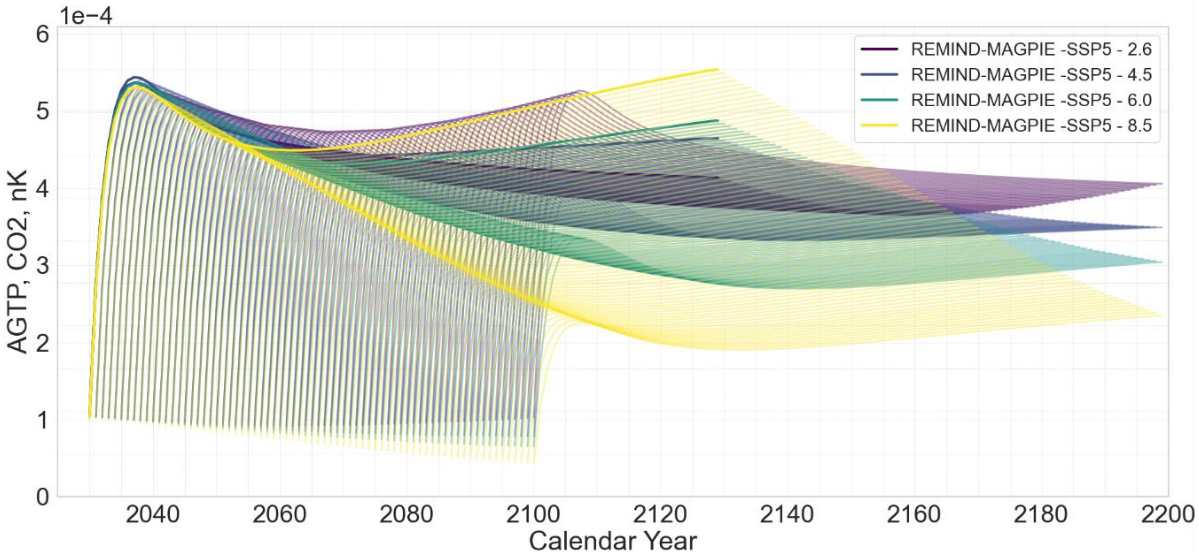

Figure S.21. Absolute global temperature change potential (AGTP) of CH_4 under IMAGE- Shared Socioeconomic Pathway 1 (SSP1) scenarios.

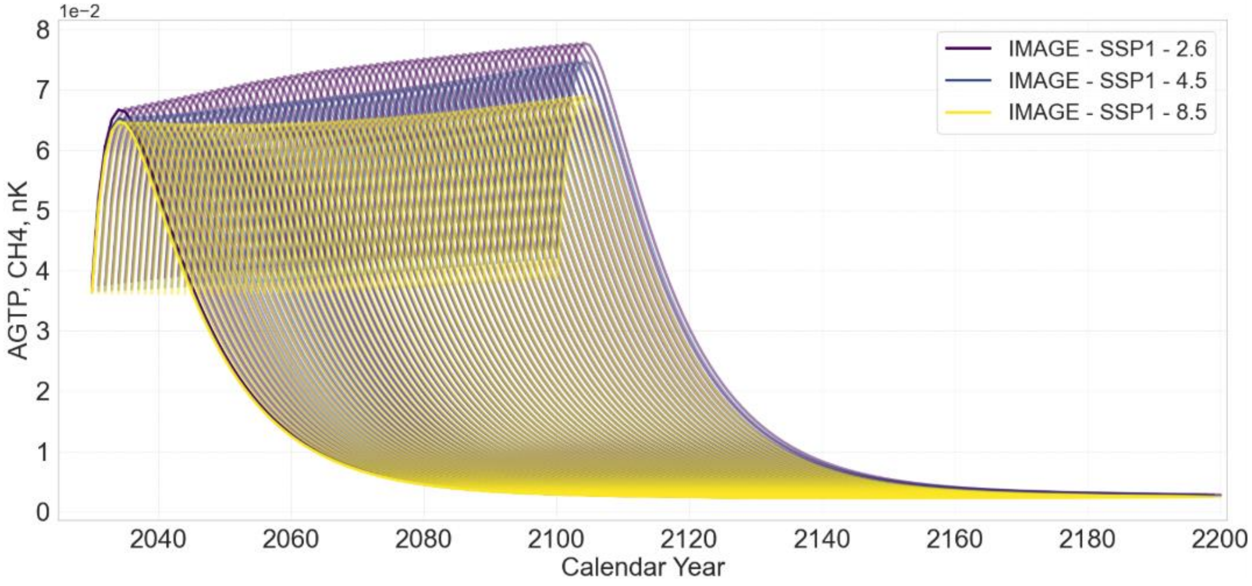

Figure S22. Absolute global temperature change potential (AGTP) of CH_4 under MESSAGE- Shared Socioeconomic Pathway 2 (SSP2) scenarios

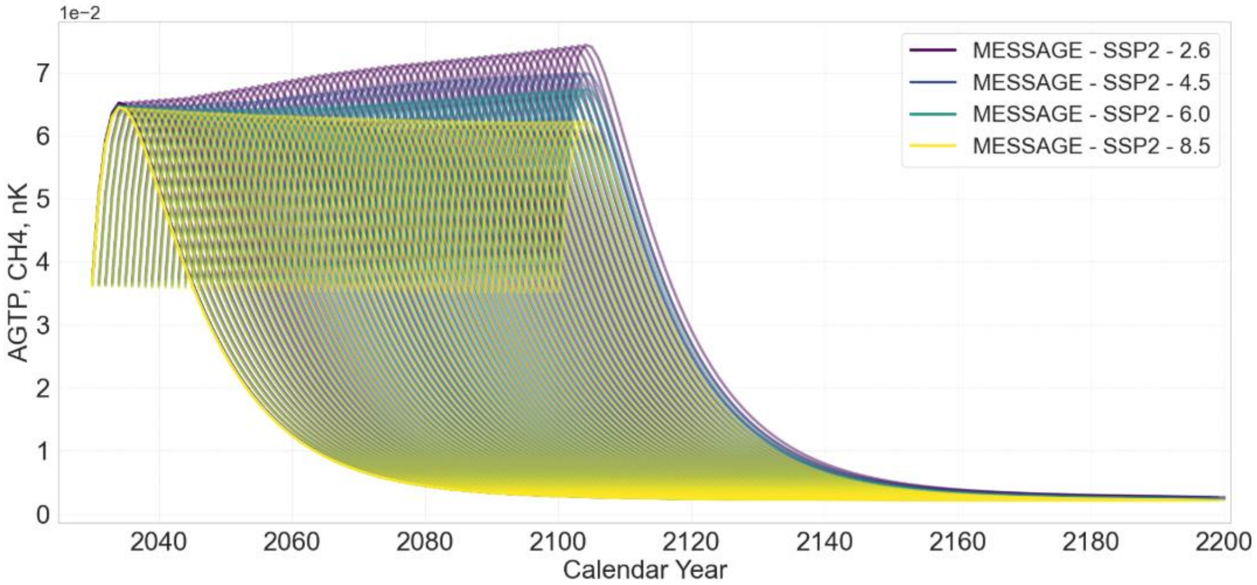

Figure S23. Absolute global temperature change potential (AGTP) of CH_4 under AIM-CGE- Shared Socioeconomic Pathway 3 (SSP3) scenarios

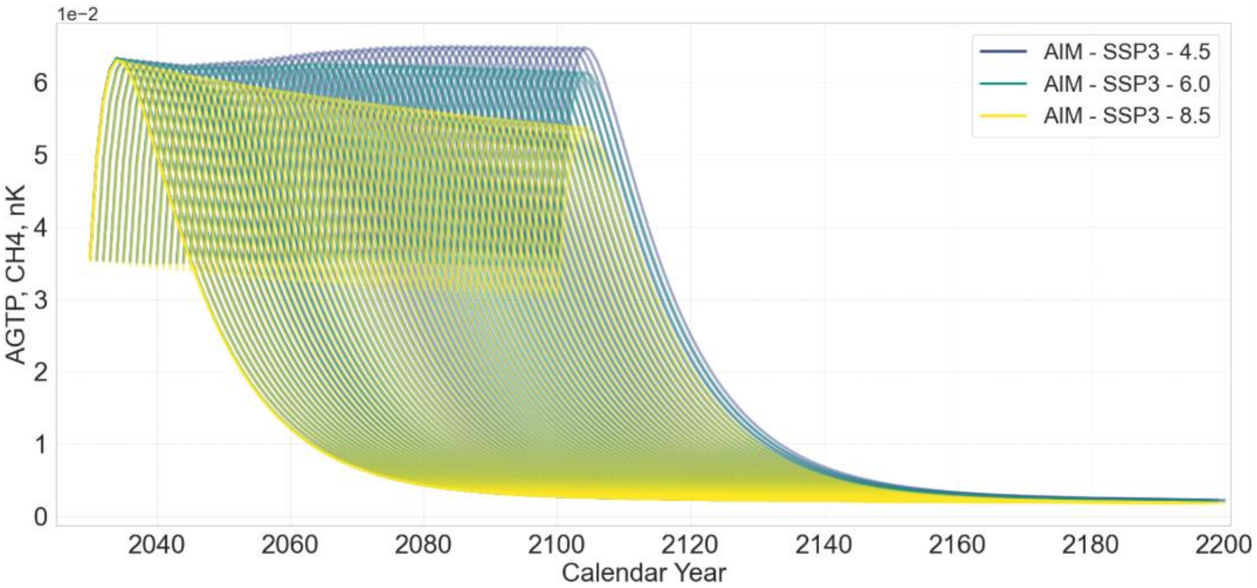

Figure S24. Absolute global temperature change potential (AGTP) of CH_4 under GCAM4- Shared Socioeconomic Pathway 4 (SSP4) scenarios.

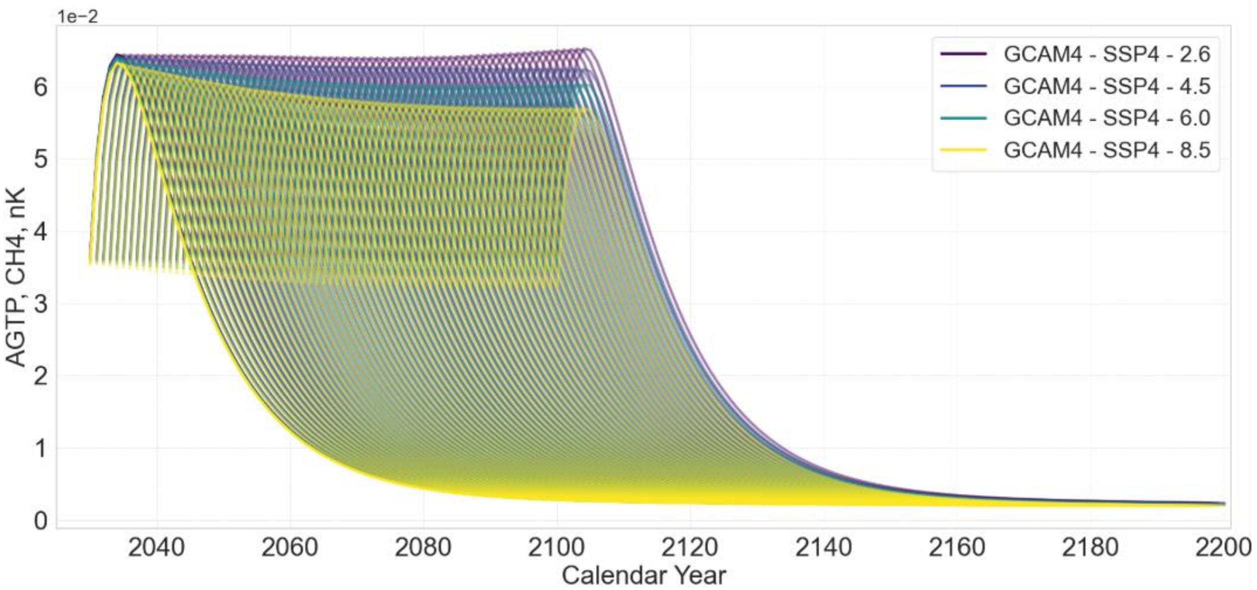

Figure S25. Absolute global temperature change potential (AGTP) of CH_4 under REMIND- Shared Socioeconomic Pathway 5 (SSP5) scenarios.

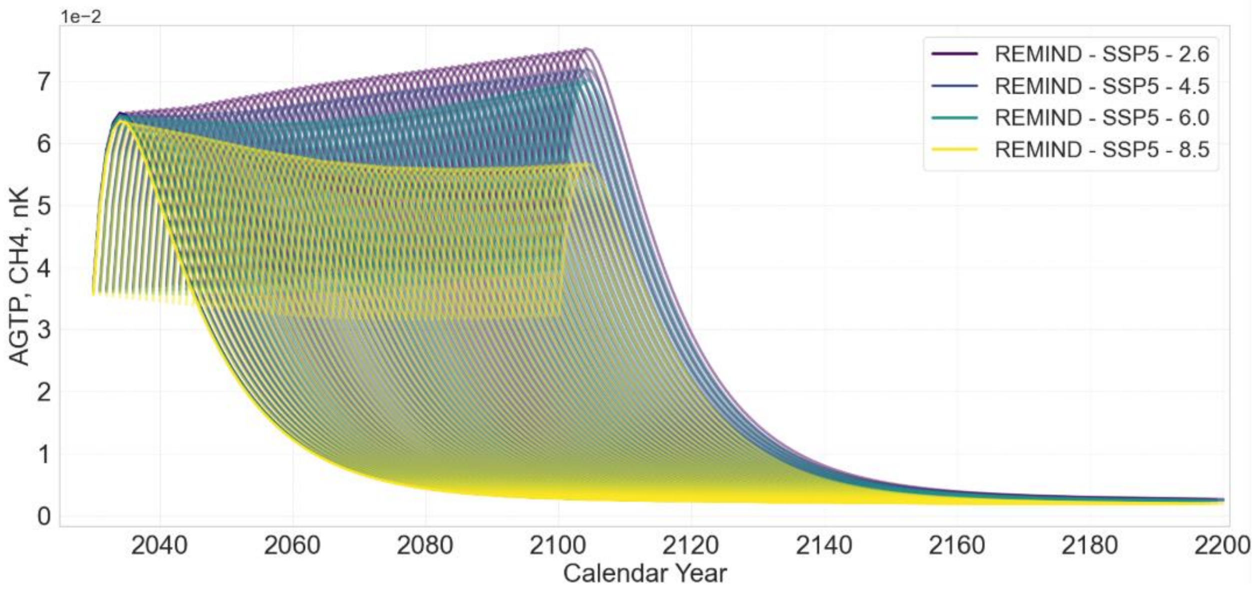

Figure S26. Absolute global temperature change potential (AGTP) of N_2O under IMAGE- Shared Socioeconomic Pathway 1 (SSP1) scenarios.

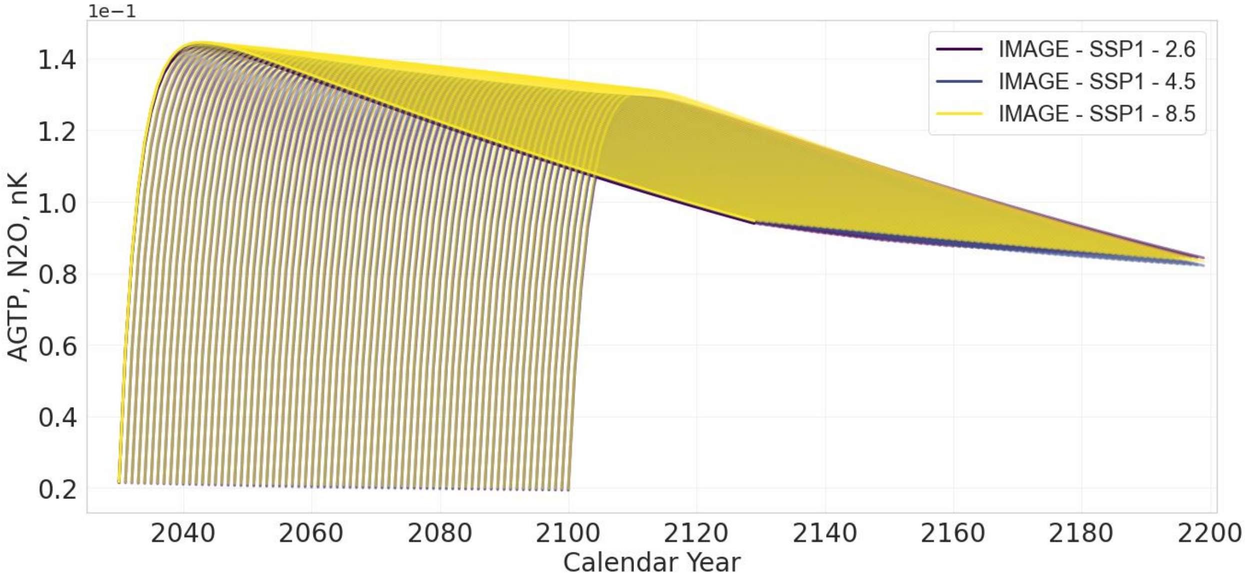

Figure S27. Absolute global temperature change potential (AGTP) of N2O under MESSAGE- Shared Socioeconomic Pathway 2 (SSP2) scenarios

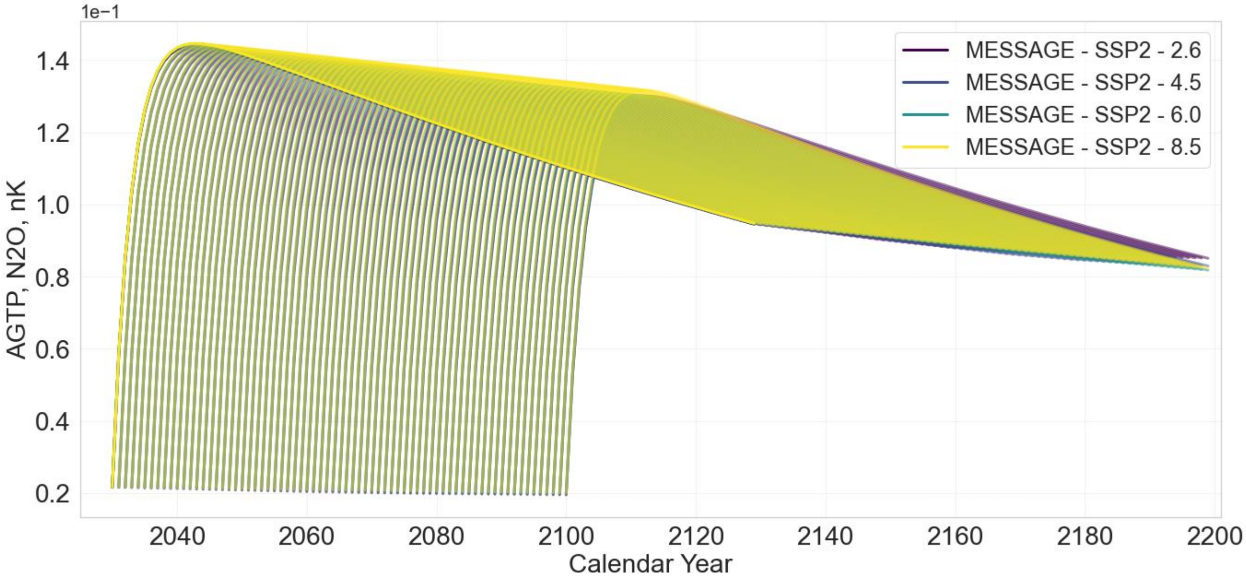

Figure S28. Absolute global temperature change potential (AGTP) of N_2O under AIM- Shared Socioeconomic Pathway 3 (SSP3) scenarios.

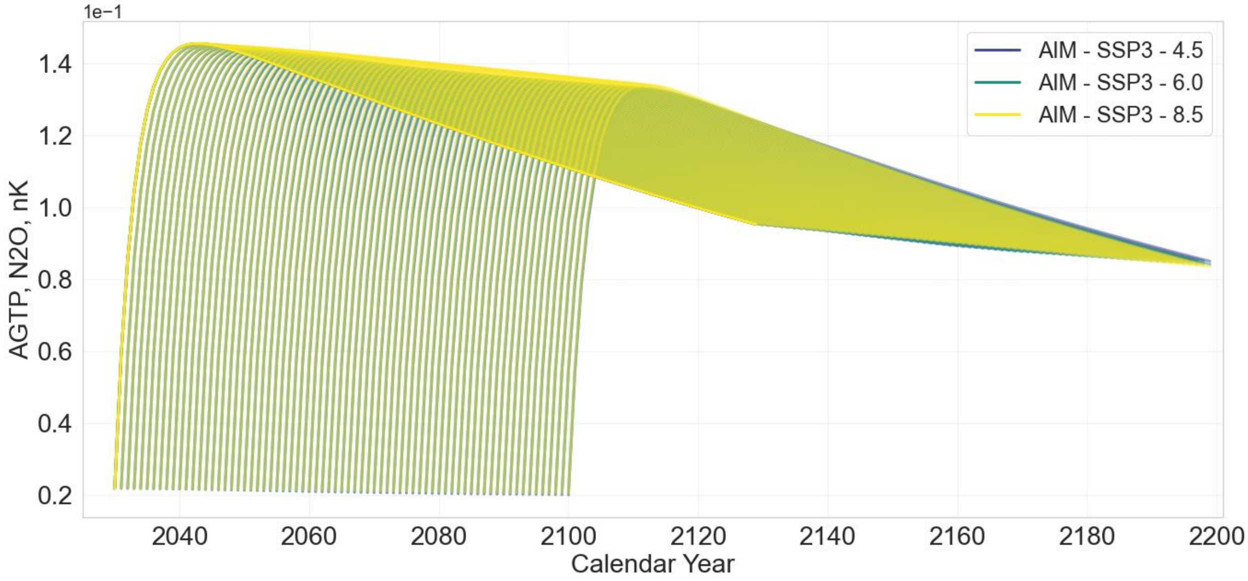

Figure S29. Absolute global temperature change potential (AGTP) of N_2O under GCAM4- Shared Socioeconomic Pathway 4 (SSP4) scenarios

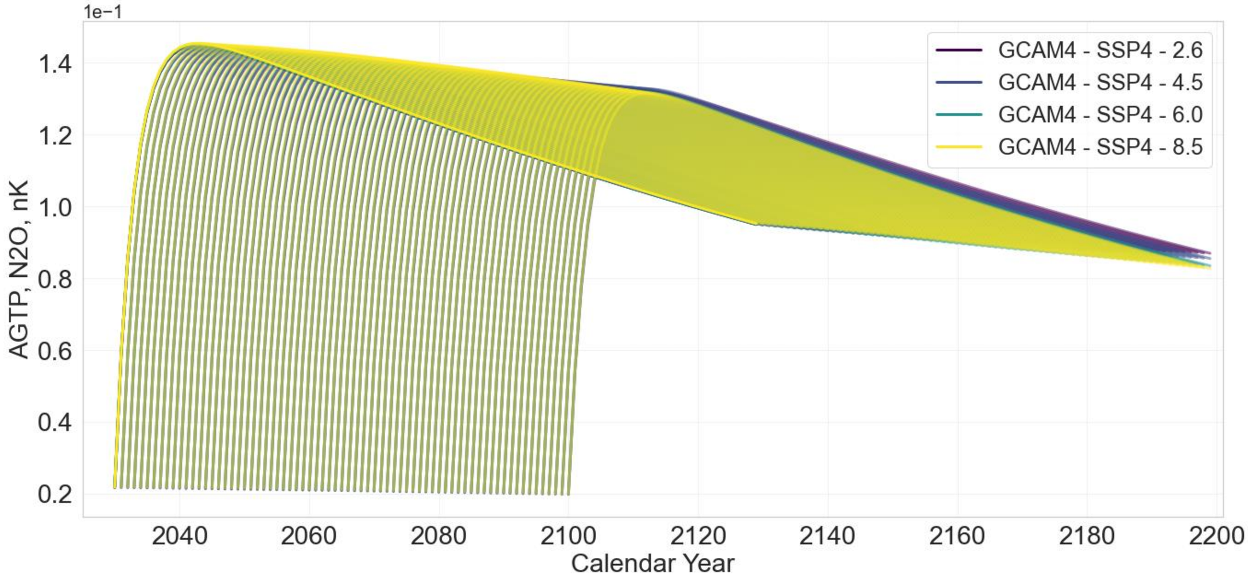

Figure S30. Absolute global temperature change potential (AGTP) of N_2O under REMIND- Shared Socioeconomic Pathway 5 (SSP5) scenarios.

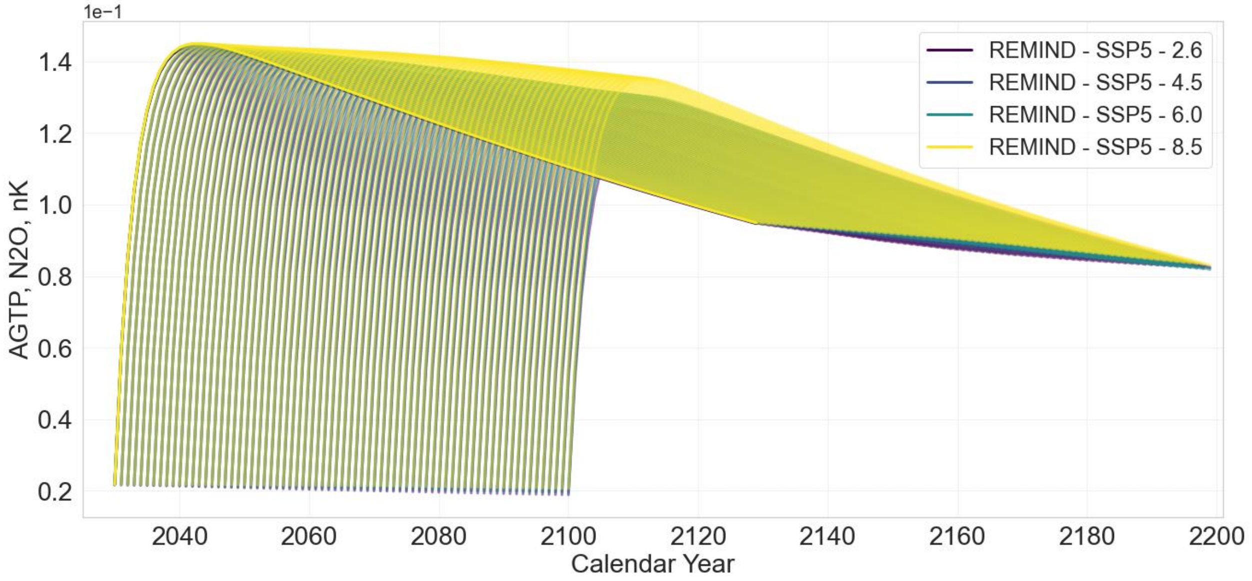

Figure S31. Comparison of 'rice production, non-basmati' in India and the United States. Foreground inventories obtained from PREMISE scenario REMIND-SSP5-RCP8.5 for the year 2040. Panels (a) and (c) present results using the current IPCC (2021) characterization factors for GTP_{100}, while panels (b) and (d) show results based on prospective characterization factors for $pGTP_{100}$.

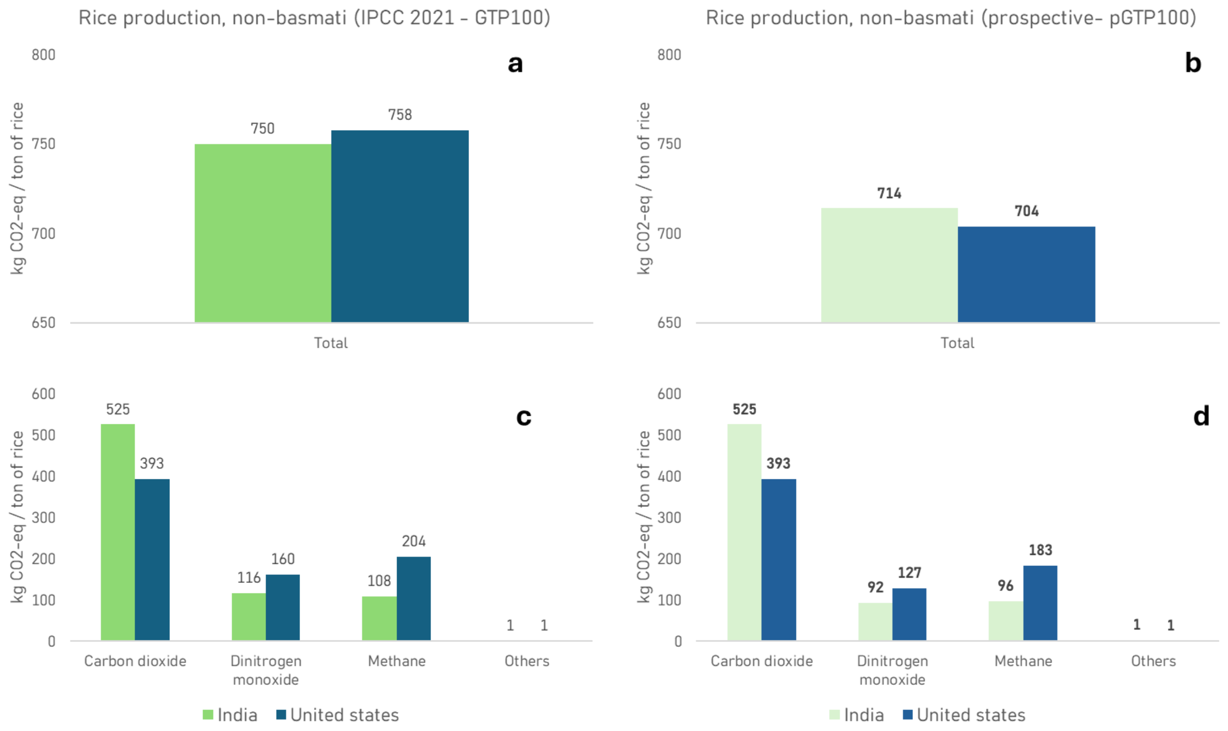

Figure S32. Comparison of 'market for nitric acid, without water, in 50% solution state' in UN-Oceania and Rest of the World (RoW). Foreground inventories were obtained from the PREMISE scenario REMIND-SSP5-RCP8.5 for the year 2040. Panels (a) and (c) present results using the current IPCC (2021) characterization factors for GTP_{100}, while panels (b) and (d) show results based on prospective characterization factors for $pGTP_{100}$.

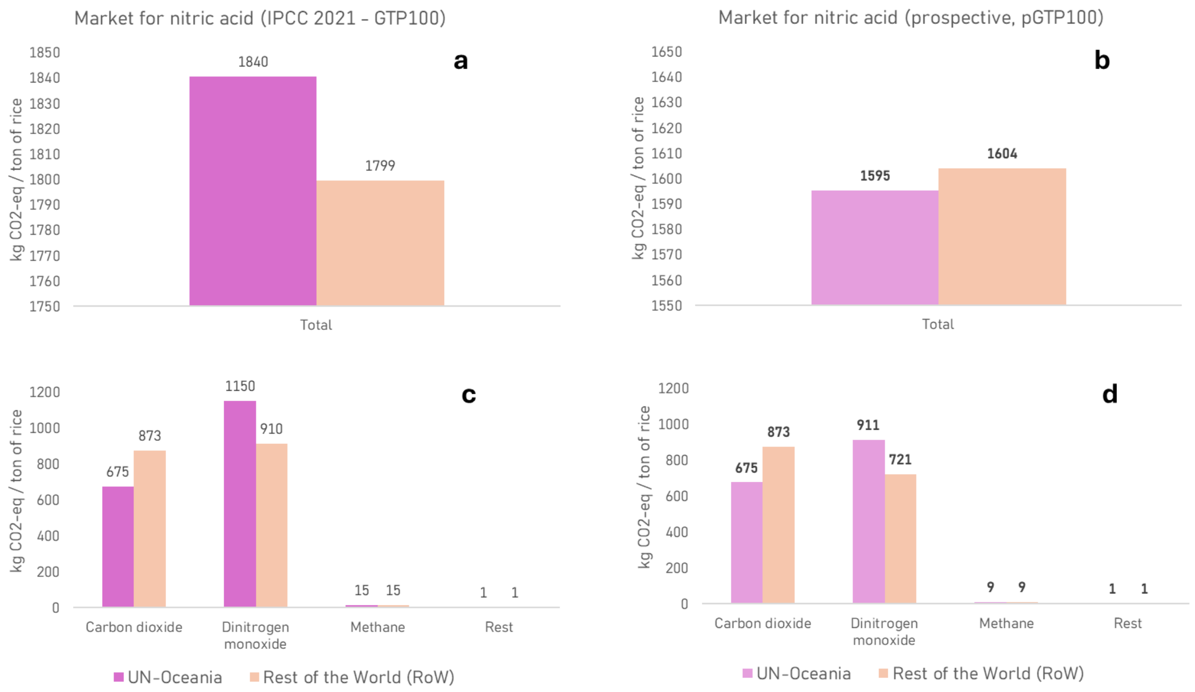

Table S.2. Results for CH_4 $pGWP_{100}$ under different IAM-SSP-RCP scenarios.

**Scenario, $pGWP_{100}$ - CH_4**	**2030**	**2035**	**2040**	**2045**	**2050**
AIM_-_SSP3_-_4.5	29.1	29.8	30.6	31.6	32.6
AIM_-_SSP3_-_6.0	28	28.7	29.5	30.4	31.4
AIM_-_SSP3_-_8.5	27.9	28.5	29.2	30	30.9
GCAM4_-SSP4_-_2.6	29	29.4	29.8	30	30.2
GCAM4_-SSP4_-_4.5	28.1	28.7	29.4	29.9	30.5
GCAM4_-SSP4_-_6.0	27.9	28.5	29.1	29.8	30.6
GCAM4_-SSP4_-_8.5	25.6	26.1	26.6	27.2	27.9
IMAGE_-_SSP1_-_2.6	30.1	31.3	32.5	33.5	34.5
IMAGE_-_SSP1_-_4.5	28.6	29.6	30.7	31.8	32.9
IMAGE_-_SSP1_-_8.5	28.2	29	29.8	30.7	31.6
MESSAGE_-SSP2_-_2.6	29.4	30.2	31	31.8	32.6
MESSAGE_-SSP2_-_4.5	28.4	29.2	30.1	31.1	32.2
MESSAGE_-SSP2_-_6.0	28.2	29	29.8	30.8	31.8
MESSAGE_-SSP2_-_8.5	25.9	26.6	27.3	28.2	29.1
REMIND_-SSP5_-_2.6	29.5	30.6	31.7	32.9	34.1
REMIND_-SSP5_-_4.5	28.3	29.2	30.2	31.3	32.5
REMIND_-SSP5_-_6.0	28.1	28.9	29.8	30.9	32
REMIND_-SSP5_-_8.5	25.9	26.6	27.3	28.2	29.1
**Statistics, $pGWP_{100}$ - CH_4**	**2030**	**2035**	**2040**	**2045**	**2050**
Mean - ALL RCPs	28.1	28.9	29.7	30.5	31.4
Std. Deviation - ALL RCPs	1.2	1.3	1.5	1.6	1.7
Mean - RCP 2.6	29.5	30.4	31.2	32.0	32.8
Std. Deviation - RCP2.6	0.5	0.8	1.1	1.5	1.9
Mean - RCP 4.5	28.5	29.3	30.2	31.1	32.1
Std. Deviation - RCP4.5	0.4	0.4	0.5	0.7	1.0
Mean - RCP 6.0	28.0	28.8	29.5	30.5	31.4
Std. Deviation - RCP6.0	0.1	0.2	0.3	0.5	0.6
Mean - RCP8.5	26.7	27.3	28.0	28.8	29.7
Std. Deviation - RCP8.5	1.1	1.2	1.2	1.3	1.3

Table S.3. Results for N_2O $pGWP_{100}$ under different IAM-SSP-RCP scenarios.

**Scenario, $pGWP_{100}$ - N_2O**	**2030**	**2035**	**2040**	**2045**	**2050**
AIM_-_SSP3_-_4.5	262.2	269.5	277.2	282.4	287.8
AIM_-_SSP3_-_6.0	252.7	260.1	268.1	274.6	281.5
AIM_-_SSP3_-_8.5	252.7	261.6	271.4	281.3	292
GCAM4_-SSP4_-_2.6	256.2	258.2	260.2	260.2	260.2
GCAM4_-SSP4_-_4.5	249.1	254.3	259.8	263.9	268.1
GCAM4_-SSP4_-_6.0	249	256	263.5	270.7	278.4
GCAM4_-SSP4_-_8.5	230.8	238.3	246.4	254.4	263.1
IMAGE_-_SSP1_-_2.6	254.2	256.6	259.2	259.9	260.6
IMAGE_-_SSP1_-_4.5	248	252.9	258.2	262.7	267.4
IMAGE_-_SSP1_-_8.5	247.7	253.7	260.2	266.1	272.4
MESSAGE_-SSP2_-_2.6	255.1	257.9	260.7	261.2	261.6
MESSAGE_-SSP2_-_4.5	248.1	253.6	259.4	264.5	269.9
MESSAGE_-SSP2_-_6.0	247.3	253.5	260.2	267.1	274.4
MESSAGE_-SSP2_-_8.5	228	234	240.5	247.4	254.9
REMIND_-SSP5_-_2.6	257.9	262.8	268.1	271	274
REMIND_-SSP5_-_4.5	249.1	255.3	261.9	267.8	274
REMIND_-SSP5_-_6.0	248.5	255.9	264	272.7	282
REMIND_-SSP5_-_8.5	232	241.5	251.8	264.3	278.2
**Statistics, GWP_{100} - N_2O**	**2030**	**2035**	**2040**	**2045**	**2050**
Mean - ALL RCPs	248.1	254.1	260.5	266.1	272.1
Std. Deviation - ALL RCPs	9.3	8.6	8.5	8.6	10.1
Mean - RCP 2.6	255.8	258.9	262.0	263.0	264.0
Std. Deviation - RCP2.6	1.6	2.7	4.1	5.3	6.6
Mean - RCP 4.5	251.2	257.0	263.2	268.2	273.3
Std. Deviation - RCP4.5	6.1	7.0	7.9	8.1	8.4
Mean - RCP 6.0	249.4	256.4	263.9	271.3	279.1
Std. Deviation - RCP6.0	2.3	2.7	3.2	3.2	3.5
Mean - RCP8.5	238.0	245.6	253.8	262.4	271.8
Std. Deviation - RCP8.5	10.0	10.3	10.8	11.5	12.7

Table S.4. Results for CH_4 $pGWP_{20}$ under different IAM-SSP-RCP scenarios.

**Scenario, $pGWP_{20}$ - CH_4**	**2030**	**2035**	**2040**	**2045**	**2050**
AIM_-_SSP3_-_4.5	91.1	93.4	95.9	99	102.3
AIM_-_SSP3_-_6.0	91.2	93.5	95.9	99.1	102.4
AIM_-_SSP3_-_8.5	91.6	93.7	96.1	98.7	101.6
GCAM4_-SSP4_-_2.6	90.9	92.1	93.4	93.9	94.5
GCAM4_-SSP4_-_4.5	91.4	93.5	95.6	97.4	99.2
GCAM4_-SSP4_-_6.0	91.6	93.6	95.7	98	100.5
GCAM4_-SSP4_-_8.5	90.7	92.5	94.5	96.7	99.2
IMAGE_-_SSP1_-_2.6	94.4	98	101.8	104.9	108.1
IMAGE_-_SSP1_-_4.5	93.1	96.3	99.8	103.5	107.3
IMAGE_-_SSP1_-_8.5	92.7	95.3	98	100.8	103.8
MESSAGE_-SSP2_-_2.6	92	94.5	97	99.6	102.1
MESSAGE_-SSP2_-_4.5	92.3	95.1	98	101.3	104.9
MESSAGE_-SSP2_-_6.0	92.5	95.2	98	101.2	104.7
MESSAGE_-SSP2_-_8.5	91.8	94.3	97	100.1	103.4
REMIND_-SSP5_-_2.6	92.4	95.8	99.4	103.1	106.9
REMIND_-SSP5_-_4.5	92.1	95	98.2	101.9	105.8
REMIND_-SSP5_-_6.0	92.3	95	97.9	101.4	105.2
REMIND_-SSP5_-_8.5	91.9	94.3	97.1	100.2	103.6
**Statistics, $pGWP_{20}$ - CH_4**	**2030**	**2035**	**2040**	**2045**	**2050**
Mean - ALL RCPs	92.0	94.5	97.2	100.0	103.0
Std. Deviation - ALL RCPs	0.9	1.4	2.0	2.6	3.4
Mean - RCP 2.6	92.4	95.1	97.9	100.3	102.8
Std.Deviation - RCP2.6	1.5	2.5	3.6	4.8	6.2
Mean - RCP 4.5	92.0	94.7	97.5	100.6	103.9
Std. Deviation - RCP4.5	0.8	1.2	1.7	2.4	3.2
Mean - RCP 6.0	91.9	94.3	96.9	99.9	103.2
Std. Deviation - RCP6.0	0.6	0.9	1.2	1.7	2.2
Mean - RCP8.5	91.7	94.0	96.5	99.3	102.3
Std. Deviation - RCP8.5	0.6	0.9	1.2	1.5	1.7

Table S.5. Results for N_2O $pGWP_{20}$ under different IAM-SSP-RCP scenarios.

**Scenario, $pGWP_{20}$ - N_2O**	**2030**	**2035**	**2040**	**2045**	**2050**
AIM_-_SSP3_-_4.5	280.8	288.7	297.1	302.7	308.7
AIM_-_SSP3_-_6.0	281.1	289.5	298.5	305.8	313.6
AIM_-_SSP3_-_8.5	283.7	293.9	305	316.2	328.4
GCAM4_-SSP4_-_2.6	274.3	276.5	278.7	278.7	278.7
GCAM4_-SSP4_-_4.5	277.1	283	289.2	293.8	298.6
GCAM4_-SSP4_-_6.0	279.5	287.5	296.1	304.3	313
GCAM4_-SSP4_-_8.5	280	289.2	299.2	309	319.7
IMAGE_-_SSP1_-_2.6	272.1	274.9	277.7	278.5	279.3
IMAGE_-_SSP1_-_4.5	275.9	281.4	287.4	292.4	297.8
IMAGE_-_SSP1_-_8.5	278.1	284.9	292.3	299.1	306.3
MESSAGE_-SSP2_-_2.6	273.1	276.2	279.3	279.8	280.4
MESSAGE_-SSP2_-_4.5	276	282.2	288.8	294.5	300.6
MESSAGE_-SSP2_-_6.0	277.6	284.7	292.3	300.1	308.5
MESSAGE_-SSP2_-_8.5	276.6	284	291.9	300.5	309.7
REMIND_-SSP5_-_2.6	276.1	281.6	287.3	290.5	293.8
REMIND_-SSP5_-_4.5	277.1	284.1	291.6	298.2	305.2
REMIND_-SSP5_-_6.0	278.9	287.5	296.6	306.5	317.1
REMIND_-SSP5_-_8.5	281.5	293.1	305.8	321.2	338.3
**Statistics, $pGWP_{20}$ - N_2O**	**2030**	**2035**	**2040**	**2045**	**2050**
Mean - ALL RCPs	277.7	284.6	291.8	298.2	305.0
Std. Deviation - ALL RCPs	3.0	5.4	8.1	11.9	16.1
Mean - RCP 2.6	273.9	277.3	280.7	281.8	283.0
Std.Deviation - RCP2.6	1.7	2.9	4.4	5.8	7.2
Mean - RCP 4.5	277.4	283.9	290.8	296.3	302.2
Std. Deviation - RCP4.5	2.0	2.9	3.8	4.2	4.6
Mean - RCP 6.0	279.3	287.3	295.9	304.2	313.0
Std. Deviation - RCP6.0	1.5	2.0	2.6	2.9	3.5
Mean - RCP8.5	280.0	289.0	298.8	309.1	320.3
Std. Deviation - RCP8.5	2.5	4.1	6.0	8.6	11.8

Table S.6. Results for CH_4 $pGTP_{100}$ under different IAM-SSP-RCP scenarios

Scenario, $pGTP_{100}$ - CH_4	2030	2035	2040	2045	2050
AIM_-_SSP3_-_4.5	5.6	5.7	5.8	6	6.2
AIM_-_SSP3_-_6.0	4.9	5.1	5.2	5.3	5.5
AIM_-_SSP3_-_8.5	4.7	4.8	4.9	5	5.2
GCAM4_-SSP4_-_2.6	5.5	5.6	5.7	5.7	5.8
GCAM4_-SSP4_-_4.5	5	5.1	5.2	5.3	5.4
GCAM4_-SSP4_-_6.0	4.7	4.8	4.9	5	5.1
GCAM4_-SSP4_-_8.5	4.1	4.1	4.2	4.3	4.4
IMAGE_-_SSP1_-_2.6	5.8	6	6.2	6.4	6.6
IMAGE_-_SSP1_-_4.5	5	5.2	5.4	5.6	5.8
IMAGE_-_SSP1_-_8.5	4.8	4.9	5	5.2	5.3
MESSAGE_-SSP2_-_2.6	5.6	5.8	5.9	6.1	6.2
MESSAGE_-SSP2_-_4.5	5	5.1	5.3	5.5	5.6
MESSAGE_-SSP2_-_6.0	4.8	4.9	5	5.2	5.3
MESSAGE_-SSP2_-_8.5	4.1	4.2	4.3	4.5	4.6
REMIND_-SSP5_-_2.6	5.6	5.8	6	6.3	6.5
REMIND_-SSP5_-_4.5	5	5.1	5.3	5.5	5.7
REMIND_-SSP5_-_6.0	4.7	4.9	5	5.2	5.4
REMIND_-SSP5_-_8.5	4.1	4.2	4.3	4.5	4.6
**Statistics, $pGTP_{100}$ - CH_4**	**2030**	**2035**	**2040**	**2045**	**2050**
Mean - ALL RCPs	4.9	5.0	5.2	5.3	5.5
Std. Deviation - ALL RCPs	0.5	0.6	0.6	0.6	0.6
Mean - RCP 2.6	5.6	5.8	5.9	6.1	6.3
Std.Deviation - RCP2.6	0.1	0.2	0.2	0.3	0.4
Mean - RCP 4.5	5.1	5.2	5.4	5.6	5.7
Std. Deviation - RCP4.5	0.3	0.3	0.2	0.3	0.3
Mean - RCP 6.0	4.8	4.9	5.0	5.2	5.3
Std. Deviation - RCP6.0	0.1	0.1	0.1	0.1	0.2
Mean - RCP8.5	4.3	4.4	4.5	4.7	4.8
Std. Deviation - RCP8.5	0.3	0.3	0.3	0.3	0.4

Table S.7. Results for N_2O $pGTP_{100}$ under different IAM-SSP-RCP scenarios

**Scenario, $pGTP_{100}$ - N_2O**	**2030**	**2035**	**2040**	**2045**	**2050**
AIM_-_SSP3_-_4.5	232.5	238.2	244.4	248.3	252.5
AIM_-_SSP3_-_6.0	206.8	212.2	218	222.7	227.7
AIM_-_SSP3_-_8.5	197.8	204.2	211	217.9	225.5
GCAM4_-SSP4_-_2.6	227.6	229	230.5	230.4	230.2
GCAM4_-SSP4_-_4.5	204	207.8	211.7	214.7	217.7
GCAM4_-SSP4_-_6.0	195.2	200.1	205.4	210.4	215.7
GCAM4_-SSP4_-_8.5	170.6	175.6	181	186.3	192
IMAGE_-_SSP1_-_2.6	225.7	227.5	229.3	229.6	230
IMAGE_-_SSP1_-_4.5	203.1	206.6	210.3	213.5	216.9
IMAGE_-_SSP1_-_8.5	194.2	198.4	202.9	207	211.4
MESSAGE_-SSP2_-_2.6	226.6	228.6	230.7	230.9	231
MESSAGE_-SSP2_-_4.5	203.2	207.2	211.3	214.9	218.8
MESSAGE_-SSP2_-_6.0	193.9	198.2	202.9	207.6	212.7
MESSAGE_-SSP2_-_8.5	168.6	172.6	176.8	181.4	186.3
REMIND_-SSP5_-_2.6	228.9	232.7	236.7	238.8	241
REMIND_-SSP5_-_4.5	204	208.5	213.3	217.4	221.9
REMIND_-SSP5_-_6.0	194.8	200	205.6	211.7	218.2
REMIND_-SSP5_-_8.5	171.4	177.7	184.6	192.9	202.2
**Statistics, $pGTP_{100}$ - N_2O**	**2030**	**2035**	**2040**	**2045**	**2050**
Mean - ALL RCPs	201.8	206.1	210.7	214.7	219.0
Std. Deviation - ALL RCPs	20.0	19.3	18.6	17.2	15.9
Mean - RCP 2.6	227.2	229.4	231.8	232.4	233.0
Std.Deviation - RCP2.6	1.4	2.3	3.3	4.3	5.3
Mean - RCP 4.5	209.1	213.3	217.8	221.4	225.2
Std. Deviation - RCP4.5	12.9	13.7	14.7	14.9	15.2
Mean - RCP 6.0	197.6	202.5	207.9	213.0	218.5
Std. Deviation - RCP6.0	6.1	6.4	6.8	6.6	6.5
Mean - RCP8.5	180.1	185.3	190.8	196.6	203.0
Std. Deviation - RCP8.5	12.7	13.0	13.3	13.5	14.0

Table S.8. Results for CH_4 $pGTP_{50}$ under different IAM-SSP-RCP scenarios

**Scenario, $pGTP_{50}$ - CH_4**	**2030**	**2035**	**2040**	**2045**	**2050**
AIM_-_SSP3_-_4.5	10.5	10.7	11	11.4	11.7
AIM_-_SSP3_-_6.0	10.3	10.6	10.8	11.2	11.6
AIM_-_SSP3_-_8.5	10.5	10.8	11	11.3	11.6
GCAM4_-SSP4_-_2.6	10.5	10.6	10.7	10.8	10.9
GCAM4_-SSP4_-_4.5	10.4	10.6	10.8	11	11.2
GCAM4_-SSP4_-_6.0	10.5	10.8	11	11.2	11.5
GCAM4_-SSP4_-_8.5	9.7	9.8	10	10.3	10.5
IMAGE_-_SSP1_-_2.6	10.9	11.3	11.7	12	12.4
IMAGE_-_SSP1_-_4.5	10.6	10.9	11.3	11.7	12.1
IMAGE_-_SSP1_-_8.5	10.7	11	11.3	11.6	11.9
MESSAGE_-SSP2_-_2.6	10.6	10.9	11.2	11.4	11.7
MESSAGE_-SSP2_-_4.5	10.5	10.8	11.1	11.5	11.8
MESSAGE_-SSP2_-_6.0	10.7	10.9	11.2	11.6	12
MESSAGE_-SSP2_-_8.5	9.8	10	10.3	10.6	11
REMIND_-SSP5_-_2.6	10.6	11	11.4	11.8	12.3
REMIND_-SSP5_-_4.5	10.4	10.8	11.1	11.5	11.9
REMIND_-SSP5_-_6.0	10.6	10.9	11.2	11.6	12
REMIND_-SSP5_-_8.5	9.8	10	10.3	10.6	10.9
**Statistics, $pGTP_{50}$ - CH_4**	**2030**	**2035**	**2040**	**2045**	**2050**
Mean - ALL RCPs	10.4	10.7	11.0	11.3	11.6
Std. Deviation - ALL RCPs	0.3	0.4	0.4	0.5	0.5
Mean - RCP 2.6	10.6	10.9	11.2	11.5	11.8
Std.Deviation - RCP2.6	0.2	0.3	0.4	0.5	0.7
Mean - RCP 4.5	10.5	10.8	11.1	11.4	11.7
Std. Deviation - RCP4.5	0.1	0.1	0.2	0.3	0.3
Mean - RCP 6.0	10.5	10.8	11.0	11.4	11.8
Std. Deviation - RCP6.0	0.2	0.1	0.2	0.2	0.3
Mean - RCP8.5	10.1	10.3	10.6	10.9	11.2
Std. Deviation - RCP8.5	0.4	0.5	0.5	0.5	0.5

Table S.9. Results for N_2O $pGTP_{50}$ under different IAM-SSP-RCP scenarios

**Scenario, $pGTP_{50}$ - N_2O**	**2030**	**2035**	**2040**	**2045**	**2050**
AIM_-_SSP3_-_4.5	285.6	293.1	301.1	306.3	311.8
AIM_-_SSP3_-_6.0	281.7	289.5	297.9	304.7	311.9
AIM_-_SSP3_-_8.5	288.4	298.2	308.7	319.3	330.9
GCAM4_-SSP4_-_2.6	279.3	281.3	283.4	283.2	283.1
GCAM4_-SSP4_-_4.5	277.8	283.3	289	293.3	297.7
GCAM4_-SSP4_-_6.0	284.4	292	300.1	307.8	316.1
GCAM4_-SSP4_-_8.5	263.4	271.5	280.3	288.9	298.3
IMAGE_-_SSP1_-_2.6	277.1	279.5	282	282.6	283.2
IMAGE_-_SSP1_-_4.5	276.6	281.7	287.1	291.8	296.7
IMAGE_-_SSP1_-_8.5	282.9	289.4	296.4	302.7	309.5
MESSAGE_-SSP2_-_2.6	278.1	280.9	283.7	284.1	284.4
MESSAGE_-SSP2_-_4.5	276.7	282.4	288.5	293.8	299.4
MESSAGE_-SSP2_-_6.0	282.5	289.2	296.4	303.7	311.6
MESSAGE_-SSP2_-_8.5	260.3	266.8	273.7	281.2	289.2
REMIND_-SSP5_-_2.6	281	286.1	291.4	294.3	297.2
REMIND_-SSP5_-_4.5	277.8	284.3	291.2	297.3	303.8
REMIND_-SSP5_-_6.0	283.8	291.9	300.6	309.9	320
REMIND_-SSP5_-_8.5	264.7	274.9	286.1	299.7	314.8
**Statistics, $pGTP_{50}$ - N_2O**	**2030**	**2035**	**2040**	**2045**	**2050**
Mean - ALL RCPs	277.8	284.1	290.8	296.7	303.0
Std. Deviation - ALL RCPs	7.7	8.0	8.9	10.7	13.5
Mean - RCP 2.6	278.9	281.9	285.1	286.0	286.9
Std. Deviation - RCP2.6	1.7	2.9	4.2	5.5	6.8
Mean - RCP 4.5	278.9	284.9	291.3	296.5	301.8
Std. Deviation - RCP4.5	3.8	4.7	5.6	5.8	6.2
Mean - RCP 6.0	283.1	290.6	298.7	306.5	314.9
Std. Deviation - RCP6.0	1.2	1.5	2.0	2.8	4.0
Mean - RCP8.5	271.7	279.9	288.8	298.1	308.2
Std. Deviation - RCP8.5	11.4	11.8	12.3	13.0	14.3

Section 2. Python codes for implementing CFs (table format)

Code #1. Loading pGWP$_{100}$ characterization factors in Brightway

```
# Loading pGWP100 CFs
# You can adapt this code for pGWP20 by replacing all instances of 'GWP100' with 'GWP20' throughout the code.
# This code was only tested with Brightway2, ecoinvent 3.9 database (and their respective biosphere3 database).

import brightway2 as bw
from brightway2 import *
import pandas as pd

#read BW2 projects
bw.projects

#select your own Brightway project
bw.projects.set_current("XXXXXX")  # Replace XXXXXX with your project name
```

```
# Defining the method to be updated - GWP100
method_key = ('IPCC 2021', 'climate change', 'global warming potential (GWP100)')

# Load the characterization factors
cfs = bw.Method(method_key).load()

# Print all tuples (flow keys and CFs)
for cf in cfs:
    print(cf)
```

```
# Extracting this tuple from the existing method to Excel file
cfs = bw.Method(method_key).load()
# Create a list to store flow details
data = []

for flow_key, cf in cfs:
    db_name, flow_code = flow_key
    flow = bw.Database(db_name).get(flow_code)  # Retrieve flow details
    flow_name = flow['name']  # Get flow name
    data.append([flow_name, flow_code, cf])  # Store in list

# Convert to DataFrame
df = pd.DataFrame(data, columns=['Flow Name', 'Flow Code', 'Characterization Factor'])

# Save to Excel
export_path = "C:/XXXXXXXXXX.../IPCC2021 GWP100.xlsx"  # find the pathway to the folder where you saved this file.
df.to_excel(export_path, index=False)
print(f"Exported with flow names to: {export_path}")
```

```
# Generating multiple Excel files that will be used to create new methods
import pandas as pd
import os
import re
from tqdm import tqdm

# Define file paths
base_path = "C:/XXXXXXXX..." # pathway to the folder where you saved your excel files
output_path = os.path.join(base_path, "GWP100")
original_file = "C:/XXXXXXXX.../IPCC2021 GWP100.xlsx" # Find the pathway to the folder where you saved this file
update_file = os.path.join(base_path, "selected_flows.xlsx")
GWP100_CH4_table = os.path.join(base_path, "GWP100_CH4_table.xlsx")
GWP100_N2O_table = os.path.join(base_path, "GWP100_N2O_table.xlsx")

# Load original data (constant for all iterations)
df_original = pd.read_excel(original_file)
df_updates = pd.read_excel(update_file)
df_CH4 = pd.read_excel(GWP100_CH4_table)
df_N2O = pd.read_excel(GWP100_N2O_table)

# Extract IAM scenarios and pulse years from GWP tables
scenarios = df_CH4.iloc[:, 0].astype(str).tolist()
years = df_CH4.columns[1:].tolist()

# Ensure output directory exists
os.makedirs(output_path, exist_ok=True)

def normalize_scenario_name(name):
    return re.sub(r'[^a-zA-Z0-9]', '', name).lower()

# Normalize IAM scenario names for matching
df_CH4["Normalized Scenario"] = df_CH4.iloc[:, 0].apply(normalize_scenario_name)
df_N2O["Normalized Scenario"] = df_N2O.iloc[:, 0].apply(normalize_scenario_name)

# Create a progress bar
total_iterations = len(scenarios) * len(years)
with tqdm(total=total_iterations, desc="Processing IAM Scenarios", unit="file") as pbar:
    # Iterate through all scenarios and years
    for scenario in scenarios:
        for year in years:
            norm_scenario = normalize_scenario_name(scenario)

            # Get methane and N2O values for the given scenario and year
            ch4_value = df_CH4.loc[df_CH4["Normalized Scenario"] == norm_scenario, year].values
            n2o_value = df_N2O.loc[df_N2O["Normalized Scenario"] == norm_scenario, year].values

            if ch4_value.size == 0 or n2o_value.size == 0:
                pbar.update(1)
                continue # Skip if no corresponding values found
```

```
        ch4_value = ch4_value[0]
        n2o_value = n2o_value[0]

        # Create a copy of the original data
        df_updated = df_original.copy()

        # Update characterization factors based on Flow Code
        methane_flows = df_updates[df_updates['Flow Name'].str.contains("methane", case=False,
na=False)]['Flow Code']
        n2o_flows = df_updates[df_updates['Flow Name'].str.contains("dinitrogen monoxide", case=False,
na=False)]['Flow Code']

        df_updated.loc[df_updated['Flow Code'].isin(methane_flows), 'Characterization Factor'] = ch4_value
        df_updated.loc[df_updated['Flow Code'].isin(n2o_flows), 'Characterization Factor'] = n2o_value

        # Define output file name
        output_filename = f"IPCC_2021_GWP100_{scenario}_{year}.xlsx"
        output_filepath = os.path.join(output_path, output_filename)

        # Save the updated file
        df_updated.to_excel(output_filepath, index=False)

        # Update progress bar
        pbar.update(1)
```

```
#Loading new methods based on IPCC2021, updating CH4 and N2O according to prospective GWP100

import brightway2 as bw
import pandas as pd
import os
from tqdm import tqdm

# Select Brightway project
bw.projects.set_current("XXXXXXXXX")  # Find your BW2 project name

# Define input directory for creating methods for GWP100 (you also need to create this folder)
input_dir = "C:/XXXXXXX.../GWP100"  # insert the pathway to the folder for your GWP100 CFs

# Ensure input directory exists
if not os.path.isdir(input_dir):
   raise FileNotFoundError(f"Input directory '{input_dir}' does not exist.")

# Get list of Excel files to process
excel_files = [f for f in os.listdir(input_dir) if f.endswith(".xlsx")]

if not excel_files:
   raise ValueError("No Excel files found in the specified directory.")

# Load biosphere database into a dictionary for faster lookup
```

```
biosphere_db = bw.Database('biosphere3')
biosphere_dict = {act['code']: act for act in biosphere_db}

# Initialize progress bar
with tqdm(total=len(excel_files), desc="Processing LCIA Methods", unit="file") as pbar:
    for file in excel_files:
        file_path = os.path.join(input_dir, file)

        try:
            df_updated = pd.read_excel(file_path)
        except Exception as e:
            print(f"Error reading {file}: {e}")
            pbar.update(1)
            continue

        # Ensure required columns exist
        required_columns = {'Flow Code', 'Characterization Factor'}
        if not required_columns.issubset(df_updated.columns):
            print(f"Skipping {file}: Missing required columns {required_columns}.")
            pbar.update(1)
            continue

        # Extract IAM scenario and Pulse Year from filename
        parts = file.replace("IPCC_2021_", "").replace(".xlsx", "").split("_")

        # Ensure filename parsing is correct
        if len(parts) < 2:
            print(f"Skipping {file}: Unable to extract scenario and year.")
            pbar.update(1)
            continue

        scenario = "_".join(parts[:-1]).strip()  # Extract scenario name
        year = parts[-1].strip()  # Extract pulse year

        # Ensure scenario and year are not empty
        if not scenario or not year:
            print(f"Skipping {file}: Invalid scenario ({scenario}) or year ({year}).")
            pbar.update(1)
            continue

        # Define the new method key
        new_method_key = ("IPCC2021_GWP100", scenario, year)

        # Register the new method if it doesn’t exist
        try:
            if new_method_key not in bw.methods:
                bw.Method(new_method_key).register()
        except Exception as e:
            print(f"Error registering method {new_method_key}: {e}")
            pbar.update(1)
            continue
```

```
        # Prepare the list of CFs
        cf_list = []
        missing_flows = 0

        for _, row in df_updated.iterrows():
            flow_code = row['Flow Code']
            new_cf = row['Characterization Factor']

            # Ensure CF is a valid number
            if not isinstance(new_cf, (int, float)):
                continue

            # Find the flow in the biosphere3 database
            flow = biosphere_dict.get(flow_code)
            if flow:
                cf_list.append((flow.key, new_cf))
            else:
                missing_flows += 1

        # Write the new method with updated CFs
        if cf_list:
            try:
                bw.Method(new_method_key).write(cf_list)
            except Exception as e:
                print(f"Error writing method {new_method_key}: {e}")

        if missing_flows > 0:
            print(f"Warning: {missing_flows} flows not found in biosphere3 for {file}.")

        # Update progress bar
        pbar.update(1)

print("All files processed successfully!")
```

Code #2. Loading $pGTP_{100}$ characterization factors

```
# Code for loading pGTP100 CFs
# This code was only tested in Brightway2, with ecoinvent 3.9 database (and its respective biosphere3).
# You can adapt this code for pGTP50 by replacing all instances of 'GTP100' with 'GTP50' throughout the code.

import brightway2 as bw
from brightway2 import *
import pandas as pd

#read BW2 projects
bw.projects

#select one existing BW project
bw.projects.set_current("XXXXXX")  # Replace with your BW2 project name
```

```
# Define the method to be updated – GTP100
method_key = ('IPCC 2021', 'climate change', 'global temperature change potential (GTP100)')

# Load the characterization factors
cfs = bw.Method(method_key).load()

# Print all tuples (flow keys and CFs)
for cf in cfs:
    print(cf)
```

```
# Extracting this tuple from selected EXISTING method to Excel file
cfs = bw.Method(method_key).load()

# Create a list to store flow details
data = []

for flow_key, cf in cfs:
    db_name, flow_code = flow_key
    flow = bw.Database(db_name).get(flow_code)  # Retrieve flow details
    flow_name = flow['name']  # Get flow name
    data.append([flow_name, flow_code, cf])  # Store in list

# Convert to DataFrame
df = pd.DataFrame(data, columns=['Flow Name', 'Flow Code', 'Characterization Factor'])

# Save to Excel
export_path = "C:/XXXXXXXXX.../IPCC2021 GTP100.xlsx" # Find the pathway to the folder where you saved this file

df.to_excel(export_path, index=False)

print(f"Exported with flow names to: {export_path}")
```

```
# Creating multiple  Excel files that will  be used to create new methods

import  pandas as pd
import  os
import  re
from tqdm import  tqdm

# Define file paths
base_path = "C:/XXXXXXXXX"  # pathway  to the folder where you saved your excel files output_path =
os.path.join(base_path, "GTP100")
original_file  = "C:/XXXXXX.../IPCC2021  GTP100.xlsx" # Find the pathway to the folder where you saved
this file
update_file = os.path.join(base_path, "selected_flows.xlsx")
GTP100_CH4_table = os.path.join(base_path,  "GTP100_CH4_table.xlsx")
GTP100_N2O_table = os.path.join(base_path, "GTP100_N2O_table.xlsx")

# Load original  data (constant for all iterations)
df_original  = pd.read_excel(original_file)
df_updates = pd.read_excel(update_file)
df_CH4 = pd.read_excel(GTP100_CH4_table)
df_N2O = pd.read_excel(GTP100_N2O_table)

# Extract IAM scenarios and pulse years from GTP tables
scenarios = df_CH4.iloc[:, 0].astype(str).tolist()
years = df_CH4.columns[1:].tolist()

# Ensure output  directory exists
os.makedirs(output_path,  exist_ok=True)

def normalize_scenario_name(name):
    return re.sub(r'[^a-zA-Z0-9]', '', name).lower()

# Normalize  IAM scenario names for matching
df_CH4["Normalized  Scenario"] = df_CH4.iloc[:, 0].apply(normalize_scenario_name)
df_N2O["Normalized  Scenario"] = df_N2O.iloc[:, 0].apply(normalize_scenario_name)

# Create a progress bar
total_iterations  = len(scenarios) * len(years)
with tqdm(total=total_iterations,  desc="Processing IAM Scenarios",  unit="file")  as pbar:
    # Iterate through all scenarios and years
    for scenario in scenarios:
        for year in years:
            norm_scenario = normalize_scenario_name(scenario)

            # Get methane and N2O values for the given scenario and year
            ch4_value = df_CH4.loc[df_CH4["Normalized  Scenario"] == norm_scenario, year].values
            n2o_value = df_N2O.loc[df_N2O["Normalized  Scenario"] == norm_scenario, year].values

            if ch4_value.size == 0 or n2o_value.size == 0:
                pbar.update(1)
                continue  # Skip if no corresponding  values found
```

```
        ch4_value = ch4_value[0]
        n2o_value = n2o_value[0]

        # Create a copy of the original data
        df_updated = df_original.copy()

        # Update characterization factors based on Flow Code
        methane_flows = df_updates[df_updates['Flow Name'].str.contains("methane", case=False,
na=False)]['Flow Code']
        n2o_flows = df_updates[df_updates['Flow Name'].str.contains("dinitrogen monoxide", case=False,
na=False)]['Flow Code']

        df_updated.loc[df_updated['Flow Code'].isin(methane_flows), 'Characterization Factor'] = ch4_value
        df_updated.loc[df_updated['Flow Code'].isin(n2o_flows), 'Characterization Factor'] = n2o_value

        # Define output file name
        output_filename = f"IPCC_2021_GTP100_{scenario}_{year}.xlsx"
        output_filepath = os.path.join(output_path, output_filename)

        # Save the updated file
        df_updated.to_excel(output_filepath, index=False)

        # Update progress bar
        pbar.update(1)
```

```
#Loading new methods based on IPCC2021, updating CH4 and N2O according to prospective GTP100

import brightway2 as bw
import pandas as pd
import os
from tqdm import tqdm

# Select Brightway2 project
bw.projects.set_current("XXXXXXXX")   # Insert your BW2 project name here

# Define input directory for creating methods for GTP100 (you also need to create this folder)
input_dir = "C:/XXXXXXXXX.../GTP100"  # insert the pathway to the folder for your GTP100 CFs


# Ensure input directory exists
if not os.path.isdir(input_dir):
    raise FileNotFoundError(f"Input directory '{input_dir}' does not exist.")

# Get list of Excel files to process
excel_files = [f for f in os.listdir(input_dir) if f.endswith(".xlsx")]

if not excel_files:
    raise ValueError("No Excel files found in the specified directory.")
```

```
# Load biosphere database into a dictionary for faster lookup
biosphere_db = bw.Database('biosphere3')
biosphere_dict = {act['code']: act for act in biosphere_db}

# Initialize progress bar
with tqdm(total=len(excel_files), desc="Processing LCIA Methods", unit="file") as pbar:
    for file in excel_files:
        file_path = os.path.join(input_dir, file)

        try:
            df_updated = pd.read_excel(file_path)
        except Exception as e:
            print(f"Error reading {file}: {e}")
            pbar.update(1)
            continue

        # Ensure required columns exist
        required_columns = {'Flow Code', 'Characterization Factor'}
        if not required_columns.issubset(df_updated.columns):
            print(f"Skipping {file}: Missing required columns {required_columns}.")
            pbar.update(1)
            continue

        # Extract IAM scenario and Pulse Year from filename
        parts = file.replace("IPCC_2021_GTP100", "").replace(".xlsx", "").split("_")

        if len(parts) < 2:
            print(f"Skipping {file}: Unable to extract scenario and year.")
            pbar.update(1)
            continue

        scenario = "_".join(parts[:-1]).strip()
        year = parts[-1].strip()

        if not scenario or not year:
            print(f"Skipping {file}: Invalid scenario ({scenario}) or year ({year}).")
            pbar.update(1)
            continue

        # Define the new method key
        new_method_key = ("IPCC2021_GTP100", scenario, year)

        # Register the new method if it doesn't exist
        try:
            if new_method_key not in bw.methods:
                bw.Method(new_method_key).register()
        except Exception as e:
            print(f"Error registering method {new_method_key}: {e}")
            pbar.update(1)
            continue
```

```
    # Prepare the list of CFs
    cf_list = []
    missing_flows = 0

    for _, row in df_updated.iterrows():
        flow_code = row['Flow Code']
        new_cf = row['Characterization Factor']

        if not isinstance(new_cf, (int, float)):
            continue

        # Lookup in biosphere dict (faster than repeated database calls)
        flow = biosphere_dict.get(flow_code)
        if flow:
            cf_list.append((flow.key, new_cf))
        else:
            missing_flows += 1

    # Write the new method with updated CFs
    if cf_list:
        try:
            bw.Method(new_method_key).write(cf_list)
        except Exception as e:
            print(f"Error writing method {new_method_key}: {e}")

    if missing_flows > 0:
        print(f"Warning: {missing_flows} flows not found in biosphere3 for {file}.")

    # Update progress bar
    pbar.update(1)

print("All files processed successfully!")
```

Section 3. GWP & GTP – Unified Folder Structure and Guidelines for using AGWP and AGTP codes

This part summarizes the unified folder structure and setup instructions for running the AGWP (Absolute Global Warming Potential) and AGTP (Absolute Global Temperature Potential) tools for CO_2, CH_4, and N_2O using multiple IAM-SSP-RCP scenarios. The last codes provided allow the calculation of:

- GWP metrics: GWP20 and GWP100 for CH_4 and N_2O for pulse years 2030–2100.
- GTP metrics: GTP50 and GTP100 for CH_4 and N_2O for pulse years 2030–2100.

Additionally, the codes automatically generate Excel tables synthesizing IAM-based prospective values for GWP100, GWP20, GTP50, and GTP100 for both CH_4 and N_2O as final outputs.

Unified Folder Structure

Obs: Download the Excel input files (IRF, RE) to the same folder where you are saving the Python scripts!

```
your_folder/
│
├── inputs/
│   │
│   ├── IRF/                    # Impulse Response Function files
│   │   ├── pIRF_CO2.xlsx
│   │   ├── pIRF_CH4.xlsx
│   │   └── pIRF_N2O.xlsx
│   │
│   ├── RE_CO2/                 # Radiative Efficiency for CO₂ per IAM
│   │   ├── pRE_CO2_IMAGE.xlsx
│   │   ├── pRE_CO2_REMIND.xlsx
│   │   ├── pRE_CO2_MESSAGE.xlsx
│   │   ├── pRE_CO2_GCAM4.xlsx
│   │   └── pRE_CO2_AIM.xlsx
│   │
│   ├── RE_CH4/                 # Radiative Efficiency for CH₄ per IAM
│   │   ├── pRE_CH4_IMAGE.xlsx
│   │   ├── pRE_CH4_REMIND.xlsx
│   │   ├── pRE_CH4_MESSAGE.xlsx
│   │   ├── pRE_CH4_GCAM4.xlsx
│   │   └── pRE_CH4_AIM.xlsx
│   │
│   └── RE_N2O/                 # Radiative Efficiency for N₂O per IAM
│       ├── pRE_N2O_IMAGE.xlsx
│       ├── pRE_N2O_REMIND.xlsx
│
```

```
│     ├── pRE_N2O_MESSAGE.xlsx
│     ├── pRE_N2O_GCAM4.xlsx
│     └── pRE_N2O_AIM.xlsx
│
├── scripts/                    # Python scripts for each gas
│   ├── AGWP_CO2.py  (Code #3)
│   ├── AGWP_CH4.py  (Code #4)
│   ├── AGWP_N2O.py  (Code #5)
│   ├── GWP.py       (Code #6)
│   ├── AGTP_CO2.py  (Code #7)
│   ├── AGTP_CH4.py  (Code #8)
│   ├── AGTP_N2O.py  (Code #9)
│   └── GTP.py       (Code #10)
│
└── outputs/
    ├── GWP/
    │   ├── CO2/                # AGWP results and plots for CO₂
    │   ├── CH4/                # AGWP results and plots for CH₄
    │   └── N2O/                # AGWP results and plots for N₂O
    └── GTP/
        ├── CO2/                # AGTP results and plots for CO₂
        ├── CH4/                # AGTP results and plots for CH₄
        └── N2O/                # AGTP results and plots for N₂O
```

Folder Description

Folder	Purpose
inputs/IRF/	Stores Impulse Response Function (IRF) Excel files for all gases.
inputs/RE_CO2/, RE_CH4/, RE_N2O/	Contain Radiative Efficiency (RE) data per IAM (IMAGE, REMIND, MESSAGE, GCAM4, AIM).
scripts/	Contains Python computation codes for each gas (AGWP & AGTP), as well as combined GWP.py and GTP.py scripts.
outputs/GWP/	Automatically stores AGWP Excel outputs and plots. Subfolders per gas keep results organized.
outputs/GTP/	Automatically stores AGTP Excel outputs and plots. Subfolders per gas keep results organized.
**Excel synthesis tables**	Final output tables summarizing IAM-based prospective values for **GWP100, GWP20, GTP50, and GTP100** for CH_4 and N_2O.

Combined Workflow Summary (GWP & GTP)

1. Prepare Inputs:
   Place all Excel input files in their corresponding folders inside inputs/:

- IRF/ → Impulse Response Functions
- RE_CO2/, RE_CH4/, RE_N2O/ → Radiative Efficiency per IAM

2. Run AGWP and AGTP Scripts:

```
# AGWP Calculations
python scripts/AGWP_CO2.py (Code #3)
python scripts/AGWP_CH4.py (Code #4)
python scripts/AGWP_N2O.py (Code #5)

# AGTP Calculations
python scripts/AGTP_CO2.py (Code #7)
python scripts/AGTP_CH4.py (Code #8)
python scripts/AGTP_N2O.py (Code #9)
```

3. Outputs:

   - AGWP results → outputs/GWP/[CO2|CH4|N2O]/
   - AGTP results → outputs/GTP/[CO2|CH4|N2O]/

4. GWP and GTP Tables (2030-2100):

   - For GWP Excel Tables: Generate CH_4 and N_2O pGWP20 and pGWP100 (pulse years 2030–2100) >> Use Code #6
   - For GTP Excel Tables: Generate CH_4 and N_2O pGTP50 and pGTP100 (pulse years 2030–2100)>> Use Code#10

5. Implementing GWP and GTP tables:

   After generating Excel synthesis tables for 2030-2100, implement these yearly CFs in Brightway (using codes #1 and #2).

Code #3. CO2 AGWP for multiple IAM-SSP-RCP scenarios

```
# AGWP_CO2 — PUBLIC RELEASE VERSION (November 2025)
# Author: [Watanabe and Cherubini (2025)]
# Description:
#   Computes Absolute Global Warming Potential (AGWP) for CO2
#   using IAM-based RE and IRF data.
#   Users must provide Excel input files in the /inputs folder
#   and create an /outputs/GWP folder for results.

import os
import pandas as pd
import numpy as np
import matplotlib.pyplot as plt
import seaborn as sns
from tqdm import tqdm
import matplotlib.ticker as ticker

# ------------------------------------------------------------------------
# GLOBAL SETTINGS
# ------------------------------------------------------------------------
plt.rcParams["font.family"] = "DejaVu Sans"

# Define color palette
full_palette = sns.color_palette("viridis", 256)
palette = [full_palette[i] for i in [0, 60, 130, 255]]
scenario_colors = {'2.6': palette[0], '4.5': palette[1], '6.0': palette[2], '8.5': palette[3]}

def get_scenario_color(scenario_name):
    for key, color in scenario_colors.items():
        if scenario_name.endswith(key):
            return color
    return '#7f7f7f'  # default gray

# ------------------------------------------------------------------------
# DATA LOADING FUNCTIONS
# ------------------------------------------------------------------------
def load_re_data(file_path):
    """Load Radiative Efficiency (RE) data from Excel."""
    try:
        df_re = pd.read_excel(file_path, sheet_name="Sheet1")
    except FileNotFoundError:
        print(f"Error: RE file not found at {file_path}")
        return None, None, None
    re_years = df_re.iloc[:, 0].astype(int).tolist()
    scenario_names = df_re.columns[1:].tolist()
    re_data = df_re.iloc[:, 1:].values.tolist()
    return re_years, scenario_names, re_data

def load_irf_data(file_path, expected_scenarios):
    """Load Impulse Response Function (IRF) data from Excel."""
    try:
        df_irf = pd.read_excel(file_path, sheet_name="Sheet1")
    except FileNotFoundError:
        print(f"Error: IRF file not found at {file_path}")
```

```
        return None, None
    irf_years = df_irf.iloc[:, 0].astype(int).tolist()
    df_irf = df_irf.iloc[:, :len(expected_scenarios) + 1]
    irf_data = df_irf.iloc[:, 1:].values.tolist()
    return irf_years, irf_data

# ------------------------------------------------------------------------
# COMPUTATION FUNCTIONS
# ------------------------------------------------------------------------
def calculate_rf(pulse_year, re_years, re_values, irf_values, integration_years=125):
    """Compute instantaneous radiative forcing (RF) for a given pulse year."""
    try:
        pulse_year_index = re_years.index(pulse_year)
    except ValueError:
        print(f"Error: Pulse Year {pulse_year} not found in RE data.")
        return None
    re_val = re_values[pulse_year_index]
    rf_values = [
        re_val * irf_values[i] * 1e9 * 1e-6 * 28.97 / 44.01 / 5.13252
        for i in range(min(integration_years, len(irf_values)))
    ]
    return rf_values

def calculate_agwp(rf_values):
    """Integrate radiative forcing (RF) to obtain AGWP over time."""
    agwp_values = [(rf_values[0] + rf_values[1]) / 2]
    for t in range(1, len(rf_values) - 1):
        agwp_t = agwp_values[t - 1] + (rf_values[t] + rf_values[t + 1]) / 2
        agwp_values.append(agwp_t)
    agwp_values.append(agwp_values[-1] + rf_values[-1])
    return agwp_values

def create_agwp_table(pulse_year, re_years, agwp_values):
    pulse_index = re_years.index(pulse_year)
    table_years = [pulse_year + i for i in range(len(agwp_values))]
    return [[table_years[i], agwp_values[i]] for i in range(len(agwp_values))]

def save_output(file_path, data, headers):
    os.makedirs(os.path.dirname(file_path), exist_ok=True)
    df = pd.DataFrame(data, columns=headers)
    df.to_excel(file_path, index=False)

# ------------------------------------------------------------------------
# MAIN PROCESSING
# ------------------------------------------------------------------------
def process_iam(iam_name, input_folder, output_folder):
    print(f"\nProcessing IAM: {iam_name}")

    re_file_path = os.path.join(input_folder, f"pRE_CO2_{iam_name}.xlsx")
    irf_file_path = os.path.join(input_folder, "pIRF_CO2.xlsx")

    re_years, scenario_names, re_data = load_re_data(re_file_path)
    if re_years is None:
        return
    _, irf_data = load_irf_data(irf_file_path, scenario_names)
    if irf_data is None:
```

```
        return

    pulse_years = list(range(2030, 2101))
    total_iters = len(scenario_names) * len(pulse_years)

    with tqdm(total=total_iters, desc=f"{iam_name}", unit="iteration") as pbar:
        for scenario_idx, scenario_name in enumerate(scenario_names):
            for pulse_year in pulse_years:
                re_values = [row[scenario_idx] for row in re_data]
                irf_values = [row[scenario_idx] for row in irf_data]

                rf_values = calculate_rf(pulse_year, re_years, re_values, irf_values, integration_years=125)
                if rf_values is None:
                    pbar.update(1)
                    continue

                agwp_values = calculate_agwp(rf_values)
                agwp_table = create_agwp_table(pulse_year, re_years, agwp_values)

                scenario_safe = scenario_name.replace(" ", "_")
                out_file = os.path.join(output_folder, f"output_AGWP_CO2_{scenario_safe}_Pulse_{pulse_year}.xlsx")
                save_output(out_file, agwp_table, ['Calendar Year', 'AGWP_CO2'])

                pbar.update(1)

# ---------------------------------------------------------------------------
# PLOTTING
# ---------------------------------------------------------------------------
def plot_agwp(iam_name, scenario_names, pulse_years, output_folder):
    fig, ax = plt.subplots(figsize=(19, 9))
    sns.set(style="whitegrid")
    plotted_labels = set()
    max_y_value = 0

    for scenario_name in scenario_names:
        scenario_safe = scenario_name.replace(" ", "_")
        color = get_scenario_color(scenario_name)

        for pulse_year in pulse_years:
            file_path = os.path.join(output_folder, f"output_AGWP_CO2_{scenario_safe}_Pulse_{pulse_year}.xlsx")
            if not os.path.exists(file_path):
                continue
            df = pd.read_excel(file_path)
            max_y_value = max(max_y_value, df['AGWP_CO2'].max())

            if scenario_name not in plotted_labels:
                ax.plot(df['Calendar Year'], df['AGWP_CO2'], label=scenario_name, color=color, linewidth=3)
                plotted_labels.add(scenario_name)
            else:
                ax.plot(df['Calendar Year'], df['AGWP_CO2'], color=color, alpha=0.4, linewidth=2)

    ax.set_xlabel("Calendar Year", fontsize=20)
    ax.set_ylabel("AGWP CO2", fontsize=20)
    ax.tick_params(axis='both', which='major', labelsize=16)

    ax.set_xlim(2025, 2155)
```

```
    ax.xaxis.set_minor_locator(ticker.MultipleLocator(5))

    y_max = max_y_value * 1.1 if max_y_value > 0 else 1
    ax.set_ylim(0, y_max)
    ax.yaxis.set_minor_locator(ticker.MultipleLocator(y_max / 10))
    ax.grid(which='both', linestyle='--', linewidth=0.5, alpha=0.7)
    ax.legend(fontsize=14)
    plt.tight_layout()
    plt.show()

# ---------------------------------------------------------------------------
# MAIN ENTRY POINT
# ---------------------------------------------------------------------------
def main():
    input_folder = "inputs"
    output_folder = os.path.join("outputs", "GWP")

    iams = ["IMAGE", "REMIND", "MESSAGE", "GCAM4", "AIM"]
    pulse_years = list(range(2030, 2101))  # Extended to 2100

    for iam in iams:
        process_iam(iam, input_folder, output_folder)
        re_file_path = os.path.join(input_folder, f"pRE_CO2_{iam}.xlsx")
        re_years, scenario_names, _ = load_re_data(re_file_path)
        if scenario_names:
            plot_agwp(iam, scenario_names, pulse_years, output_folder)

if __name__ == "__main__":
    main()
```

Code #4 CH_4 AGWP for multiple IAM-SSP-RCP scenarios

```
# AGWP_CH4— PUBLIC RELEASE VERSION (November 2025)
# Author: [Watanabe and Cherubini (2025)]
# Description:
#   Computes Absolute Global Warming Potential (AGWP) for CH4
#   using IAM-based RE and IRF data.
#   Users must provide Excel input files in the /inputs folder
#   and create an /outputs/GWP folder for results.

import pandas as pd
import numpy as np
import os
import matplotlib.pyplot as plt
import seaborn as sns
import difflib  # To find the closest matching filename

# ==================== FILE PATHS ====================
# Handle environments where __file__ is not defined (Jupyter/Spyder)
if "__file__" in globals():
    BASE_DIR = os.path.dirname(os.path.abspath(__file__))
else:
```

```
    BASE_DIR = os.getcwd() # Use current working directory

INPUT_DIR = os.path.join(BASE_DIR, "inputs")
OUTPUT_DIR = os.path.join(BASE_DIR, "outputs", "GWP")
os.makedirs(OUTPUT_DIR, exist_ok=True)

# ================== DATA LOADING ==================
def load_re_data(file_name):
    file_path = os.path.join(INPUT_DIR, file_name)
    df_re = pd.read_excel(file_path, sheet_name="Sheet1")
    re_years = df_re.iloc[:, 0].astype(int).tolist()
    scenario_names = df_re.columns[1:].tolist()
    re_values = df_re.iloc[:, 1:].values
    return re_years, scenario_names, re_values

def load_irf_data(file_name):
    file_path = os.path.join(INPUT_DIR, file_name)
    df_irf = pd.read_excel(file_path, sheet_name="Sheet1")
    irf_values = df_irf.iloc[:, 1:].values
    return irf_values

# ================== RF CALCULATION ==================
def calculate_rf(pulse_year, re_years, re_values_ch4, re_values_co2, irf_values, scenario_index):
    try:
        pulse_year_index = re_years.index(pulse_year)
    except ValueError:
        print(f"Error: Pulse Year {pulse_year} not found in RE data.")
        return None

    if pulse_year_index >= len(re_years):
        print("Error: Pulse year index out of bounds in RE data.")
        return None

    rf_values, factor2_values, conc_values = [], [], []
    C1_values, C2_values, C3_values = [], [], []
    T_s_values, T_d_values = [], []
    factor1_values = []

    constant = 10**-6 * 28.97 / 16.04 * 1 / 5.13252

    T_s, T_d = [0] * 102, [0] * 102
    C1, C2, C3 = [0] * 102, [0] * 102, [0] * 102
    factor2 = [0] * 102

    re_value_ch4_fixed = re_values_ch4[pulse_year_index, scenario_index]
    re_value_co2_fixed = re_values_co2[pulse_year_index, scenario_index]

    for t in range(1, 101):
        irf_value = irf_values[t - 1, scenario_index]

        factor1 = (re_value_ch4_fixed + 0.00014 + 0.00004) * 10**9 * (irf_value * constant)
        factor1_values.append(factor1)

        T_s[t] = T_s[t - 1] + factor1 / 7.7 + (-1.31) * T_s[t - 1] / 7.7 - 1.03 * (0.88/1.03) * (T_s[t - 1] - T_d[t - 1]) / 7.7
        T_d[t] = T_d[t - 1] + (0.88/1.03) * (T_s[t - 1] - T_d[t - 1]) / 147
```

```
        T_s_values.append(T_s[t])
        T_d_values.append(T_d[t])

        C1[t] = T_s[t] * 10**-9 * 11.06 * 10**9 * 0.6368 + C1[t - 1] * np.exp(-1 / 2.376)
        C1_values.append(C1[t])

        C2[t] = T_s[t] * 10**-9 * 11.06 * 10**9 * 0.3322 + C1[t - 1] * np.exp(-1 / 30.14)
        C3[t] = T_s[t] * 10**-9 * 11.06 * 10**9 * 0.031 + C1[t - 1] * np.exp(-1 / 490.1)

        C2_values.append(C2[t])
        C3_values.append(C3[t])

        factor2[t] = (C1[t] + C2[t] + C3[t]) * 10**-6 * 28.97 / 44.01 * 1 / 5.13252 * re_value_co2_fixed * 10**9
        factor2_values.append(factor2[t])

    # Final time step t=101
    t = 101
    irf_value = irf_values[t - 1, scenario_index]
    factor1 = (re_value_ch4_fixed + 0.00014 + 0.00004) * 10**9 * (irf_value * constant)
    factor1_values.append(factor1)

    T_s[t] = T_s[t - 1] + factor1 / 7.7 + (-1.31) * T_s[t - 1] / 7.7 - 1.03 * (0.88/1.03) * (T_s[t - 1] - T_d[t - 1]) / 7.7
    T_d[t] = T_d[t - 1] + (0.88/1.03) * (T_s[t - 1] - T_d[t - 1]) / 147
    T_s_values.append(T_s[t])
    T_d_values.append(T_d[t])

    C1[t] = T_s[t] * 10**-9 * 11.06 * 10**9 * 0.6368 + C1[t - 1] * np.exp(-1 / 2.376)
    C1_values.append(C1[t])
    C2[t] = T_s[t] * 10**-9 * 11.06 * 10**9 * 0.3322 + C1[t - 1] * np.exp(-1 / 30.14)
    C3[t] = T_s[t] * 10**-9 * 11.06 * 10**9 * 0.031 + C1[t - 1] * np.exp(-1 / 490.1)
    C2_values.append(C2[t])
    C3_values.append(C3[t])

    factor2[t] = (C1[t] + C2[t] + C3[t]) * 10**-6 * 28.97 / 44.01 * 1 / 5.13252 * re_value_co2_fixed * 10**9
    factor2_values.append(factor2[t])

    return C1_values, C2_values, C3_values, T_s_values, T_d_values, factor1_values, factor2_values

# ================== AGWP CALCULATION ==================
def calculate_agwp_ch4(factor1_values, factor2_values):
    AGWP_CH4_values = [0]
    for t in range(1, 101):
        agwp_ch4_t = AGWP_CH4_values[t - 1] + (factor1_values[t - 1] + factor1_values[t]) / 2 + (factor2_values[t - 1] +
factor2_values[t]) / 2
        AGWP_CH4_values.append(agwp_ch4_t)
    return AGWP_CH4_values

# ================== SAVE RESULTS ==================
def save_results(output_dir, pulse_year, scenario_name, AGWP_CH4_values):
    file_name = f"output_AGWP_CH4_{scenario_name}_Pulse_{pulse_year}.xlsx"
    file_path = os.path.join(output_dir, file_name)
    calendar_years = list(range(pulse_year, pulse_year + 100))
    df_output = pd.DataFrame({
        "Year": calendar_years,
        "AGWP_CH4": AGWP_CH4_values[1:]
    })
```

```
    df_output.to_excel(file_path, index=False)

# ================== PLOTTING ==================
full_palette = sns.color_palette("viridis", 256)
palette = [full_palette[i] for i in [0, 60, 130, 255]]
scenario_colors = {'2.6': palette[0], '4.5': palette[1], '6.0': palette[2], '8.5': palette[3]}

def get_scenario_color(column_name):
    for key, color in scenario_colors.items():
        if column_name.endswith(key):
            return color
    return '#7f7f7f'

def find_closest_filename(expected_filename, output_folder):
    try:
        all_files = os.listdir(output_folder)
        matches = difflib.get_close_matches(expected_filename, all_files, n=1, cutoff=0.6)
        return matches[0] if matches else None
    except FileNotFoundError:
        print(f"Warning: Output folder '{output_folder}' not found.")
        return None

def plot_agwp_ch4(iam_name, scenario_names, pulse_years, output_folder):
    fig, ax = plt.subplots(figsize=(14, 9))
    sns.set(style="whitegrid")

    scenario_labels_plotted = set()
    no_data = True

    for scenario_name in scenario_names:
        scenario_safe = scenario_name.replace("_", " ")
        rcp_value = scenario_name.split("-")[-1]
        label = f"RCP {rcp_value}"

        for pulse_year in pulse_years:
            expected_filename = f"output_AGWP_CH4_{scenario_name}_Pulse_{pulse_year}.xlsx"
            actual_filename = find_closest_filename(expected_filename, output_folder)
            if not actual_filename:
                continue

            file_path = os.path.join(output_folder, actual_filename)
            try:
                df = pd.read_excel(file_path)
                if df.empty:
                    continue
            except FileNotFoundError:
                continue

            color = get_scenario_color(rcp_value)
            no_data = False

            if label not in scenario_labels_plotted:
                ax.plot(df['Year'], df['AGWP_CH4'], label=label, color=color)
                scenario_labels_plotted.add(label)
            else:
                ax.plot(df['Year'], df['AGWP_CH4'], color=color, alpha=0.5)
```

```
    if not no_data:
        ax.set_xlabel("Pulse Year", fontsize=36)
        ax.set_ylabel("AGWP CH4", fontsize=36)
        ax.set_title(f"AGWP CH4 for {iam_name} Scenarios", fontsize=36)
        ax.legend(loc='upper left', bbox_to_anchor=(1.05, 1), fontsize=36)
        ax.set_xlim(2025, 2200)
        plt.rcParams["font.family"] = "DejaVu Sans"
        plt.tick_params(axis='both', which='major', labelsize=36)
        plt.tight_layout()
        plt.show()
    else:
        print("No data was found for plotting.")

# ================== MAIN ==================
def main():
    iams = ['IMAGE', 'REMIND', 'MESSAGE', 'GCAM4', 'AIM']

    # Extend pulse years from 2030–2100
    pulse_years = list(range(2030, 2101))  # 2101 because range is exclusive at the end

    # Load scenario names from first IAM
    _, scenario_names, _ = load_re_data(f"pRE_CH4_{iams[0]}.xlsx")

    for iam in iams:
        print(f"Processing IAM: {iam}")

        re_years, scenario_names, re_values_ch4 = load_re_data(f"pRE_CH4_{iam}.xlsx")
        _, _, re_values_co2 = load_re_data(f"pRE_CO2_{iam}.xlsx")
        irf_values = load_irf_data("pIRF_CH4.xlsx")

        for scenario_index, scenario_name in enumerate(scenario_names):
            for pulse_year in pulse_years:
                result = calculate_rf(pulse_year, re_years, re_values_ch4, re_values_co2, irf_values, scenario_index)
                if result is None:
                    continue

                C1_values, C2_values, C3_values, T_s_values, T_d_values, factor1_values, factor2_values = result
                AGWP_CH4_values = calculate_agwp_ch4(factor1_values, factor2_values)

                save_results(OUTPUT_DIR, pulse_year, scenario_name, AGWP_CH4_values)

        plot_agwp_ch4(iam, scenario_names, pulse_years, OUTPUT_DIR)


if __name__ == "__main__":
    main()
```

Code #5 N_2O AGWP for multiple IAM-SSP-RCP scenarios

```
# AGWP_N2O — PUBLIC RELEASE VERSION (November 2025)
# Author: [Watanabe and Cherubini (2025)]
# Description:
#   Computes Absolute Global Warming Potential (AGWP) for N2O
#   using IAM-based RE and IRF data.
#   Users must provide Excel input files in the /inputs folder
#   and create an /outputs/GWP folder for results.


import pandas as pd
import numpy as np
import os
from tqdm import tqdm
import matplotlib.pyplot as plt
import seaborn as sns
import difflib

# === DATA LOADING FUNCTIONS ===

def load_re_data(file_path):
    df_re = pd.read_excel(file_path, sheet_name="Sheet1")
    re_years = df_re.iloc[:, 0].astype(int).tolist()
    scenario_names = df_re.columns[1:].tolist()
    re_values = df_re.iloc[:, 1:].values
    return re_years, scenario_names, re_values

def load_irf_data(file_path):
    df_irf = pd.read_excel(file_path, sheet_name="Sheet1")
    irf_values = df_irf.iloc[:, 1:].values
    return irf_values


# === CORE CALCULATION FUNCTIONS ===

def calculate_rf(pulse_year, re_years, re_values_n2o, re_values_ch4, re_values_co2, irf_values, scenario_index):
    if pulse_year not in re_years:
        return None, None, None, None, None, None, None

    pulse_year_index = re_years.index(pulse_year)

    rf_values, factor2_values = [], []
    C1_values, C2_values, C3_values = [], [], []
    T_s_values, T_d_values = [], []
    factor1_values = []

    constant = 10**-6 * 28.97 / 44.013 * 1 / 5.13252

    T_s, T_d = [0] * 102, [0] * 102
    C1, C2, C3 = [0] * 102, [0] * 102, [0] * 102
    factor2 = [0] * 102

    re_value_fixed = re_values_n2o[pulse_year_index, scenario_index]
    re_value2_fixed = re_values_ch4[pulse_year_index, scenario_index]
```

```
    re_value_co2_fixed = re_values_co2[pulse_year_index, scenario_index]

    for t in range(1, 101):
        irf_value = irf_values[t - 1, scenario_index]

        factor1 = (re_value_fixed + 0.00055 - 1.7 * (re_value2_fixed + 0.00014 + 0.00004)) \
                * 10**9 * (irf_value * constant)
        factor1_values.append(factor1)

        T_s[t] = T_s[t - 1] + factor1 / 7.7 + (-1.31) * T_s[t - 1] / 7.7 \
                - 1.03 * (0.88 / 1.03) * (T_s[t - 1] - T_d[t - 1]) / 7.7
        T_d[t] = T_d[t - 1] + (0.88 / 1.03) * (T_s[t - 1] - T_d[t - 1]) / 147

        T_s_values.append(T_s[t])
        T_d_values.append(T_d[t])

        C1[t] = T_s[t] * 10**-9 * 11.06 * 10**9 * 0.6368 + C1[t - 1] * np.exp(-1 / 2.376)
        C2[t] = T_s[t] * 10**-9 * 11.06 * 10**9 * 0.3322 + C1[t - 1] * np.exp(-1 / 30.14)
        C3[t] = T_s[t] * 10**-9 * 11.06 * 10**9 * 0.031 + C1[t - 1] * np.exp(-1 / 490.1)

        C1_values.append(C1[t])
        C2_values.append(C2[t])
        C3_values.append(C3[t])

        factor2[t] = (C1[t] + C2[t] + C3[t]) * constant * re_value_co2_fixed * 10**9
        factor2_values.append(factor2[t])

    return C1_values, C2_values, C3_values, T_s_values, T_d_values, factor1_values, factor2_values


def calculate_agwp_n2o(factor1_values, factor2_values):
    n_years = len(factor1_values)
    if n_years < 2:
        return []

    cumulative = 0
    AGWP_N2O_values = []

    for t in range(n_years - 1):
        delta = (factor1_values[t] + factor1_values[t + 1]) / 2 + \
              (factor2_values[t] + factor2_values[t + 1]) / 2
        cumulative += delta
        AGWP_N2O_values.append(cumulative)

    AGWP_N2O_values.append(AGWP_N2O_values[-1] + factor1_values[-1] + factor2_values[-1])
    return AGWP_N2O_values[:100]


def save_results(output_dir, pulse_year, scenario_name, AGWP_N2O_values):
    years = list(range(pulse_year, pulse_year + 100))
    df_output = pd.DataFrame({"Year": years, "AGWP_N2O": AGWP_N2O_values})
    os.makedirs(output_dir, exist_ok=True)
    file_name = f"output_AGWP_N2O_{scenario_name}_Pulse_{pulse_year}.xlsx"
    df_output.to_excel(os.path.join(output_dir, file_name), index=False)
```

```
def find_closest_filename(expected_filename, output_folder):
    try:
        all_files = os.listdir(output_folder)
        matches = difflib.get_close_matches(expected_filename, all_files, n=1, cutoff=0.6)
        return matches[0] if matches else None
    except FileNotFoundError:
        return None


# === COLOR SCHEME ===

full_palette = sns.color_palette("viridis", 256)
palette = [full_palette[i] for i in [0, 60, 130, 255]]

scenario_colors = {
    '2.6': palette[0],
    '4.5': palette[1],
    '6.0': palette[2],
    '8.5': palette[3],
}

def get_scenario_color(column_name):
    for key, color in scenario_colors.items():
        if column_name.endswith(key):
            return color
    return '#7f7f7f'


# === PLOTTING ===

def plot_agwp_n2o(iam_name, scenario_names, pulse_years, output_folder):
    plt.rcParams["font.family"] = "DejaVu Sans"
    fig, ax = plt.subplots(figsize=(14, 9))
    sns.set(style="whitegrid")

    scenario_labels_plotted = set()
    no_data = True

    for scenario_name in scenario_names:
        scenario_safe = scenario_name.replace("_", " ")
        rcp_value = scenario_name.split("-")[-1]
        label = f"RCP {rcp_value}"

        for pulse_year in pulse_years:
            file_path = os.path.join(output_folder, f"output_AGWP_N2O_{scenario_name}_Pulse_{pulse_year}.xlsx")
            if not os.path.exists(file_path):
                continue

            try:
                df = pd.read_excel(file_path)
                if df.empty:
                    continue
            except FileNotFoundError:
                continue

            color = get_scenario_color(rcp_value)
```

```
        no_data = False

        if label not in scenario_labels_plotted:
            ax.plot(df['Year'], df['AGWP_N2O'], label=label, color=color)
            scenario_labels_plotted.add(label)
        else:
            ax.plot(df['Year'], df['AGWP_N2O'], color=color, alpha=0.5)

    if not no_data:
        ax.set_xlabel("Pulse Year", fontsize=28)
        ax.set_ylabel("AGWP N₂O", fontsize=28)
        ax.set_title(f"AGWP N₂O for {iam_name} Scenarios", fontsize=26)
        ax.set_xlim(2025, 2200)
        ax.set_xticks(list(range(2030, 2201, 30)))  # dynamic ticks every 30 years
        handles, labels = ax.get_legend_handles_labels()
        for handle in handles:
            handle.set_linewidth(3.5)
        ax.legend(handles, labels, loc='upper left', bbox_to_anchor=(1.05, 1), fontsize=22)
        plt.tick_params(axis='both', which='major', labelsize=22)
        plt.tight_layout()
        plt.show()


# === MAIN FUNCTION ===

def main():
    base_dir = os.path.join(os.getcwd(), "inputs")
    output_dir = os.path.join(os.getcwd(), "outputs", "GWP")

    iams = ["IMAGE", "REMIND", "MESSAGE", "GCAM4", "AIM"]
    pulse_years = list(range(2030, 2101))

    for iam in iams:
        re_years, scenario_names, re_values_n2o = load_re_data(os.path.join(base_dir, f"pRE_N2O_{iam}.xlsx"))
        _, _, re_values_co2 = load_re_data(os.path.join(base_dir, f"pRE_CO2_{iam}.xlsx"))
        _, _, re_values_ch4 = load_re_data(os.path.join(base_dir, f"pRE_CH4_{iam}.xlsx"))
        irf_values = load_irf_data(os.path.join(base_dir, "pIRF_N2O.xlsx"))

        for pulse_year in tqdm(pulse_years, desc=f"{iam}"):
            if pulse_year not in re_years:
                continue
            for scenario_index, scenario_name in enumerate(scenario_names):
                C1_values, C2_values, C3_values, T_s_values, T_d_values, factor1_values, factor2_values = calculate_rf(
                    pulse_year, re_years, re_values_n2o, re_values_ch4, re_values_co2, irf_values, scenario_index
                )
                if factor1_values is None or factor2_values is None:
                    continue

                AGWP_N2O_values = calculate_agwp_n2o(factor1_values, factor2_values)
                if not AGWP_N2O_values or len(AGWP_N2O_values) != 100:
                    continue

                save_results(output_dir, pulse_year, scenario_name, AGWP_N2O_values)

        plot_agwp_n2o(iam, scenario_names, pulse_years, output_dir)
```

```
if __name__ == "__main__":
    main()
```

Code #6. Generating GWP20 and GWP100 values for CH_4 and N_2O (Pulse years: 2030-2100)

```
# GWPTOOL — PUBLIC RELEASE VERSION (November 2025)
# Author: [Watanabe and Cherubini (2025)]
# Description:
#   Computes Global Warming Potential (GWP) for both CH4 and N2O
#   Remember to run this code only after generating all AGWPs for both CH4 and N2O

import os
import re
import pandas as pd
from tqdm import tqdm

def extract_key_parts(filename):
    """Extracts gas type, model_scenario name, and pulse year from the filename."""
    match = re.search(r'AGWP_(CH4|CO2|N2O)_(.*?)_Pulse_(\d{4})', filename)
    if match:
        gas_type = match.group(1)
        model_scenario = re.sub(r'[_\s]+', '_', match.group(2).strip())
        pulse_year = int(match.group(3))
        return gas_type, model_scenario, pulse_year
    return None, None, None

def find_matching_co2_file(gas_file, all_files):
    """Find the corresponding CO2 file for a CH4 or N2O file."""
    _, model_scenario, pulse_year = extract_key_parts(gas_file)
    for file in all_files:
        gas_type, file_model_scenario, file_pulse_year = extract_key_parts(file)
        if gas_type == 'CO2' and file_model_scenario == model_scenario and file_pulse_year == pulse_year:
            return file
    return None

def process_files(input_dir, output_dir):
    """Main processing function to compute and summarize GWP20 and GWP100 for CH4 and N2O."""
    all_files = os.listdir(input_dir)
    total_files = len([file for file in all_files if re.search(r'AGWP_(CH4|N2O)', file)])

    # Store scenario data separately for each gas and horizon
    scenario_data = {
        'CH4': {'GWP100': {}, 'GWP20': {}},
        'N2O': {'GWP100': {}, 'GWP20': {}}
    }
    all_years = set()

    with tqdm(total=total_files, desc="Processing Files", unit="file", ncols=100) as pbar:
        for file in all_files:
            gas_type, model_scenario, pulse_year = extract_key_parts(file)
            if gas_type in ['CH4', 'N2O']:
                pbar.set_postfix(file=file)
```

```
            matching_co2_file = find_matching_co2_file(file, all_files)

            if matching_co2_file:
                agwp_gas_df = pd.read_excel(os.path.join(input_dir, file))
                agwp_co2_df = pd.read_excel(os.path.join(input_dir, matching_co2_file))

                calendar_col = agwp_gas_df.columns[0]
                gas_values = pd.to_numeric(agwp_gas_df.iloc[:, 1], errors='coerce')
                co2_values = pd.to_numeric(agwp_co2_df.iloc[:, 1], errors='coerce')

                # Extract GWP100 and GWP20
                gas_value_100 = gas_values.iloc[-1]
                gas_value_20 = gas_values.iloc[19] if len(gas_values) > 19 else gas_values.iloc[-1]

                co2_value_100 = co2_values.iloc[-1]
                co2_value_20 = co2_values.iloc[19] if len(co2_values) > 19 else co2_values.iloc[-1]

                if co2_value_100 == 0 or pd.isna(co2_value_100):
                    continue

                gwp_100 = gas_value_100 / co2_value_100
                gwp_20 = gas_value_20 / co2_value_20 if co2_value_20 != 0 else None

                year_key = pulse_year
                all_years.add(year_key)

                # Store GWP values
                for horizon, value in zip(['GWP100', 'GWP20'], [gwp_100, gwp_20]):
                    if model_scenario not in scenario_data[gas_type][horizon]:
                        scenario_data[gas_type][horizon][model_scenario] = {}
                    if year_key not in scenario_data[gas_type][horizon][model_scenario]:
                        scenario_data[gas_type][horizon][model_scenario][year_key] = []
                    if value is not None:
                        scenario_data[gas_type][horizon][model_scenario][year_key].append(round(value, 1))

                # Export individual file results
                result_df = pd.DataFrame({
                    calendar_col: [pulse_year],
                    f'GWP_{gas_type}_100': [gwp_100],
                    f'GWP_{gas_type}_20': [gwp_20]
                })
                output_filename = file.replace('AGWP_', f'GWP_')
                result_df.to_excel(os.path.join(output_dir, output_filename), index=False)

            pbar.update(1)

    # Export summary tables
    all_years = sorted(all_years)

    def create_summary_df(scenario_horizon_data):
        row_labels = sorted(scenario_horizon_data.keys())
        table_data = []
        for scenario in row_labels:
            table_row = []
            for year in all_years:
                values = scenario_horizon_data[scenario].get(year, [])
```

```
            table_row.append(round(sum(values)/len(values),1) if values else '')
        table_data.append(table_row)
    return pd.DataFrame(table_data, columns=all_years, index=row_labels)

  for gas in ['CH4', 'N2O']:
    for horizon in ['GWP100', 'GWP20']:
        df = create_summary_df(scenario_data[gas][horizon])
        excel_filename = os.path.join(output_dir, f"{horizon}_{gas}_table.xlsx")
        df.to_excel(excel_filename, index_label="Scenario")

# Define input and output folders
input_dir = os.path.join('outputs', 'GWP')
output_dir = input_dir

# Run the process
process_files(input_dir, output_dir)
```

Code #7. AGTP of CO_2 for multiple IAM-SSP-RCP scenarios

```
# AGTP_CO2 TOOL — REFACTORED VERSION (November 2025)
# Description:
#   Computes Absolute Global Temperature Potential (AGTP) for CO2
#   using IAM-based RE and IRF data.
#   Inputs: /inputs folder
#   Outputs: /outputs/GTP folder

import os
import pandas as pd
import matplotlib.pyplot as plt
import seaborn as sns
from tqdm import tqdm
import matplotlib.ticker as ticker

# ---------------------------------------------------------------------------
# GLOBAL SETTINGS
# ---------------------------------------------------------------------------
plt.rcParams["font.family"] = "DejaVu Sans"

# Color palette
full_palette = sns.color_palette("viridis", 256)
palette = [full_palette[i] for i in [0, 60, 130, 255]]
scenario_colors = {'2.6': palette[0], '4.5': palette[1], '6.0': palette[2], '8.5': palette[3]}

def get_scenario_color(scenario_name):
  for key, color in scenario_colors.items():
    if scenario_name.endswith(key):
        return color
  return '#7f7f7f'  # default gray

# ---------------------------------------------------------------------------
# DATA LOADING
# ---------------------------------------------------------------------------
def load_re_data(file_path):
```

```
    try:
        df_re = pd.read_excel(file_path, sheet_name="Sheet1")
    except FileNotFoundError:
        print(f"Error: RE file not found at {file_path}")
        return None, None, None
    re_years = df_re.iloc[:, 0].astype(int).tolist()
    scenario_names = df_re.columns[1:].tolist()
    re_data = df_re.iloc[:, 1:].values.tolist()
    return re_years, scenario_names, re_data

def load_irf_data(file_path, expected_scenarios):
    try:
        df_irf = pd.read_excel(file_path, sheet_name="Sheet1")
    except FileNotFoundError:
        print(f"Error: IRF file not found at {file_path}")
        return None, None
    irf_years = df_irf.iloc[:, 0].astype(int).tolist()
    df_irf = df_irf.iloc[:, :len(expected_scenarios) + 1]
    irf_data = df_irf.iloc[:, 1:].values.tolist()
    return irf_years, irf_data

# ---------------------------------------------------------------------------
# COMPUTATION FUNCTIONS
# ---------------------------------------------------------------------------
def calculate_rf(pulse_year, re_years, re_values, irf_values, n_years=100):
    """Compute radiative forcing (RF) for AGTP calculation."""
    try:
        pulse_year_index = re_years.index(pulse_year)
    except ValueError:
        print(f"Error: Pulse Year {pulse_year} not found in RE data.")
        return None

    re_val = re_values[pulse_year_index]
    integration_years = min(len(irf_values), n_years)

    rf_values = [
        re_val * irf_values[i] * 1e9 * 1e-6 * 28.97 / 44.01 / 5.13252
        for i in range(integration_years)
    ]
    return rf_values

def calculate_temperature_and_agtp(rf_values):
    """Compute surface temperature, deep temperature, and AGTP values."""
    T_s = [0]
    T_d = [0]

    for t in range(1, len(rf_values)):
        T_s_prev = T_s[t - 1]
        T_d_prev = T_d[t - 1]
        rf_value = rf_values[t - 1] if t > 1 else rf_values[0]

        T_s_new = (T_s_prev + rf_value / 7.7 - 1.31 * T_s_prev / 7.7 -
                   1.03 * 0.88 / 1.03 * (T_s_prev - T_d_prev) / 7.7)
        T_d_new = T_d_prev + (0.88 / 1.03) * (T_s_prev - T_d_prev) / 147

        T_s.append(T_s_new)
```

```
        T_d.append(T_d_new)

    AGTP = [(T_s[t] + T_s[t + 1]) / 2 for t in range(len(T_s) - 1)]
    last_slope = T_s[-1] - T_s[-2]
    AGTP.append(AGTP[-1]  + last_slope / 2)

    return T_s[1:], T_d[1:], AGTP

def create_agtp_table(pulse_year, agtp_values):
    """Create AGTP table (Calendar Year vs AGTP)."""
    table_years = [pulse_year + i for i in range(len(agtp_values))]
    return [[table_years[i], agtp_values[i]] for i in range(len(agtp_values))]

def save_output(file_path, data, headers):
    os.makedirs(os.path.dirname(file_path),  exist_ok=True)
    df = pd.DataFrame(data, columns=headers)
    df.to_excel(file_path,  index=False)

# ---------------------------------------------------------------------------
# PROCESSING
# ---------------------------------------------------------------------------
def process_iam(iam_name,  input_folder, output_folder, n_years=100):
    print(f"\nProcessing IAM: {iam_name}")

    re_file_path = os.path.join(input_folder, f"pRE_CO2_{iam_name}.xlsx")
    irf_file_path  = os.path.join(input_folder, "pIRF_CO2.xlsx")

    re_years, scenario_names, re_data = load_re_data(re_file_path)
    if re_years is None:
        return
    _, irf_data = load_irf_data(irf_file_path,  scenario_names)
    if irf_data is None:
        return

    pulse_years = list(range(2030,  2101))
    total_iters = len(scenario_names) * len(pulse_years)

    with tqdm(total=total_iters, desc=f"{iam_name}", unit="iteration") as pbar:
        for scenario_idx, scenario_name in enumerate(scenario_names):
            for pulse_year in pulse_years:
                re_values = [row[scenario_idx]  for row in re_data]
                irf_values  = [row[scenario_idx]  for row in irf_data]

                rf_values = calculate_rf(pulse_year, re_years, re_values, irf_values, n_years=n_years)
                if rf_values is None:
                    pbar.update(1)
                    continue

                _, _, AGTP  = calculate_temperature_and_agtp(rf_values)
                agtp_table = create_agtp_table(pulse_year, AGTP)

                scenario_safe = scenario_name.replace(" ", "_")
                out_file = os.path.join(output_folder, f"output_AGTP_CO2_{scenario_safe}_Pulse_{pulse_year}.xlsx")
                save_output(out_file, agtp_table, ['Calendar Year',  'AGTP_CO2'])

                pbar.update(1)
```

```
# ---------------------------------------------------------------------------
# PLOTTING
# ---------------------------------------------------------------------------
def plot_agtp(iam_name, scenario_names, pulse_years, output_folder, n_years=100):
    fig, ax = plt.subplots(figsize=(19, 9))
    sns.set(style="whitegrid")
    plotted_labels = set()
    max_y_value = 0

    for scenario_name in scenario_names:
        scenario_safe = scenario_name.replace(" ", "_")
        color = get_scenario_color(scenario_name)

        for pulse_year in pulse_years:
            file_path = os.path.join(output_folder, f"output_AGTP_CO2_{scenario_safe}_Pulse_{pulse_year}.xlsx")
            if not os.path.exists(file_path):
                continue
            df = pd.read_excel(file_path)
            df = df[df['Calendar Year'] <= pulse_year + n_years - 1]
            max_y_value = max(max_y_value, df['AGTP_CO2'].max())

            if scenario_name not in plotted_labels:
                ax.plot(df['Calendar Year'], df['AGTP_CO2'], label=scenario_name, color=color, linewidth=3)
                plotted_labels.add(scenario_name)
            else:
                ax.plot(df['Calendar Year'], df['AGTP_CO2'], color=color, alpha=0.4, linewidth=2)

    # Labels and limits
    ax.set_xlabel("Calendar Year", fontsize=30)
    ax.set_ylabel("AGTP, CO2, nK", fontsize=30)
    ax.tick_params(axis='both', which='major', labelsize=28)
    ax.set_xlim(2025, 2200)
    ax.xaxis.set_minor_locator(ticker.MultipleLocator(5))

    # Y-axis scientific notation
    ax.set_ylim(0, max_y_value * 1.1 if max_y_value > 0 else 1)
    ax.yaxis.set_minor_locator(ticker.MultipleLocator(max_y_value / 10 if max_y_value > 0 else 0.1))
    ax.yaxis.get_offset_text().set_fontsize(28)
    ax.ticklabel_format(axis='y', style='scientific', scilimits=(0,0))

    # Grid and legend
    ax.grid(which='both', linestyle='--', linewidth=0.5, alpha=0.7)
    ax.legend(fontsize=20)
    plt.tight_layout()
    plt.show()

# ---------------------------------------------------------------------------
# MAIN
# ---------------------------------------------------------------------------
def main():
    input_folder = "inputs"
    output_folder = os.path.join("outputs", "GTP")
    n_years = 100

    iams = ["IMAGE", "REMIND", "MESSAGE", "GCAM4", "AIM"]
```

```
    pulse_years = list(range(2030, 2101))

    for iam in iams:
        process_iam(iam, input_folder, output_folder, n_years=n_years)
        re_file_path = os.path.join(input_folder, f"pRE_CO2_{iam}.xlsx")
        re_years, scenario_names, _ = load_re_data(re_file_path)
        if scenario_names:
            plot_agtp(iam, scenario_names, pulse_years, output_folder, n_years=n_years)

if __name__ == "__main__":
    main()
```

Code #8. AGTP of CH_4 for multiple IAM-SSP-RCP scenarios

```
# AGTP_CH4 TOOL — VERSION (November 2025)
# Description:
#   Computes Absolute Global Temperature Potential (AGTP) for CH4
#   using IAM-based RE and IRF data.
#   Inputs: /inputs folder
#   Outputs: /outputs/GTP folder

import numpy as np
import os
import matplotlib.pyplot as plt
import pandas as pd
from tqdm import tqdm # Progress bar library
import seaborn as sns

# === Data Loading Functions ===
def load_re_data(file_path):
    df_re = pd.read_excel(file_path, sheet_name="Sheet1")
    re_years = df_re.iloc[:, 0].astype(int).tolist()
    scenario_names = df_re.columns[1:].tolist()
    re_values = df_re.iloc[:, 1:].values
    return re_years, scenario_names, re_values

def load_irf_data(file_path, expected_scenarios):
    df_irf = pd.read_excel(file_path, sheet_name="Sheet1")
    df_irf = df_irf.iloc[:, :len(expected_scenarios) + 1]
    irf_values = df_irf.iloc[:, 1:].values
    return irf_values

# === Core Calculation Functions ===
def calculate_rf(pulse_year, re_years, re_values_ch4, re_values_co2, irf_values, scenario_index):
    try:
        pulse_year_index = re_years.index(pulse_year)
    except ValueError:
        print(f"Error: Pulse Year {pulse_year} not found in RE data.")
        return None

    # ✅ Removed unnecessary check for 100-year data
```

```
rf_values, factor2_values, conc_values = [], [], []
C1_values, C2_values, C3_values = [], [], []
T_s_values, T_d_values = [], []
factor1_values = []
T_s_prime_values, T_d_prime_values = [], []

constant = 10**-6 * 28.97 / 16.04 * 1 / 5.13252

T_s, T_d = [0] * 102, [0] * 102
T_s_prime, T_d_prime = [0] * 102, [0] * 102
C1, C2, C3 = [0] * 102, [0] * 102, [0] * 102
factor2 = [0] * 102

# Use RE from pulse year only
fixed_re_ch4 = re_values_ch4[pulse_year_index, scenario_index]
fixed_re_co2 = re_values_co2[pulse_year_index, scenario_index]

for t in range(1, 101):
    re_value = fixed_re_ch4
    irf_value = irf_values[t - 1, scenario_index]

    factor1 = (re_value + 0.00014 + 0.00004) * 10**9 * (irf_value * constant)
    factor1_values.append(factor1)

    T_s[t] = T_s[t - 1] + factor1 / 7.7 + (-1.31) * T_s[t - 1] / 7.7 - 1.03 * (0.88/1.03) * (T_s[t - 1] - T_d[t - 1]) / 7.7
    T_d[t] = T_d[t - 1] + (0.88/1.03) * (T_s[t - 1] - T_d[t - 1]) / 147

    T_s_values.append(T_s[t])
    T_d_values.append(T_d[t])

    C1[t] = T_s[t] * 10**-9 * 11.06 * 10**9 * 0.6368 + C1[t - 1] * np.exp(-1 / 2.376)
    C1_values.append(C1[t])

    C2[t] = T_s[t] * 10**-9 * 11.06 * 10**9 * 0.3322 + C1[t - 1] * np.exp(-1 / 30.14)
    C3[t] = T_s[t] * 10**-9 * 11.06 * 10**9 * 0.031 + C1[t - 1] * np.exp(-1 / 490.1)
    C2_values.append(C2[t])
    C3_values.append(C3[t])

    factor2[t] = (C1[t] + C2[t] + C3[t]) * 10**-6 * 28.97 / 44.01 * 1 / 5.13252 * fixed_re_co2 * 10**9
    factor2_values.append(factor2[t])

    T_s_prime[t] = T_s_prime[t - 1] + factor2[t] / 7.7 - 1.31 * T_s_prime[t - 1] / 7.7 - 1.03 * (0.88 / 1.03) * (T_s_prime[t
- 1] - T_d_prime[t - 1]) / 7.7
    T_d_prime[t] = T_d_prime[t - 1] + (0.88 / 1.03) * (T_s_prime[t - 1] - T_d_prime[t - 1]) / 147
    T_s_prime_values.append(T_s_prime[t])
    T_d_prime_values.append(T_d_prime[t])

# t = 101 extrapolation
t = 101
re_value = fixed_re_ch4
irf_value = irf_values[t - 1, scenario_index]

factor1 = (re_value + 0.00014 + 0.00004) * 10**9 * (irf_value * constant)
factor1_values.append(factor1)

T_s[t] = T_s[t - 1] + factor1 / 7.7 + (-1.31) * T_s[t - 1] / 7.7 - 1.03 * (0.88/1.03) * (T_s[t - 1] - T_d[t - 1]) / 7.7
```

```
    T_d[t] = T_d[t - 1] + (0.88/1.03)  * (T_s[t - 1] - T_d[t - 1]) / 147
    T_s_values.append(T_s[t])
    T_d_values.append(T_d[t])

    C1[t] = T_s[t] * 10**-9 * 11.06 * 10**9 * 0.6368 + C1[t - 1] * np.exp(-1 / 2.376)
    C1_values.append(C1[t])
    C2[t] = T_s[t] * 10**-9 * 11.06 * 10**9 * 0.3322 + C1[t - 1] * np.exp(-1 / 30.14)
    C3[t] = T_s[t] * 10**-9 * 11.06 * 10**9 * 0.031 + C1[t - 1] * np.exp(-1 / 490.1)
    C2_values.append(C2[t])
    C3_values.append(C3[t])

    factor2[t] = (C1[t] + C2[t] + C3[t]) * 10**-6 * 28.97 / 44.01 * 1 / 5.13252 * fixed_re_co2 * 10**9
    factor2_values.append(factor2[t])

    T_s_prime[t] = T_s_prime[t - 1] + factor2[t] / 7.7 - 1.31 * T_s_prime[t - 1] / 7.7 - 1.03 * (0.88 / 1.03) * (T_s_prime[t -
1] - T_d_prime[t - 1]) / 7.7
    T_d_prime[t] = T_d_prime[t - 1] + (0.88 / 1.03) * (T_s_prime[t - 1] - T_d_prime[t - 1]) / 147
    T_s_prime_values.append(T_s_prime[t])
    T_d_prime_values.append(T_d_prime[t])

    return C1_values, C2_values, C3_values, T_s_values, T_d_values, factor1_values, factor2_values, T_s_prime_values,
T_d_prime_values

def calculate_agtp_ch4(T_s_values, T_s_prime_values):
    AGTP_CH4_values = []
    for t in range(len(T_s_values) - 1):
        avg_T_s = (T_s_values[t] + T_s_values[t + 1]) / 2
        avg_T_s_prime = (T_s_prime_values[t] + T_s_prime_values[t + 1]) / 2
        AGTP_CH4_values.append(avg_T_s + avg_T_s_prime)

    # Linear extrapolation for t=101
    if len(AGTP_CH4_values) >= 100:
        x = np.array([99, 100])
        y = np.array(AGTP_CH4_values[-2:])
        slope, intercept = np.polyfit(x, y, 1)
        AGTP_CH4_values.append(slope * 101 + intercept)

    return AGTP_CH4_values

# === Save Results ===
def save_results(output_dir, pulse_year, scenario_name, AGTP_CH4_values):
    if len(AGTP_CH4_values) != 101:
        print(f"Warning: AGTP_CH4 length {len(AGTP_CH4_values)} instead of 101")
        return
    AGTP_CH4_values = AGTP_CH4_values[:-1]
    file_path = os.path.join(output_dir, f"output_AGTP_CH4_{scenario_name}_Pulse_{pulse_year}.xlsx")
    df_output = pd.DataFrame({
        "Calendar Year": list(range(pulse_year, pulse_year + 100)),
        "AGTP_CH4": AGTP_CH4_values
    })
    df_output.to_excel(file_path, index=False)

# === Plotting ===
full_palette = sns.color_palette("viridis", 256)
palette = [full_palette[i] for i in [0, 60, 130, 255]]
scenario_colors = {'2.6': palette[0], '4.5': palette[1], '6.0': palette[2], '8.5': palette[3]}
```

```
def get_scenario_color(scenario_name):
    for key, color in scenario_colors.items():
        if scenario_name.endswith(key):
            return color
    return '#7f7f7f'

def plot_agtp_ch4(agtp_values, scenario_names, pulse_years, iam_name):
    fig, ax = plt.subplots(figsize=(19, 9))
    sns.set(style="whitegrid")
    scenario_labels_plotted = set()
    no_data = True

    # Define SSP mapping
    iam_ssp_map = {
        "IMAGE": "SSP1",
        "MESSAGE": "SSP2",
        "AIM": "SSP3",
        "GCAM4": "SSP4",
        "REMIND": "SSP5"
    }
    ssp_label = iam_ssp_map.get(iam_name, "SSPX")  # default if not found

    for i, scenario_name in enumerate(scenario_names):
        for j, pulse_year in enumerate(pulse_years):
            agtp_values_for_scenario = agtp_values[i][j]
            pulse_year_adjusted = [pulse_year + k for k in range(len(agtp_values_for_scenario))]
            rcp_value = scenario_name.split(" - ")[-1]  # extract RCP number
            label = f"{iam_name} - {ssp_label} - {rcp_value}"  # IAM-SSPX-Y.Y format
            color = get_scenario_color(scenario_name)
            no_data = False
            if label not in scenario_labels_plotted:
                ax.plot(pulse_year_adjusted, agtp_values_for_scenario, label=label, color=color, linestyle='-', linewidth=3)
                scenario_labels_plotted.add(label)
            else:
                ax.plot(pulse_year_adjusted, agtp_values_for_scenario, color=color, alpha=0.5, linestyle='-', linewidth=3)

    if no_data:
        print("No data was found for plotting.")
    else:
        ax.set_xlabel("Calendar Year", fontsize=28)
        ax.set_ylabel("AGTP, CH4, nK", fontsize=28)
        ax.tick_params(axis='both', which='major', labelsize=28)
        ax.set_xticks(range(2040, 2201, 20))
        ax.set_xlim(2025, 2201)
        ax.ticklabel_format(style='scientific', axis='y', scilimits=(0,0))
        ax.yaxis.get_offset_text().set_fontsize(20)

        # Legend inside plot
        legend = ax.legend(loc='upper right', fontsize=24, frameon=True)
        for handle in legend.legendHandles:
            handle.set_linewidth(3)

        ax.grid(which='both', linestyle='--', linewidth=0.5, alpha=0.7)
        plt.tight_layout()
        plt.show()
```

```
# === IAM Processing ===
def process_iam(iam_name):
    print(f"\nProcessing IAM: {iam_name}")
    base_input_dir = os.path.join(os.getcwd(), "inputs")
    output_dir = os.path.join(os.getcwd(), "outputs", "GTP")
    os.makedirs(output_dir, exist_ok=True)

    re_years, scenario_names, re_values_ch4 = load_re_data(os.path.join(base_input_dir, f"pRE_CH4_{iam_name}.xlsx"))
    _, _, re_values_co2 = load_re_data(os.path.join(base_input_dir, f"pRE_CO2_{iam_name}.xlsx"))
    irf_values = load_irf_data(os.path.join(base_input_dir, "pIRF_CH4.xlsx"), scenario_names)

    pulse_years = list(range(2030, 2101))
    total_iterations = len(scenario_names) * len(pulse_years)
    agtp_values = [[] for _ in range(len(scenario_names))]

    with tqdm(total=total_iterations, desc=f"Processing {iam_name}", unit="iteration") as pbar:
        for pulse_year in pulse_years:
            for scenario_index, scenario_name in enumerate(scenario_names):
                result = calculate_rf(pulse_year, re_years, re_values_ch4, re_values_co2, irf_values, scenario_index)
                if result is None:
                    print(f"Skipping scenario {scenario_name} due to error.")
                    pbar.update(1)
                    continue
                C1_values, C2_values, C3_values, T_s_values, T_d_values, factor1_values, factor2_values, T_s_prime_values,
T_d_prime_values = result
                AGTP_CH4_values = calculate_agtp_ch4(T_s_values, T_s_prime_values)
                save_results(output_dir, pulse_year, scenario_name, AGTP_CH4_values)
                agtp_values[scenario_index].append(AGTP_CH4_values)
                pbar.update(1)

    # Corrected call with iam_name
    plot_agtp_ch4(agtp_values, scenario_names, pulse_years, iam_name)


def main():
    iams = ["IMAGE", "REMIND", "MESSAGE", "GCAM4", "AIM"]
    for iam in iams:
        process_iam(iam)

if __name__ == "__main__":
    main()
```

Code #9. AGTP of N_2O for multiple IAM-SSP-RCP scenarios

```
# AGTP_N20 TOOL — VERSION (November 2025)
# Description:
#   Computes Absolute Global Temperature Potential (AGTP) for N2O
#   using IAM-based RE and IRF data.
#   Inputs: /inputs folder
#   Outputs: /outputs/GTP folder
```

```
import pandas as pd
import numpy as np
import os
import matplotlib.pyplot as plt
from tqdm import tqdm
import seaborn as sns

# === Data Loading Functions ===
def load_re_data(file_path):
    df_re = pd.read_excel(file_path, sheet_name="Sheet1")
    re_years = df_re.iloc[:, 0].astype(int).tolist()
    scenario_names = df_re.columns[1:].tolist()
    re_values = df_re.iloc[:, 1:].values
    return re_years, scenario_names, re_values

def load_irf_data(file_path, expected_scenarios):
    df_irf = pd.read_excel(file_path, sheet_name="Sheet1")
    df_irf = df_irf.iloc[:, :len(expected_scenarios) + 1]
    irf_values = df_irf.iloc[:, 1:].values
    return irf_values

# === Calculation Functions ===
def calculate_rf(pulse_year, re_years, re_values_n2o, re_values_ch4, re_values_co2, irf_values, scenario_index):
    try:
        pulse_year_index = re_years.index(pulse_year)
    except ValueError:
        print(f"Error: Pulse Year {pulse_year} not found in RE data.")
        return None

    n_steps = irf_values.shape[0]

    C1_values = [0] * n_steps
    C2_values = [0] * n_steps
    C3_values = [0] * n_steps
    T_s_values = [0] * n_steps
    T_d_values = [0] * n_steps
    factor1_values = [0] * n_steps
    factor2_values = [0] * n_steps
    T_s_prime = [0] * n_steps
    T_d_prime = [0] * n_steps
    constant = 10**-6 * 28.97 / 44.013 * 1 / 5.13252

    fixed_re_n2o = re_values_n2o[pulse_year_index, scenario_index]
    fixed_re_ch4 = re_values_ch4[pulse_year_index, scenario_index]
    fixed_re_co2 = re_values_co2[pulse_year_index, scenario_index]

    for t in range(n_steps):
        irf_value = irf_values[t, scenario_index]
        factor1 = (fixed_re_n2o + 0.00055 - 1.7 * (fixed_re_ch4 + 0.00014 + 0.00004)) * 10**9 * (irf_value * constant)
        factor1_values[t] = factor1

        if t > 0:
            T_s_values[t] = T_s_values[t - 1] + factor1 / 7.7 + (-1.31) * T_s_values[t - 1] / 7.7 - 1.03 * (0.88 / 1.03) *
(T_s_values[t - 1] - T_d_values[t - 1]) / 7.7
            T_d_values[t] = T_d_values[t - 1] + (0.88 / 1.03) * (T_s_values[t - 1] - T_d_values[t - 1]) / 147
```

```
        C1_values[t] = T_s_values[t] * 10**-9 * 11.06 * 10**9 * 0.6368 + C1_values[t - 1] * np.exp(-1 / 2.376)
        C2_values[t] = T_s_values[t] * 10**-9 * 11.06 * 10**9 * 0.3322 + C2_values[t - 1] * np.exp(-1 / 30.14)
        C3_values[t] = T_s_values[t] * 10**-9 * 11.06 * 10**9 * 0.031 + C3_values[t - 1] * np.exp(-1 / 490.1)

        factor2_values[t] = (C1_values[t] + C2_values[t] + C3_values[t]) * constant * fixed_re_co2 * 10**9

        T_s_prime[t] = T_s_prime[t - 1] + factor2_values[t] / 7.7 - 1.31 * T_s_prime[t - 1] / 7.7 - 1.03 * (0.88 / 1.03) *
(T_s_prime[t - 1] - T_d_prime[t - 1]) / 7.7
        T_d_prime[t] = T_d_prime[t - 1] + (0.88 / 1.03) * (T_s_prime[t - 1] - T_d_prime[t - 1]) / 147

    return C1_values, C2_values, C3_values, T_s_values, T_d_values, T_s_prime, T_d_prime, factor1_values,
factor2_values

def calculate_agtp_n2o(T_s_values, T_s_prime_values):
    AGTP_N2O_values = []
    for t in range(len(T_s_values) - 1):
        avg_T_s = (T_s_values[t] + T_s_values[t + 1]) / 2
        avg_T_s_prime = (T_s_prime_values[t] + T_s_prime_values[t + 1]) / 2
        AGTP_N2O_values.append(avg_T_s + avg_T_s_prime)

    while len(AGTP_N2O_values) < 100:
        slope = AGTP_N2O_values[-1] - AGTP_N2O_values[-2]
        AGTP_N2O_values.append(AGTP_N2O_values[-1] + slope)

    return AGTP_N2O_values[:100]

def save_results(output_dir, pulse_year, scenario_name, AGTP_N2O_values):
    os.makedirs(output_dir, exist_ok=True)
    df_output = pd.DataFrame({
        "Calendar Year": list(range(pulse_year, pulse_year + len(AGTP_N2O_values))),
        "AGTP_N2O": AGTP_N2O_values
    })
    df_output.to_excel(os.path.join(output_dir, f"output_AGTP_N2O_{scenario_name}_Pulse_{pulse_year}.xlsx"),
index=False)

# === Plotting ===
full_palette = sns.color_palette("viridis", 256)
palette = [full_palette[i] for i in [0, 60, 130, 255]]
scenario_colors = {'2.6': palette[0], '4.5': palette[1], '6.0': palette[2], '8.5': palette[3]}

def get_scenario_color(scenario_name):
    for key, color in scenario_colors.items():
        if scenario_name.endswith(key):
            return color
    return '#7f7f7f'

def plot_agtp_n2o(agtp_values, scenario_names, pulse_years, iam_name):
    fig, ax = plt.subplots(figsize=(19, 9))
    sns.set(style="whitegrid")
    scenario_labels_plotted = set()
    no_data = True

    iam_ssp_map = {
        "IMAGE": "SSP1",
        "MESSAGE": "SSP2",
        "AIM": "SSP3",
```

```
        "GCAM4": "SSP4",
        "REMIND": "SSP5"
    }
    ssp_label = iam_ssp_map.get(iam_name, "SSPX")

    for i, scenario_name in enumerate(scenario_names):
        for j, pulse_year in enumerate(pulse_years):
            agtp_values_for_scenario = agtp_values[i][j]
            pulse_year_adjusted = [pulse_year + k for k in range(len(agtp_values_for_scenario))]
            rcp_value = scenario_name.split(" - ")[-1]
            label = f"{iam_name} - {ssp_label} - {rcp_value}"
            color = get_scenario_color(scenario_name)
            no_data = False
            if label not in scenario_labels_plotted:
                ax.plot(pulse_year_adjusted, agtp_values_for_scenario, label=label, color=color, linestyle='-', linewidth=3)
                scenario_labels_plotted.add(label)
            else:
                ax.plot(pulse_year_adjusted, agtp_values_for_scenario, color=color, alpha=0.5, linestyle='-', linewidth=3)

    if not no_data:
        ax.set_xlabel("Calendar Year", fontsize=28)
        ax.set_ylabel("AGTP, N2O, nK", fontsize=28)
        ax.tick_params(axis='both', which='major', labelsize=28)
        ax.set_xticks(range(2040, 2201, 20))
        ax.set_xlim(2025, 2201)
        ax.ticklabel_format(style='scientific', axis='y', scilimits=(0,0))
        ax.yaxis.get_offset_text().set_fontsize(20)

        legend = ax.legend(loc='upper right', fontsize=24, frameon=True)
        for handle in legend.legendHandles:
            handle.set_linewidth(3)

        ax.grid(which='both', linestyle='--', linewidth=0.5, alpha=0.7)
        plt.tight_layout()
        plt.show()

# === IAM Processing ===
def process_iam(iam_name):
    print(f"\nProcessing IAM: {iam_name}")
    input_dir = "inputs"
    output_dir = os.path.join("outputs", "GTP")
    os.makedirs(output_dir, exist_ok=True)

    re_years, scenario_names, re_values_n2o = load_re_data(os.path.join(input_dir, f"pRE_N2O_{iam_name}.xlsx"))
    _, _, re_values_co2 = load_re_data(os.path.join(input_dir, f"pRE_CO2_{iam_name}.xlsx"))
    _, _, re_values_ch4 = load_re_data(os.path.join(input_dir, f"pRE_CH4_{iam_name}.xlsx"))
    irf_values = load_irf_data(os.path.join(input_dir, "pIRF_N2O.xlsx"), scenario_names)

    pulse_years = list(range(2030, 2101))
    agtp_values_all = [[] for _ in range(len(scenario_names))]

    total_iterations = len(scenario_names) * len(pulse_years)
    with tqdm(total=total_iterations, desc=f"Processing {iam_name}", unit="iteration") as pbar:
        for pulse_year in pulse_years:
            for scenario_idx, scenario_name in enumerate(scenario_names):
```

```
            results = calculate_rf(pulse_year, re_years, re_values_n2o, re_values_ch4, re_values_co2, irf_values,
scenario_idx)
            if results is None:
                pbar.update(1)
                continue
            C1_values, C2_values, C3_values, T_s_values, T_d_values, T_s_prime_values, T_d_prime_values,
factor1_values, factor2_values = results
            AGTP_N2O_values = calculate_agtp_n2o(T_s_values, T_s_prime_values)
            save_results(output_dir, pulse_year, scenario_name, AGTP_N2O_values)
            agtp_values_all[scenario_idx].append(AGTP_N2O_values)
            pbar.update(1)

    plot_agtp_n2o(agtp_values_all, scenario_names, pulse_years, iam_name)

def main():
    iams = ["IMAGE", "REMIND", "MESSAGE", "GCAM4", "AIM"]
    for iam in iams:
        process_iam(iam)

if __name__ == "__main__":
    main()
```

Code #10. Generating GTP50 and GTP100 values for CH4 and N2O (Pulse years: 2030-2100)

```
# GTPTOOL — PUBLIC RELEASE VERSION (November 2025)
# Author: [Watanabe and Cherubini (2025)]
# Description:
#   Computes Global Temperature Change Potential (GTP) for both CH4 and N2O
#   Remember to run this code only after generating all AGTPs for both CH4 and N2O
#   Get Excel Tables for GTP100, GTP50 (2030-2100)

import os
import re
import pandas as pd
from tqdm import tqdm

def extract_key_parts(filename):
    match = re.search(r'AGTP_(CH4|CO2|N2O)_(.*?)_Pulse_(\d{4})', filename)
    if match:
        gas_type = match.group(1)
        model_scenario = re.sub(r'[_\s]+', '_', match.group(2).strip())
        pulse_year = int(match.group(3))
        return gas_type, model_scenario, pulse_year
    return None, None, None

def find_matching_co2_file(gas_file, all_files):
    _, model_scenario, pulse_year = extract_key_parts(gas_file)
    for file in all_files:
        gas_type, file_model_scenario, file_pulse_year = extract_key_parts(file)
        if gas_type == 'CO2' and file_model_scenario == model_scenario and file_pulse_year == pulse_year:
            return file
    return None

def process_files(input_dir, output_dir):
```

```
all_files = os.listdir(input_dir)
gas_files = [f for f in all_files if 'AGTP_CH4' in f or 'AGTP_N2O' in f]

scenario_data_50 = {'CH4': {}, 'N2O': {}}
scenario_data_100 = {'CH4': {}, 'N2O': {}}
all_years = set()

with tqdm(total=len(gas_files), desc="Processing Files", unit="file", ncols=100) as pbar:
    for file in gas_files:
        pbar.set_postfix(file=file)
        gas_type, model_scenario, pulse_year = extract_key_parts(file)
        matching_co2_file = find_matching_co2_file(file, all_files)

        if matching_co2_file:
            gas_df = pd.read_excel(os.path.join(input_dir, file))
            co2_df = pd.read_excel(os.path.join(input_dir, matching_co2_file))

            calendar_col = gas_df.columns[0]
            gas_values = pd.to_numeric(gas_df.iloc[:, 1], errors='coerce')
            co2_values = pd.to_numeric(co2_df.iloc[:, 1], errors='coerce')

            if len(gas_values) < 100 or len(co2_values) < 100:
                pbar.update(1)
                continue

            gas_value_50 = gas_values.iloc[49]
            co2_value_50 = co2_values.iloc[49]
            gas_value_100 = gas_values.iloc[99]
            co2_value_100 = co2_values.iloc[99]

            if co2_value_50 == 0 or pd.isna(co2_value_50) or co2_value_100 == 0 or pd.isna(co2_value_100):
                pbar.update(1)
                continue

            gtp_value_50 = gas_value_50 / co2_value_50
            gtp_value_100 = gas_value_100 / co2_value_100

            # Store GTP50
            if model_scenario not in scenario_data_50[gas_type]:
                scenario_data_50[gas_type][model_scenario] = {}
            scenario_data_50[gas_type][model_scenario][pulse_year] = gtp_value_50

            # Store GTP100
            if model_scenario not in scenario_data_100[gas_type]:
                scenario_data_100[gas_type][model_scenario] = {}
            scenario_data_100[gas_type][model_scenario][pulse_year] = gtp_value_100

            all_years.add(pulse_year)

            # Create individual output file
            result_df = pd.DataFrame({
                calendar_col: [pulse_year],
                f'GTP50_{gas_type}': [gtp_value_50],
                f'GTP100_{gas_type}': [gtp_value_100]
            })
            output_filename = file.replace(f'AGTP_{gas_type}', f'GTP_{gas_type}')
```

```
            result_df.to_excel(os.path.join(output_dir, output_filename), index=False)

        pbar.update(1)

    # Export summary tables
    all_years = sorted(all_years)

    def create_summary_df(scenario_data):
        table_data = []
        row_labels = sorted(scenario_data.keys())
        for scenario in row_labels:
            table_row = [scenario_data[scenario].get(year, '') for year in all_years]
            table_data.append(table_row)
        return pd.DataFrame(table_data, columns=all_years, index=row_labels)

    for gas in ['CH4', 'N2O']:
        gtp50_df = create_summary_df(scenario_data_50[gas])
        gtp50_df.to_excel(os.path.join(output_dir, f"GTP50_{gas}_table.xlsx"), index_label="Scenario")

        gtp100_df = create_summary_df(scenario_data_100[gas])
        gtp100_df.to_excel(os.path.join(output_dir, f"GTP100_{gas}_table.xlsx"), index_label="Scenario")


# Define input/output directories
input_dir = os.path.join('outputs', 'GTP')
output_dir = input_dir

# Run the process
process_files(input_dir, output_dir)
```
